# Novel risk genes and mechanisms implicated by exome sequencing of 2572 individuals with pulmonary arterial hypertension

**DOI:** 10.1186/s13073-019-0685-z

**Published:** 2019-11-14

**Authors:** Na Zhu, Michael W. Pauciulo, Carrie L. Welch, Katie A. Lutz, Anna W. Coleman, Claudia Gonzaga-Jauregui, Jiayao Wang, Joseph M. Grimes, Lisa J. Martin, Hua He, PAH Biobank Enrolling Centers’ Investigators, Russel Hirsch, R. James White, Marc Simon, David Badesch, Erika Rosenzweig, Charles Burger, Murali Chakinala, Thenappan Thenappan, Greg Elliott, Robert Simms, Harrison Farber, Robert Frantz, Jean Elwing, Nicholas Hill, Dunbar Ivy, James Klinger, Steven Nathan, Ronald Oudiz, Ivan Robbins, Robert Schilz, Terry Fortin, Jeffrey Wilt, Delphine Yung, Eric Austin, Ferhaan Ahmad, Nitin Bhatt, Tim Lahm, Adaani Frost, Zeenat Safdar, Zia Rehman, Robert Walter, Fernando Torres, Sahil Bakshi, Stephen Archer, Rahul Argula, Christopher Barnett, Raymond Benza, Ankit Desai, Veeranna Maddipati, Yufeng Shen, Wendy K. Chung, William C. Nichols

**Affiliations:** 1 Department of Pediatrics, Columbia University Medical Center, New York, NY USA; 2 Department of Systems Biology, Columbia University, New York, NY USA; 3 Division of Human Genetics, Cincinnati Children’s Hospital Medical Center, 3333 Burnet Avenue MLC 7016, Cincinnati, OH USA; 4 Department of Pediatrics, College of Medicine, University of Cincinnati, Cincinnati, OH USA; 5 Regeneron Genetics Center, Regeneron Pharmaceuticals, Tarrytown, NY USA; 6 Department of Biomedical Informatics, Columbia University, New York, NY USA; 7 Herbert Irving Comprehensive Cancer Center, Columbia University Medical Center, New York, NY USA; 8 Department of Medicine, Columbia University Medical Center, New York, NY USA

**Keywords:** Genetics, Pulmonary arterial hypertension, Exome sequencing, Case-control association testing

## Abstract

**Background:**

Group 1 pulmonary arterial hypertension (PAH) is a rare disease with high mortality despite recent therapeutic advances. Pathogenic remodeling of pulmonary arterioles leads to increased pulmonary pressures, right ventricular hypertrophy, and heart failure. Mutations in bone morphogenetic protein receptor type 2 and other risk genes predispose to disease, but the vast majority of non-familial cases remain genetically undefined.

**Methods:**

To identify new risk genes, we performed exome sequencing in a large cohort from the National Biological Sample and Data Repository for PAH (PAH Biobank, *n* = 2572). We then carried out rare deleterious variant identification followed by case-control gene-based association analyses. To control for population structure, only unrelated European cases (*n* = 1832) and controls (*n* = 12,771) were used in association tests. Empirical *p* values were determined by permutation analyses, and the threshold for significance defined by Bonferroni’s correction for multiple testing.

**Results:**

Tissue kallikrein 1 (*KLK1*) and gamma glutamyl carboxylase (*GGCX*) were identified as new candidate risk genes for idiopathic PAH (IPAH) with genome-wide significance. We note that variant carriers had later mean age of onset and relatively moderate disease phenotypes compared to bone morphogenetic receptor type 2 variant carriers. We also confirmed the genome-wide association of recently reported growth differentiation factor (*GDF2*) with IPAH and further implicate T-box 4 (*TBX4*) with child-onset PAH.

**Conclusions:**

We report robust association of novel genes *KLK1* and *GGCX* with IPAH, accounting for ~ 0.4% and 0.9% of PAH Biobank cases, respectively. Both genes play important roles in vascular hemodynamics and inflammation but have not been implicated in PAH previously. These data suggest new genes, pathogenic mechanisms, and therapeutic targets for this lethal vasculopathy.

## Background

Pulmonary arterial hypertension (PAH) is a progressive vascular disease characterized by proliferative remodeling, increased pulmonary pressures and resistance, and high mortality [1–4]. The disease is etiologically heterogeneous, classified as familial (FPAH) as a subset of heritable PAH, idiopathic (IPAH), associated with other medical conditions (APAH, including autoimmune connective tissue disorders (CTD), congenital heart disease (CHD), and portopulmonary disease (PoPH)), or induced by drugs and toxins (DTOX) [5]. Disease susceptibility includes genetic and environmental factors. Known risk genes underlie 70–80% of FPAH and ~ 10–40% of IPAH [6, 7]. However, the majority of non-familial cases remain genetically undefined.

Heterozygous germline mutations in bone morphogenetic protein receptor type 2 (*BMPR2*), a member of the transforming growth factor beta (TGF-β) superfamily, are the most common genetic cause of PAH [8–10]. Similar frequencies of *BMPR2* mutations are observed across patient ethnicities and are present in 60–80% of familial cases [11–14]. *BMPR2* mutations are observed in both child- and adult-onset PAH [14], and *BMPR2* mutation carriers exhibit a younger age of onset compared to non-carriers [7]. Mutations in the developmental transcription factor T-box 4 (*TBX4*) are more common in child-onset PAH, and de novo mutations in many different genes may explain ~ 19% of child-onset PAH [14]. Germline mutations in other genes are individually rare causes of PAH. These include other genes in the TGF-β/BMP signaling pathway [15], hereditary hemorrhagic telangiectasia (HHT) genes activin A receptor type II-like 1 (*ACVRL1*) and endoglin (*ENG*) [7], eukaryotic initiation translation factor (*EIF2AK4*) associated with pulmonary veno-occlusive disease (PVOD)/pulmonary capillary hemangiomatosis (PCH) [16, 17], caveolin-1 (*CAV1*) [18], and channel genes including potassium two pore domain channel (*KCNK3*) [19], ATP-binding cassette subfamily member 8 (*ABCC8*) [20], and voltage-dependent potassium channel 1.5 (*KCNA5*) [21].

New risk genes are emerging from large exome- and genome-wide sequencing studies. Rare mutations in SRY-related HMG-box transcription factor (*SOX17*), a key regulator of embryonic vasculogenesis, explain ~ 3.2% of APAH-CHD [22] and 0.7% of IPAH [22, 23]. The UK NIHR BioResource–Rare Diseases PAH Study, utilizing ~ 1000 PAH cases of primarily adult-onset IPAH, identified an ATPase gene (*ATP13A3*), growth differentiation factor 2 (*GDF2*; also known as *BMP9*), and *SOX17* as risk genes contributing to 0.8–1.1% of cases [23]. The low frequency of risk variants for each gene, except *BMPR2*, indicates that large numbers of individuals are required for further validation of rare risk genes and pathways, and to understand the natural history of each genetic subtype of PAH.

The National Biological Sample and Data Repository for PAH (aka PAH Biobank) is a resource of biological specimens as well as clinical and genetic data generated for 2900 group 1 PAH patients to serve as a resource to the research community to enable larger-scale PAH studies. Herein, we performed targeted PAH gene and whole exome sequencing of 2572 cases from the PAH Biobank to identify and characterize frequencies and mutations in known PAH risk genes, identify new risk genes, and identify correlations between risk genes and clinical phenotypes.

## Methods

### Participants

The PAH Biobank is housed and maintained at the Cincinnati Children’s Hospital Medical Center (CCHMC). Thirty-eight North American PH Centers participate in the PAH Biobank to identify and enroll patients meeting eligibility criteria. Each enrolling center also completes an electronic case report form with clinical data for each patient enrolled. Participants are diagnosed according to the World Health Organization PH group I classification [5], and the diagnosis of PAH is confirmed by medical record review including right heart catheterization. The cohort for this genetic analysis included 2534 singletons, 19 duos (proband and 1 unaffected parent), and 19 trios (proband and 2 unaffected biological parents). Written informed consent (and assent when appropriate) was obtained from participants or parents/legal guardians under a protocol approved by the institutional review board at CCHMC as well as those at each of the participating PH Centers. Written informed consent for publication was obtained at enrollment. The data and resources of the PAH Biobank are made available to the research community for hypothesis-driven projects via an application process (www.pahbiobank.org). A subset including 183 affected participants were included in previous publications from our group [14, 20, 22].

### Targeted sequencing and multiplex ligation-dependent probe amplification (MLPA)

After proper informed consent, blood samples were collected and shipped to CCHMC for processing and generation of genetic data including panel sequencing of up to 12 genes, SNP genotyping using the Illumina OMNI5-4 Beadchip, and limited MLPA dosage data. Targeted next-generation sequencing was performed with 250 ng DNA using the Illumina Tru-seq Custom Amplicon system (Illumina, USA) according to the manufacturer’s instructions. Custom amplicons were designed with Illumina’s DesignStudio for the coding sequence of *BMPR2*, *ACVRL1*, *ENG*, *CAV1*, *SMAD9*, *KCNK3*, and *EIF2AK4*, for which all participants were sequenced. *ABCC8*, *GDF2*, *KCNA5*, *SMAD4*, and *TBX4* were added to the panel later, and a subset of 739 were also sequenced for these genes. Each sample was sequenced using the Illumina MiSeq® instrument with paired-end 250 nucleotide read lengths. Demultiplexing, base calling, and alignment were executed using the default Illumina TruSeq Amplicon Workflow. Fastq files were aligned and visualized with NextGENe (SoftGenetics, USA). Variants were confirmed via Sanger sequencing on an ABI 3730xl DNA analyzer (Applied Biosystems, USA).

MLPA was performed with 100 ng of genomic DNA according to the manufacturer’s instructions using the P093 Salsa MLPA probe sets (MRC-Holland, Amsterdam, The Netherlands). This probe set includes probes for all exons of *BMPR2*, *ALK1*, and *ENG*. Probe amplification products were run on an ABI 3730xl DNA Analyzer using GS500 size standard (Applied Biosystems). MLPA peak data was imported into Coffalyser (MRC-Holland) for quality checks and dosage ratio analysis. A dosage ratio value of ≤ 0.7 was used as the boundary for deletions, and ≥ 1.35 was used as the boundary for duplications.

### Whole exome sequencing (WES)

Exome sequencing was performed for the entire cohort in collaboration with the Regeneron Genetics Center (RGC). In brief, genomic DNA was prepared with a customized reagent kit from Kapa Biosystems and captured using Integrated DNA Technologies xGen lockdown probes. All samples were sequenced on the Illumina HiSeq 2500 platform using v4 chemistry, generating 76 bp paired-end reads. 99.8% of the exome sequencing samples have read depth coverage ≥ 15× for 90% of the targeted regions (see Additional file 1: Figure S1).

### WES data analysis

We used a previously established bioinformatics procedure [24] to process and analyze exome sequence data. Specifically, we used BWA-MEM [25] to map and align paired-end reads to the human reference genome (version GRCh38/hg38), Picard MarkDuplicates to identify and flag PCR duplicates, and GATK HaplotypeCaller (version 3.5) [26, 27] recommended settings to call genetic variants. We used additional heuristic filters to minimize technical artifacts, excluding variants that met any of the following criteria: missingness > 10%, allele balance ≤ 25% [28], genotype quality < 60 for indels or < 90 for SNVs, cohort allele frequency ≥ 0.01, depth < 9, GATK MQ < 40, located in MUC or HLA genes, and located in segmental duplications with similarity ≥ 95%. We obtained gnomAD whole genome sequence (WGS, data release v2.02) as part of the controls. We applied the same heuristic filtering approach with additional exclusion criteria: not “PASS,” not located in xGen-captured protein coding region, VQSLOD ≤ − 5.5, and FS ≥ 35. We obtained WES data of unaffected parents from the Pediatric Cardiac Genomics Consortium (PCGC) [24] as “internal” controls. The internal control data was processed with the same pipeline as cases. For both panel and exome sequencing data, we used ANNOVAR [29] to aggregate variant annotation, allele frequencies (AF), and in silico predictions of deleteriousness. Rare variants were defined as AF ≤ 0.01% in both ExAC and gnomAD exome datasets (all ancestries). An exception was made for recessive inheritance of *EIF2AK4* variants [16, 17], in which the AF cutoff was ≤ 1%. Deleterious variants were defined as likely gene damaging (LGD, including premature stop-gain, frameshift indels, canonical splicing variants, and exon deletions) or predicted damaging missense with REVEL score > 0.5 (D-Mis), as previously described [22, 30]. For *EIF2AK4* variants, deleterious missense variants were defined by CADD score ≥ 20 since REVEL is not optimized to assess the deleteriousness of relatively common variants. CADD scores for all variants are provided in the variant tables as a reference [31]. Insertion/deletion variants were manually inspected using Integrative Genome Viewer (IGV).

### Statistical analysis

To identify novel risk genes, we performed a gene-based case-control association test comparing the frequency of rare deleterious variants in PAH cases with population controls. The controls consisted of gnomAD WGS subjects and unaffected parents from the Pediatric Cardiac Genomics Consortium (PCGC) (“internal controls”) [24]. To control for confounding from genetic ancestry, we selected 1832 cases and 5262 internal controls of European ancestry based on principle components analysis using PLINK version 1.9 [32], and 7509 non-Finnish, European (NFE) gnomAD subjects. Relatedness was checked using Peddy [32], and only unrelated cases were included in the association tests. To reduce batch effects in combined datasets from different sources [33], we limited the analysis in regions targeted by all xGen, NimbleGen, and MedExome, and with at least 10× coverage in 90% of samples. We then tested for similarity of the rare synonymous variant rate among cases and controls, a class that is mostly neutral with respect to disease status.

To identify PAH risk genes, we tested the burden of rare deleterious variants (AF ≤ 0.01%, LGD or D-mis) in each protein-coding gene in cases compared to controls. We used REVEL [30] scores to predict the deleteriousness of missense variants. To improve statistical power, we searched for a gene-specific REVEL score threshold that maximized the burden of rare deleterious variants in cases compared to controls, and use permutations to calculate statistical significance, similar to a published method [34] designed for variable threshold on allele frequency. Specifically, in each gene, we performed binomial tests with a given REVEL score threshold, ranging from 0.2 to 1 with 0.05 intervals, and defined the optimal threshold by the smallest *p* value (***P***_**0**_). Then, we performed 10,000,000 permutations (shuffling case-control labels); in each permutation, we obtained the smallest *p* value (***P*′**) using the same variable REVEL threshold procedure. We then recalibrated the *p* value for each gene as the fraction of permutations where ***P*′** ≤ ***P***_**0**_. We assigned a REVEL score of 1 (most deleterious) to LGD variants. In each binomial test, the null model is that the number of rare deleterious variants in cases follows a binomial distribution, given the total number of such variants in cases and controls, and a rate determined by fraction of cases in total number of subjects (cases and controls). We used *binom.test* function in R to calculate *p* values in the binomial tests. The script used for this variable threshold method is available from the following URL: https://github.com/ShenLab/VariableThresholdTest.

We expect that most genes will not be associated with PAH, and thus, the distribution of test statistics across most targeted regions in cases will not deviate from that of the controls. We checked for inflation using a quantile-quantile (Q-Q) plot and calculated the genomic control factor, lambda, using QQperm (https://cran.r-project.org/web/packages/QQperm/QQperm.pdf). Lambda equal to 1 indicates no deviation from the expected distribution.

To assess type I error and statistical power, we used *BMPR2* data from 1832 unrelated PAH Biobank cases and 5262 unrelated internal controls, all of European ancestry. In total, there were 188 rare (allele frequency < 10^−4^) LGD and D-mis variants. To assess whether type I error is controlled, we randomly shuffled case/control labels 10,000,000 times to generate 10,000,000 sets of data under the null and applied the variable threshold test to each dataset. We found the type I error rate was well controlled (see Additional file 2: Table S1).

*BMPR2* is a well-known PAH risk gene with large effect size and relatively long transcript, two conditions that lead to good statistical power in association tests. We expect that most of the undiscovered risk genes will have smaller effects or shorter transcripts. To estimate statistical power under realistic conditions with smaller effect sizes and shorter transcripts, we simulated data in two ways:

1. Randomly labeled cases and controls with a required fraction (*F*) of true cases being labeled as cases. *F* = 0.258 is equivalent to completely randomizing case and control labels, and therefore, it corresponds to the null model (relative risk = 1). *F* = 1 corresponds to original case/control data and maximizes the effect size (relative risk ~ 45). The power was estimated using two significance thresholds, α = 0.005 and α = 2.5e−6. In each setting, we ran 1000 simulations to calculate power. We compared our method (VT) with SKAT-O [35], a popular method for testing association of rare variants. VT has better power than SKAT-O with *F* in the range of 0.4–0.6, reflecting a range of modest effect sizes (relative risk ~ 2 to 5) as can be seen in Additional file 3: Figure S2A.
2. Given *F*, we then sampled a fraction of variants to generate a smaller dataset. This effectively creates datasets with smaller cumulative allele frequencies (CAF), a condition that fits for genes with shorter transcripts. We generated 1000 datasets under each condition (defined by *F* and CAF) to estimate power, setting the significance threshold at 2.5e−6. As shown in Additional file 3: Figure S2B, VT has better power than SKAT-O with all CAF values when *F* is between 0.45 and 0.6.

We defined the threshold for genome-wide significance by Bonferroni’s correction for multiple testing (*n* = 20,000 genes, threshold *p* value = 2.5e−6). We used the Benjamini-Hochberg procedure to estimate false discovery rate (FDR) by p.adjust in R. All *GGCX* and *KLK1* variants reported herein were confirmed by Sanger sequencing.

## Results

### Cohort characteristics

Characteristics of the PAH Biobank cohort are shown in Table 1 with more detailed characteristics shown in Additional file 4: Table S2. The cohort included 2572 cases: 43% IPAH, 48% APAH, 4% FPAH, and 5% other PAH. The APAH cases included 722 associated with autoimmune CTDs (mostly scleroderma with few cases of rheumatoid arthritis, systemic lupus erythematosus, and Sjogren’s syndrome), 268 with CHD, 139 with portopulmonary hypertension (PoPH), and 110 with other diseases (HHT, HIV, and rare disorders). The “other PAH” group included 110 drug- and toxin-induced (DTOX) PAH, 11 non-familial PVOD/PCH cases, and 1 persistent pulmonary hypertension of the newborn. The majority of cases (91.2%) were adult-onset with a cohort mean age of onset of 48 ± 19 years (mean ± SD). However, there was an enrichment of child-onset cases (95/268, 37.2%, *p* < 0.0001 by chi-square) in the APAH-CHD subclass. As has been reported previously for adult populations [36], there was an overall 3.7:1 ratio of females to males, with a 9:1 ratio for PAH associated with autoimmune disease and 1:1.2 ratio for the PoPH subclass. The genetic ancestries included European (72%), Hispanic (12%), African (11%), East Asian (2.7%), and South Asian (1.1%), fairly equally distributed among PAH subclasses. Within the APAH subclass, Africans were more likely to have disease associated with CTD (*p* = 0.02) and less likely with CHD (*p* = 0.0004) or PoPH (*p* = 0.001), consistent with a previous report [37]. There was an enrichment of PoPH among patients of Hispanic ancestry (*p* = 0.02).

**Table 1:** PAH Biobank cohort demographic and hemodynamic data

	All	IPAH	APAH	FPAH	Other*
Total (%)	2572	1110 (43.2)	1239 (48.2)	101 (3.9)	122 (4.7)
Age of onset, *n* (%)					
Child (dx age < 19)	226 (8.8)	94 (8.5)	112 (9.0)	15 (14.9)	5 (4.1)
Adult (dx age > =19)	2345 (91.2)	1015 (91.4)	1127 (91.0)	86 (85.1)	117 (95.9)
Mean age	48 ± 19	48 ± 18	49 ± 19	37 ± 15	47 ± 15
Gender, *n* (%)					
Female	2023 (78.7)	868 (78.2)	996 (80.4)	69 (68.3)	90 (73.8)
Male	549 (21.3)	242 (21.8)	243 (19.6)	32 (31.7)	32 (26.2)
Female to male ratio	3.7:1	3.6:1	4.1:1	2.2:1	2.8:1
Ancestry, *n* (%)					
European	1852 (72)	809 (73.0)	855 (69.0)	89 (88.1)	99 (81.2)
Hispanic	315 (12.3)	137 (12.3)	156 (12.6)	10 (9.9)	12 (9.8)
African	292 (11.4)	117 (10.5)	168 (13.6)	1 (1)	6 (4.9)
East Asian	70 (2.7)	25 (2.2)	41 (3.3)	0	4 (3.3)
South Asian	28 (1.1)	12 (1.1)	15 (1.2)	1 (1)	0
Others	15 (0.58)	10 (0.9)	4 (0.3)	0	1 (0.8)
Hemodynamic parameters					
MPAP (mmHg)	50 ± 14	52 ± 14	48 ± 14	58 ± 14	52 ± 13
MPCW (mmHg)	10 ± 4	10 ± 4	10 ± 4	10 ± 4	11 ± 4
CO, Fick (L/min)	4.5 ± 1.8	4.5 ± 1.7	4.6 ± 1.9	3.6 ± 1.0	4.2 ± 1.3
PVR (Woods units)	10.7 ± 7.0	11.2 ± 7.0	10.0 ± 7.1	14.9 ± 6.3	11.0 ± 6.6
MAP (mmHg)	90 ± 19	91 ± 20	90 ± 19	88 ± 16	94 ± 19
MAP:MPAP	1.9 ± 0.7	1.9 ± 0.7	2.0 ± 0.7	1.6 ± 0.5	1.9 ± 0.5

*Abbreviations*: *MPAP* mean pulmonary arterial pressure, *MPCW* mean pulmonary capillary wedge pressure, *CO* cardiac output by Frick’s method, *PVR* pulmonary vascular resistance, *MAP* mean arterial pressure

*Other included 110 diet- and toxin-induced PAH, 11 non-familial pulmonary veno-occlusive disease/pulmonary capillary hemangiomatosis, and 1 persistent pulmonary hypertension of the newborn

Hemodynamic data collected at the time of PAH diagnosis are also shown in Additional file 4: Table S2. Compared to IPAH cases, APAH-CTD cases had lower mean pulmonary artery pressure (MPAP) and lower pulmonary vascular resistance (PVR) by one-way ANOVA with correction for multiple comparisons. APAH-CHD and FPAH cases had higher MPAP and PVR compared to IPAH cases; FPAH cases also had decreased cardiac output (CO) as previously described. PoPH cases had increased CO and decreased PVR compared to IPAH cases. By these measures, APAH-CTD and PoPH cases had more moderate hemodynamic profiles compared to IPAH cases whereas both FPAH and APAH-CHD had less favorable profiles.

### Rare deleterious variants in established and recently reported PAH risk genes

**Fig. 1:**
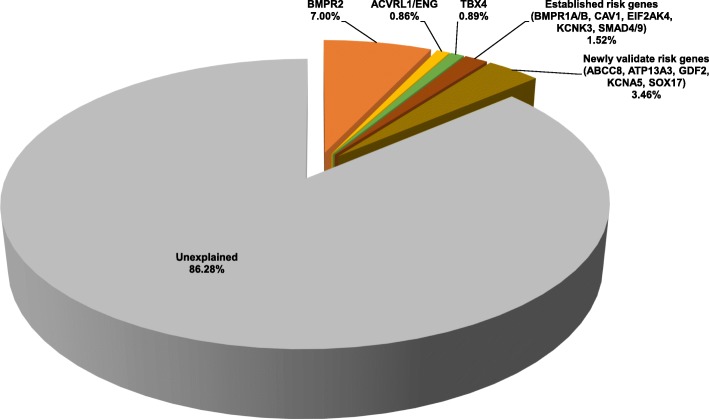
Contribution of known PAH risk genes in the PAH Biobank cohort (*n* = 2572 cases). *BMPR2*, *ACVRL1/ENG*, *TBX4*. Other established risk genes included in the analysis: *BMPR1A*, *BMPR1B*, *CAV1*, *EIF2AK4*, *KCNK3*, *SMAD4*, and *SMAD9*. Newly validated risk genes: *ABCC8*, *ATP13A1*, *GDF2*, *KCN5A*, *KLF2*, *SMAD1*, and *SOX17*

We screened for rare, predicted deleterious variants (allele frequency < 0.01% and likely gene damaging (LGD) or missense with REVEL score > 0.5 (D-Mis), see the “Methods” section) in 11 established PAH risk genes [38–41]: *ACVRL1*, *BMPR1A*, *BMPR1B*, *BMPR2*, *CAV1*, *EIF2AK4*, *ENG*, *KCNK3*, *SMAD4*, *SMAD9*, and *TBX4* by targeted capture/sequencing, multiple ligation-dependent probe amplification (MLPA) (to evaluate deletions/duplications in *BMPR2*, *ACVRL1*, and *ENG* only), and exome sequencing. We also screened the cohort for variants in 7 recently reported risk genes: *ABCC8*, *ATP13A3*, *GDF2/BMP9*, *KCNA5*, *KLF2*, *SMAD1*, and *SOX17*. Only 14% of cases (*n* = 349, 22% IPAH, 12% APAH, 55% FPAH, 11% other) carried rare predicted deleterious variants in these risk genes (see Fig. 1).

**Fig. 2:**
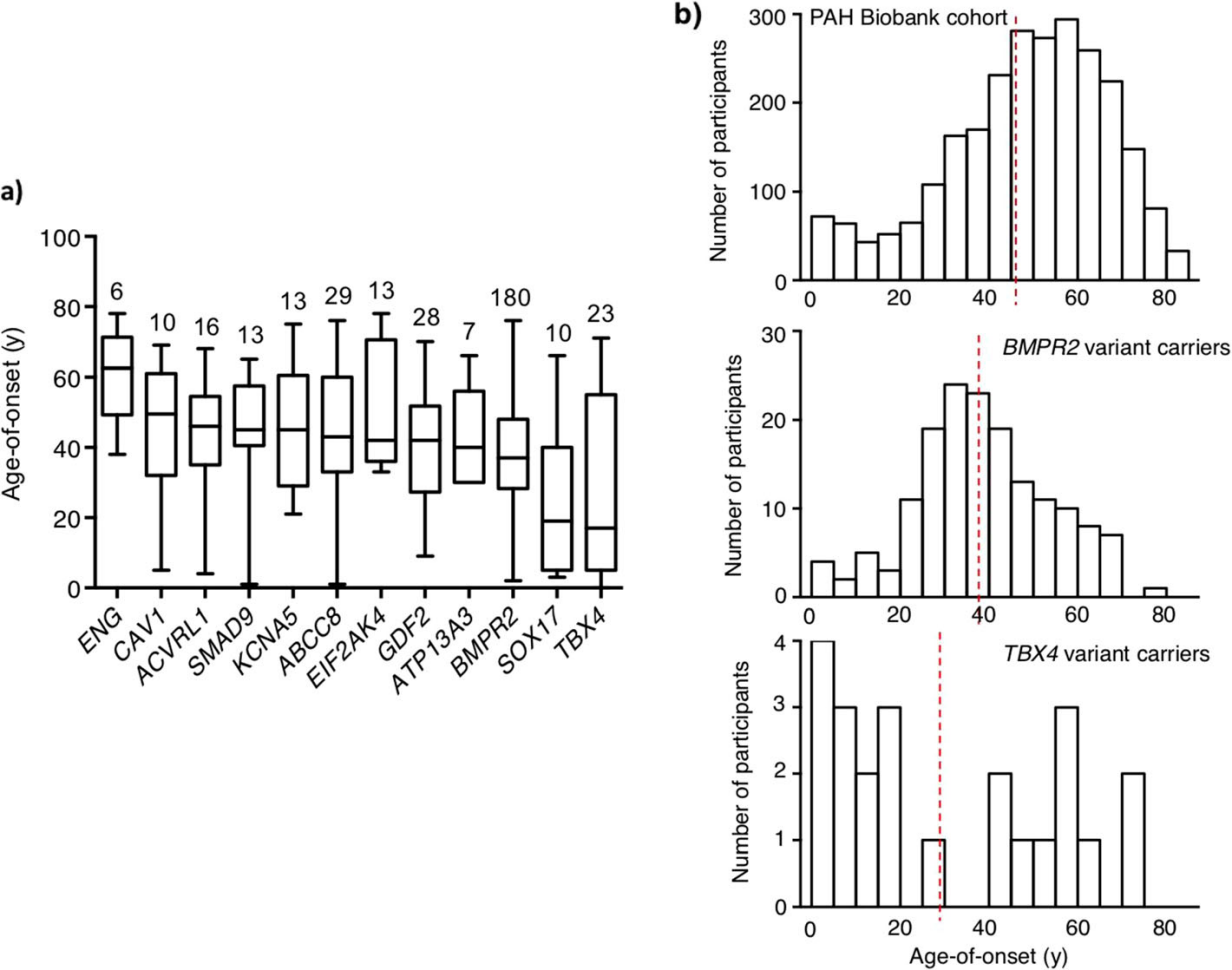
Age of disease onset for PAH Biobank cases with rare deleterious variants in known PAH risk genes. **a** Box plots showing median, interquartile range, and min/max values for age of disease onset (i.e., age at diagnostic right heart catheterization). The number of cases carrying variants for each gene is given above each box plot. Genes represented by less than four cases are not shown. **b** Histogram plots showing age-of-onset distributions for the whole cohort (*n* = 2572), *BMPR2* (*n* = 180), or *TBX4* (*n* = 23) variant carriers. Red vertical lines indicate the group means. *BMPR2* carriers had a younger mean age of onset (mean = 37 years, SD = 15; Mann-Whitney U test, *p* = 1.1E−15) but no enrichment of child-onset cases (binomial test *p* = 1, RR = 0.93) compared to the whole cohort, whereas *TBX4* carriers had a younger mean age of onset (mean = 29 years, SD = 25; Mann-Whitney U test, *p* = 0.001) and significant enrichment of child-onset cases (binomial test *p* = 6.5E−08, RR = 12.3) compared to the whole cohort

A complete list of cases carrying rare deleterious variants in established risk genes is provided in Additional file 5: Table S3. Not surprisingly, 68% of these cases carried variants in *BMPR2* (*n* = 119 variants in 180 cases: 9% exon deletions, 65% LGD, 26% D-Mis). The age of onset for *BMPR2* variant carriers was 38 ± 15 years (mean ± SD), significantly younger than that of the whole cohort (*p* = 1.1E−15, Mann-Whitney *U* test) but with a wide range of ages from 2 to 76 years (Fig. 2). The second most common genetic cause was *TBX4*, accounting for approximately 1% of cases (*n* = 23 cases with 22 variants: 12 LGD, 9 D-Mis, and 1 in-frame deletion), the majority of whom (57%) had a diagnosis of IPAH. Although more than 90% of cases in the PAH Biobank cohort had adult-onset disease, only 48% of the *TBX4* variant carriers had adult-onset disease. The overall mean age of onset was 29 ± 25 years (see Fig. 2a), with a bimodal distribution and a significant enrichment of pediatric-onset cases compared to the whole PAH cohort (*p* = 6.5E−08, RR = 12.3, binomial test) (see Fig. 2b), consistent with previous findings [14]. Deleterious variants in 9 additional genes were observed: *ACVRL1* (*n* = 16 cases, including 7 with HHT), *SMAD9* (13 cases), *CAV1* (10 cases), *ENG* (6 cases, including 2 with HHT), bi-allelic *EIF2AK4* (5 cases, including 2 with PVOD/PCH), *KCNK3* (3 cases), *BMPR1A* (4 cases), *SMAD4* (2 cases), and *BMPR1B* (2 cases). The three FPAH cases carrying the same LGD mutation in *CAV1* were related (proband, aunt, grandfather); only the proband was included in the downstream association analyses. We note that four cases (two IPAH, two FPAH) carried risk variants in *BMPR2* plus one other risk gene.

A complete list of rare deleterious variants in newly reported PAH risk genes is provided also in Additional file 5: Table S4. Nearly two thirds were variants in *ABCC8* (26 variants in 29 cases: all D-Mis) or *GDF2* (24 variants in 28 cases: 9 LGD, 15 D-Mis). The *ABCC8* variants occurred equally in IPAH and APAH cases (50:50) while the *GDF2* variants occurred primarily in IPAH cases (75%). Deleterious variants in the other new PAH risk genes were observed less frequently or not at all: *KCNA5* (*n* = 13 cases), *SOX17* (10 cases), *ATP13A3* (7 cases), *SMAD1* (2 cases), and *KLF2* (0 cases). The mean age of onset for these risk gene variant carriers ranged from 41 to 46 years, with the exception of *SOX17* which had a mean age of onset of 26 years (Fig. 2a), significantly younger than that of the whole cohort (*p* < 0.003, Mann-Whitney *U* test). The female to male ratio among these patients was 4.2:1, similar to that of the whole cohort. Overall, 71% of the variants in known risk genes were novel.

Locations of the risk gene variants are shown in Additional file 6: Figure S3 and Additional file 7: Figure S4. For *BMPR2*, all but 2 of the D-Mis variants are located within the first 500 amino acids of the protein, mostly within the conserved activin and protein kinase domains (Additional file 6: Figure S3). While the LGD variants are also clustered within the activin and protein kinase domains, 26 variants carried by 28 individuals are located downstream of these domains. For the other risk genes, the majority of D-Mis variants are also located in conserved protein domains (Additional file 7: Figure S4).

### Identification of novel PAH risk genes: *KLK1* and *GGCX*

Our extensive genome screening efforts failed to identify rare deleterious variants in known risk genes for 86% of the PAH Biobank cases. To identify novel PAH risk genes, we performed a gene-based, case-control association analysis. To prevent confounding by genetic ancestry, we included only participants of European ancestry (cases *n* = 1832; controls *n* = 7509 gnomAD WGS subjects and 5262 unaffected parents from the Pediatric Cardiac Genomics Consortium). To minimize technical batch effects of genotype data between cases and controls, we applied heuristic filters as described in the “Methods” section. We observed similar overall frequencies of rare synonymous variants in cases and controls (enrichment rate = 1.01, *p* value = 0.09), a class that is mostly neutral with respect to disease status (Additional file 8: Table S5). Further, a gene-level burden test confined to rare synonymous variants was consistent with a global null model (Additional file 9: Figure S5), indicating that technical batch effects would likely have minimal impact on genetic analyses.

**Fig. 3:**
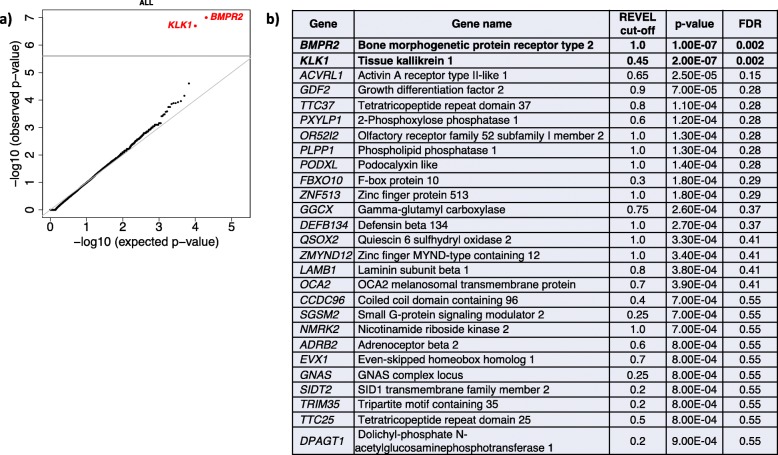
Gene-based association analysis using 1832 European cases from all PAH subclasses and 12,771 European controls. **a** Results of a binomial test confined to rare LGD and D-Mis (REVEL variable threshold) variants in 20,000 protein-coding genes. Horizontal gray line indicates the Bonferroni-corrected threshold for significance. **b** Complete list of top association genes (*p* ≤ 0.001)

We then proceeded to test for gene-specific enrichment of rare deleterious variants (allele frequency < 0.01%, LGD and D-Mis) in cases compared to controls. The use of in silico prediction tools to select deleterious missense variants can increase statistical power for rare variant association analyses [42], but the optimal threshold for deleteriousness scores is often gene specific [43]. To improve power, we implemented a rare variant burden test utilizing empirically determined, gene-specific deleterious score thresholds, a “variable threshold test.” The association results across all protein-coding genes, including 1832 European cases from all PAH subclasses, were generally consistent with the expectation under the null model (see Fig. 3). The Q-Q plot shows negligible genomic inflation, and we calculated the genomic control factor lambda = 1.02. Only two genes exceeded the Bonferroni-corrected threshold for significance: *BMPR2* (*p* = 1.0E−07, FDR = 0.002) and *KLK1* (*p* = 2.0E−07, FDR = 0.002) (Fig. 3). Established risk gene, *ACVRL1*, and recently reported *GDF2* fell just below the cutoff for significance. Variants in most other known risk genes are less frequent causes of PAH, and some have smaller effect size compared to *BMPR2*, requiring larger patient populations for genome-wide significance in association studies. *TBX4* and *SOX17* exhibited marginal association (*p* = 0.001 for each) which was not significant in this largely adult-onset cohort. *KLK1* encodes kallikrein 1, also known as tissue kallikrein, involved in the regulation of systemic blood pressure and vascular remodeling but not previously associated with pulmonary hypertension [44, 45].

**Fig. 4:**
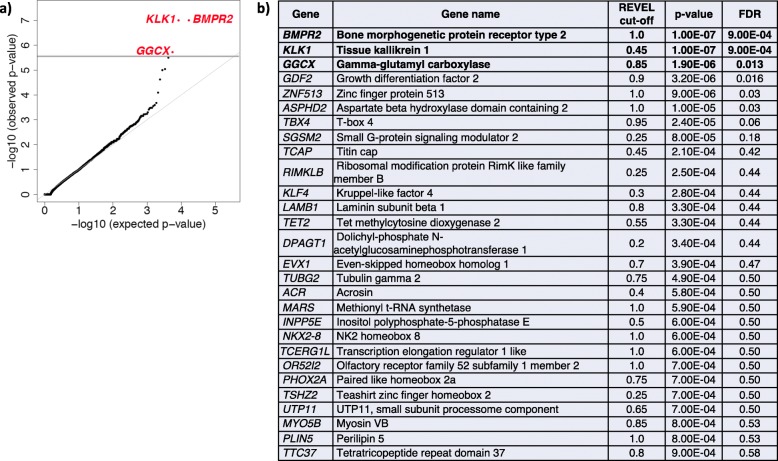
Gene-based association analysis using 812 European IPAH cases and 12,771 European controls. **a** Results of a binomial test confined to rare LGD and D-Mis (REVEL variable threshold) variants in 20,000 protein-coding genes. Horizontal gray line indicates the Bonferroni-corrected threshold for significance. **b** Complete list of top association genes (*p* ≤ 0.001)

We next repeated the analysis using 812 IPAH cases only (all European). Again, the Q-Q plot shows negligible inflation (see Fig. 4) with lambda = 0.98. For IPAH, we observed significant associations for *BMPR2* (*p* = 1.0E−7, FDR = 9.0E−04), *KLK1* (*p* = 1.0E−7, FDR = 9.0E−04), and *GGCX* (*p* = 5.0E−07, FDR = 0.002) (see Fig. 4). *GGCX* encodes gamma glutamyl carboxylase, implicated in coagulation factor deficiencies and ectopic mineralization of soft tissues [46]. These three genes were the only genes to reach genome-wide significance among IPAH cases. IPAH risk genes, *TBX4* and *GDF2*, fell just below the cutoff for significance. The near genome-wide associations of *ACVRL1* and *TBX4* are considered positive controls in our study as variants in *ACVRL1* are enriched in PVOD/PCH patients and variants in *TBX4* enriched in pediatric patients, both subgroups included in the cohort but at very low frequency. Likewise, while the association signal for *GDF2* fell below the cutoff (*p* = 3.0E−07, FDR = 0.002), we clearly provide confirmation of this new PAH risk gene. All association results for the total cohort or IPAH alone, with *p* ≤ 0.001, are listed in Figs. 3 and 4, respectively. Analysis of the depth of sequencing coverage of the targeted regions in *KLK1* and *GGCX* indicated that nearly 100% of samples attained read depths of at least 15×, excluding the possibility that the associations were driven by coverage differences between cases and controls (Additional file 10: Figure S6).

**Fig. 5:**
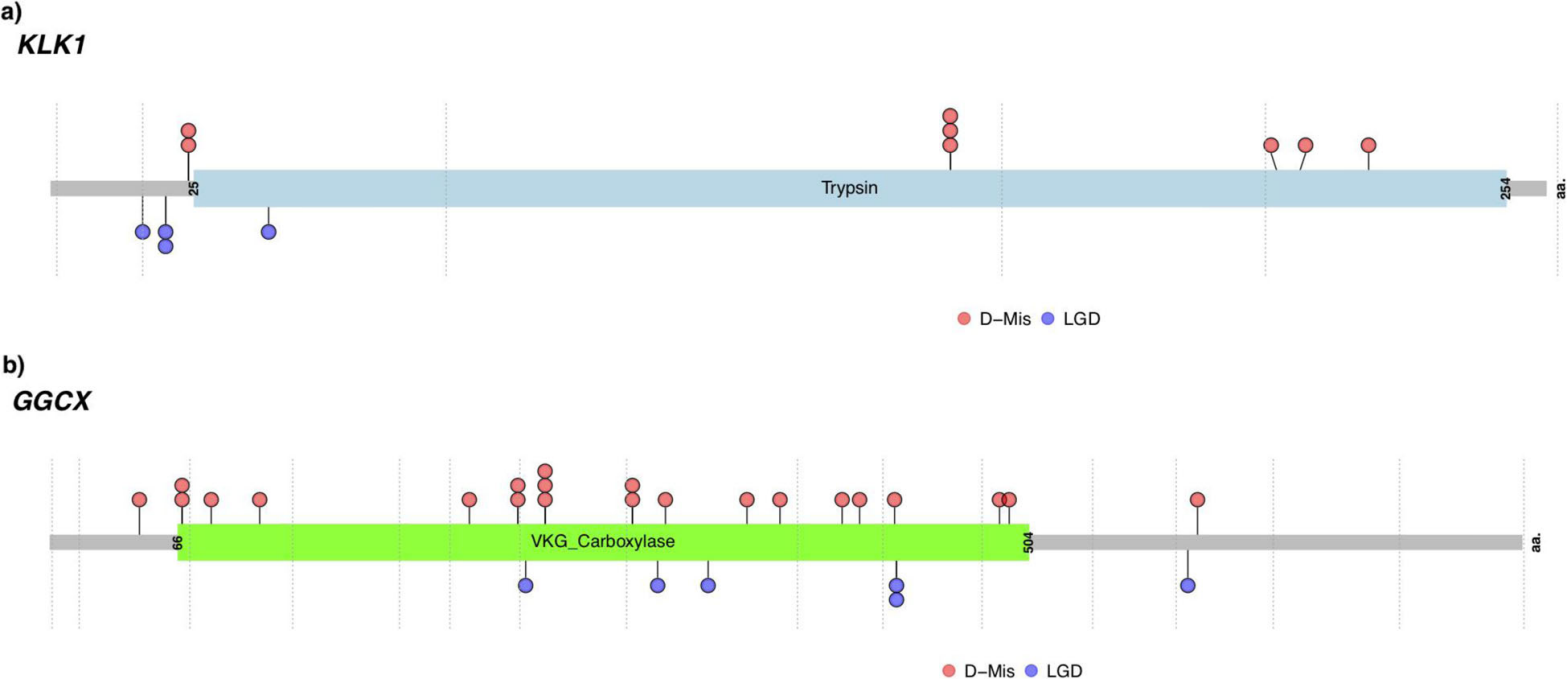
Locations of rare, predicted deleterious variants in *KLK1* (**a**) and *GGCX* (**b**) across the PAH Biobank cohort (*n* = 2572 cases). Locations are provided within the two-dimensional protein structures. The numbers of variants at each amino acid position are indicated along the *y*-axes. The vertical gray lines indicate exon borders. D-MIS, predicted damaging missense; LGD, likely gene disrupting (stop-gain, frameshift, splicing)

We next screened the entire PAH Biobank cohort, including participants of non-European ancestry, for rare deleterious variants in *KLK1* and *GGCX*. In total, 12 cases carried *KLK1* variants (10 IPAH, 2 APAH) and 28 cases carried *GGCX* variants (17 IPAH, 9 APAH, 1 FPAH, 1 unknown subclass) (Table 2). Most of the participants were of European ancestry; however, for *GGCX*, there were also 6 cases of African and 3 cases of Hispanic ancestries. The mean age of onset was similar to that of the overall cohort for both genes (*KLK1*, 49 ± 6; *GGCX*, 49 ± 3). The variants for *KLK1* included 4 LGD (1 stop-gain, 2 frameshifts, 1 splicing) and 8 D-Mis; variants for *GGCX* included 6 LGD (5 stop-gain, 1 frameshift), 21 D-Mis, and 1 in-frame deletion. Three *KLK1* (1 LGD, 2 D-Mis) and 5 *GGCX* (1 LGD, 4 D-Mis) variants were recurrent in the cohort. Locations of the variant amino acid residues are shown in Fig. 5. All but 1 of the *KLK1* and 2 of the *GGCX* missense variants, as well as the in-frame deletion, occur in conserved enzymatic domains.

**Table 2:** Rare, predicted deleterious *KLK1* and *GGCX* variants among 2572 PAH cases. Participants were heterozygous for the indicated variants

Participant ID	Gender	Age at dx (years)	PAH subclass	Ancestry	Gene**	Nucleotide change	Amino acid change	Variant type	MAF (ExAC)	CADD score	Revel score
08–022	F	60	IPAH	EUR	*KLK1*	c.46+1G>T	p.(=)	Splicing	–	24	–
10–096	F	68	IPAH	EUR	*KLK1*	c.60dup	p.Ile21Aspfs*12	Frameshift	4.29E−05	–	–
28–049	F	36	APAH-CHD	EUR	*KLK1*	c.60dup	p.Ile21Aspfs*12	Frameshift	4.29E−05	–	–
06–058	M	13	IPAH	EUR	*KLK1*	c.70C>T	p.Arg24Trp	D-Mis	8.47E−06	26	0.56
13–002	F	71	IPAH	EUR	*KLK1*	c.70C>T	p.Arg24Trp	D-Mis	8.47E−06	26	0.56
06–007	M	26	IPAH	EUR	*KLK1*	c.113G>A	p.Trp38*	Stop-gain	8.30E−06	35	–
12–061	F	51	IPAH	EUR	*KLK1*	c.469G>A	p.Gly157Ser	D-Mis	9.36E−05	29	0.72
14–018	M	61	IPAH	EUR	*KLK1*	c.469G>A	p.Gly157Ser	D-Mis	9.36E−05	29	0.72
17–075	F	82	APAH-CTD	EUR	*KLK1*	c.469G>A	p.Gly157Ser	D-Mis	9.36E−05	29	0.72
18–026	F	37	IPAH	EUR	*KLK1*	c.644G>A	p.Gly215Glu	D-Mis	–	30	0.85
19–013	F	51	IPAH	EUR	*KLK1*	c.650C>T	p.Pro217Leu	D-Mis	2.52E−05	29	0.60
19–033	F	37	IPAH	EUR	*KLK1*	c.689G>C	p.Trp230Ser	D-Mis	8.26E−06	25	0.50
06–014	M	35	FPAH	EUR	*GGCX*	c.137C>G	p.Ser46Cys	D-Mis	5.77E−05	23	0.70
04–020	F	36	IPAH	EUR	*GGCX*	c.G203G>C	p.Arg68Pro	D-Mis	–	35	0.96
12–207	F	43	IPAH	EUR	*GGCX*	c.G203G>C	p.Arg68Pro	D-Mis	–	35	0.96
32–003	M	81	IPAH	EUR	*GGCX*	c.248G>A	p.Arg83Gln	D-Mis	–	34	0.92
32–008	F	36	IPAH	AFR	*GGCX*	c.322C>T	p.Arg108Cys	D-Mis	1.65E−05	31	0.55
08–013	F	66	APAH-CTD	EUR	*GGCX*	c.646_647delinsCA	p.Val216Gln	In-frame	–	31	***
26–036	F	52	IPAH	EUR	*GGCX*	c.722 T>C	p.Phe241Ser	D-Mis	–	33	0.94
30–031	F	55	IPAH	EUR	*GGCX*	c.722 T>C	p.Phe241Ser	D-Mis	–	33	0.94
04–029	F	60	IPAH	EUR	*GGCX*	c.734 T>A	p.Leu245*	Stop-gain	–	40	–
04–087	F	54	IPAH	EUR	*GGCX*	c.763G>A	p.Val255Met	D-Mis	1.65E−05	34	0.86
34–005	M	66	IPAH	EUR	*GGCX*	c.763G>A	p.Val255Met	D-Mis	1.65E−05	34	0.86
11–004	F	24	IPAH	HIS	*GGCX*	c.763G>A	p.Val255Met	D-Mis	1.65E−05	34	0.86
22–108	F	40	APAH-HIV	AFR	*GGCX*	c.899C>T	p.Ser300Phe	D-Mis	2.53E−05	28	0.82
28–110	F	56	IPAH	AFR	*GGCX*	c.899C>T	p.Ser300Phe	D-Mis	2.53E−05	28	0.82
28–096	F	23	IPAH	EUR	*GGCX*	c.938_939del	p.Pro313Argfs*33	Frameshift	1.00E−04	–	–
08–046	F	53	APAH-Porto	EUR	*GGCX*	c.950G>A	p.Arg317Gln	D-Mis	1.67E−05	33	0.81
12–205	F	55	IPAH	EUR	*GGCX*	c.1017_1018insT	p.Ser340*	Stop-gain	–	–	–
15–008	F	14	APAH-CHD	EUR	*GGCX*	c.1075C>T	p.Arg359Cys	D-Mis	–	28	0.76
21–037	F	45	APAH-CTD	AFR	*GGCX*	c.1128C>G	p.Phe376Leu	D-Mis	–	27	0.85
06–039	F	28	IPAH	EUR	*GGCX*	c.1224C>A	p.His408Gln	D-Mis	8.24E−06	23	0.75
37–004	F	48	IPAH	EUR	*GGCX*	c.1249G>A	p.Asp417Asn	D-Mis	1.65E−05	26	0.72
30–034	F	49	APAH-CTD	HIS	*GGCX*	c.1304G>A	p.Arg435Gln	D-Mis	8.24E−06	29	0.67
14–029	M	48	IPAH	EUR	*GGCX*	c.1306C>T	p.Arg436*	Stop-gain	3.30E−05	41	–
37–010	F	77	APAH-CTD	EUR	*GGCX*	c.1306C>T	p.Arg436*	Stop-gain	3.30E−05	41	–
11–090	F	47	APAH	AFR	*GGCX*	c.1465G>A	p.Val489Met	D-Mis	2.47E−05	26	0.68
05–013	M	63	APAH-Porto	HIS	*GGCX*	c.1480 T>G	p.Ser494Ala	D-Mis	–	26	0.84
28–033	F	51	IPAH	EUR	*GGCX*	c.1758C>G	p.Tyr586*	Stop-gain	–	45	–
17–033	F	74	APAH-CTD	AFR	*GGCX*	c.1772C>T	p.Thr591Met	D-Mis	3.30E−05	29	0.83

*Rare, deleterious variants defined as gnomAD AF ≤ 1.00E−04 and REVEL > 0.5

**KLK1 transcript NM_002257.3 and *GGCX* transcript NM_000821.6

***REVEL score could not be computed for this 2-nt substitution because machine learning is based on 1-nt substitutions. Inclusion in the table was based on REVEL > 0.9 for single nt substitution and PROVEAN = deleterious for 2-nt substitution

*KLK1* belongs to a contiguous gene family cluster on chromosome 19 encoding 15 distinct peptidases. While some have highly restricted expression patterns (i.e., *KLK2* and *KLK3* in prostate), others are widely expressed [47]. Nine of the family members, including *KLK1*, are expressed in the lung and have been implicated in various lung diseases—inflammatory respiratory diseases, viral infections, and cancers [48]. We tested for enrichment of rare deleterious variants in the gene set expressed in the lung and observed a significant enrichment of LGD + D-Mis variants in European cases compared to controls (enrichment rate = 2.1, *p* = 0.004) (Additional file 11: Table S6A). We then performed gene-specific association analyses to determine which genes were contributing to the enrichment. The associations were stronger for IPAH than all PAH; while *KLK1* was the only gene to exceed the Bonferroni-corrected threshold for significance (OR = 13.9, *p* = 2.00E−07 for all PAH; OR = 26.2, *p* = 1.00E−07 for IPAH), five additional family members had an enrichment rate of rare deleterious variants greater than 2.0 for IPAH (Additional file 11: Table S6B).

### Clinical phenotypes of *KLK1* and *GGCX* variant carriers

Hemodynamic measurements at the time of PAH diagnosis for individual carriers of *KLK1* and *GGCX* variants are provided in Table 3. Clinical phenotypes of IPAH participants with *KLK1* or *GGCX* variants did not differ from that of other IPAH cases without variants in known risk genes (Additional file 12: Table S7). Overall, participants with predicted deleterious variants in either gene exhibited less severe clinical phenotypes compared to participants with variants in *BMPR2*. Carriers of both *KLK1* and *GGCX* variants were older at PAH onset and had decreased MPAP, increased CO, and decreased PVR compared to *BMPR2* carriers (Table 3). Furthermore, both *KLK1* and *GGCX* carriers had increased ratios of mean (systemic) arterial pressure to MPAP compared to *BMPR2* carriers (MAP:MPAP, Table 3).

**Table 3:** Clinical phenotypes of *KLK1* and *GGCX* variant carriers at PAH diagnosis and compared to mean phenotypes of *BMPR2* variant carriers

Participant ID	Gender	PAH subclass	Gene	Age at dx (years)	MPAP (mmHg)	MPCW (mmHg)	CO, Fick (L/min)	PVR (Woods units)	MAP (mmHg)	MAP:MPAP
08–022	F	IPAH	*KLK1*	60	55	7	2.6	18.46	98	1.78
10–096	F	IPAH	*KLK1*	68	46	9	3.95	9.37	NA	NA
28–049	F	APAH-CHD	*KLK1*	36	61	5	3.77	14.85	82	1.34
06–058	M	IPAH	*KLK1*	13	38	8	8.2	3.66	92	2.42
13–002	F	IPAH	*KLK1*	71	44	9	3.7	9.46	NA	NA
06–007	M	IPAH	*KLK1*	26	41	11	5.8	5.17	97	2.37
12–061	F	IPAH	*KLK1*	51	53	13	NA	NA	106	2.00
14–018	M	IPAH	*KLK1*	61	37	6	4.38	7.08	98	2.65
17–075	F	APAH-CTD	*KLK1*	82	27	7	5.23	3.82	NA	NA
18–026	F	IPAH	*KLK1*	37	73	15	NA	NA	NA	NA
19–013	F	IPAH	*KLK1*	51	42	13	3.6	8.06	112	2.67
19–033	F	IPAH	*KLK1*	37	34	8	5.53	4.70	93	2.74
Mean ± SD, *KLK1*				49 ± 20	46 ± 13	9 ± 3	4.7 ± 1.6	8.5 ± 4.9	97 ± 9	2.3 ± 0.5
*n*, *KLK1*				12	12	12	10	10	8	8
Mean ± SD, *BMPR2*				38 ± 15	59 ± 12	10 ± 4	3.7 ± 1.3	15.3 ± 7.3	90 ± 17	1.6 ± 0.4
*n*, *BMPR2*				181	175	172	123	120	114	114
*p* value, *KLK1* vs *BMPR2*				0.014	0.0007	NS	0.02	0.004	NS	< 0.0001
06–014	M	FPAH	*GGCX*	35	56	7	2.2	22.27	103	1.84
04–020	F	IPAH	*GGCX*	36	78	8	2.6	26.92	71	0.91
12–207	F	IPAH	*GGCX*	43	68	7	2.63	23.19	NA	NA
32–003	M	IPAH	*GGCX*	81	31	4	4.51	5.99	NA	NA
32–008	F	IPAH	*GGCX*	36	49	15	6.13	5.55	87	1.78
08–013	F	APAH-CTD	*GGCX*	66	40	9	5.8	5.34	120	3.00
26–036	F	IPAH	*GGCX*	52	70	9	NA	NA	86	1.23
30–031	F	IPAH	*GGCX*	55	56	15	NA	NA	121	2.16
04–029	F	IPAH	*GGCX*	60	51	8	5.53	7.78	NA	NA
04–087	F	IPAH	*GGCX*	54	40	14	5.64	4.61	115	2.88
34–005	M	IPAH	*GGCX*	66	43	14	6.85	4.23	83	1.93
11–004	F	IPAH	*GGCX*	24	77	11	NA	NA	NA	NA
22–108	F	APAH-HIV	*GGCX*	40	78	13	5.7	11.40	94	1.21
28–110	F	IPAH	*GGCX*	56	78	NA	3.6	NA	85	1.09
28–096	F	IPAH	*GGCX*	23	65	7	4.8	12.08	NA	NA
08–046	F	APAH-Porto	*GGCX*	53	44	10	6.23	5.46	88	2.00
12–205	F	IPAH	*GGCX*	55	52	8	NA	NA	NA	NA
15–008	F	APAH-CHD	*GGCX*	14	60	12	2.5	19.20	62	1.03
21–037	F	APAH-CTD	*GGCX*	45	31	12	5.26	3.61	NA	NA
06–039	F	IPAH	*GGCX*	28	48	11	4.6	8.04	81	1.69
37–004	F	IPAH	*GGCX*	48	49	18	3.6	8.61	67	1.37
30–034	F	APAH-CTD	*GGCX*	49	38	17	NA	NA	NA	NA
14–029	M	IPAH	*GGCX*	48	50	14	NA	NA	NA	NA
37–010	F	APAH-CTD	*GGCX*	77	28	6	NA	NA	74	2.64
11–090	F	APAH	*GGCX*	47	NA	NA	NA	NA	NA	NA
05–013	M	APAH-Porto	*GGCX*	63	33	6	6.31	4.28	119	3.61
28–033	F	IPAH	*GGCX*	51	61	NA	3.6	NA	85	1.39
17–033	F	APAH-CTD	*GGCX*	74	45	7	3.69	10.30	88	1.96
Mean ± SD, *GGCX*				49 ± 16	53 ± 15	10 ± 4	4.6 ± 1.4	10.5 ± 7.4	91 ± 18	1.9 ± 0.8
*n*, *GGCX*				28	27	25	20	18	18	18
Mean ± SD, *BMPR2*				38 ± 15	59 ± 12	10 ± 4	3.7 ± 1.3	15.3 ± 7.3	90 ± 17	1.6 ± 0.4
*n*, *BMPR2*				181	175	172	123	120	114	114
*p* value, *GGCX* vs *BMPR2*				< 0.0001	0.02	NS	0.004	0.01	NS	0.007

*Abbreviations*: *Age at dx* participant age at diagnosis/right heart catheterization, *MPAP* mean pulmonary arterial pressure, *MPCW* mean pulmonary capillary pressure, *CO* cardiac output by the Frick method, *PVR* pulmonary vascular resistance, *MAP* mean arterial pressure

A known *KLK1* single nucleotide polymorphism conferring at least partial loss of function occurs with high frequency in the general population [49, 50]. The c.230G>A; p.R77H SNP (formerly called c.230G>A; p.R53H) has been associated with decreased urinary kallikrein activity and aberrant flow-mediated arterial remodeling but not systemic hypertension [49, 50]. We screened the PAH Biobank cohort for the c.230G>A; p.R77H SNP and compared the cohort allele frequency with the frequency observed in gnomAD. No enrichment was observed in the PAH Biobank cohort (Additional file 13: Table S8), and none of the carriers of rare deleterious *KLK1* variants also carried the c.230G>A; p.R77H SNP. Thus, the observed association of rare, deleterious *KLK1* variants with PAH and associated phenotypes could not be explained by coincident occurrence of the common SNP.

## Discussion

Using exome sequencing of a large PAH Biobank cohort recruited by 28 participating centers, followed by rare deleterious variant identification and gene-based association analysis, we identified *KLK1* and *GGCX* as novel candidate genes for PAH. These candidate risk genes suggest new pathogenic mechanisms outside of the TGF-β/BMPR2 signaling pathway. We showed that carriers of rare, predicted deleterious variants in *KLK1* or *GGCX* have less severe clinical phenotypes compared to carriers of *BMPR2* variants. In addition, we identified 252 novel rare deleterious variants in 17 known PAH risk genes and confirmed the importance of *TBX4* and *SOX17* in early-onset disease as well as the association of *GDF2* with IPAH.

Our study complements the recently reported findings from the UK NIHR BioResource–Rare Diseases PAH Study with some similarities and some differences. Our cohort differed from the UK cohort in size and composition. The PAH Biobank is more than twice as large as the UK cohort and includes PAH associated with other diseases (APAH), a subgroup that has not been widely studied and was not included in the UK cohort. In agreement with the findings from the UK cohort [23], we provide confirmation of *GDF2* among 1832 all PAH and 812 IPAH cases of European ancestry. In total, we identified 24 variants, only 2 of which had been reported previously. *GDF2* encodes a well-characterized ligand for *BMPR2*, and these data further confirm an important role for *GDF2* in IPAH, as well as other PAH subclasses. Similar to the UK cohort, as well as our previous report of a cohort enriched in APAH-CHD cases [22], we observed a low frequency of *SOX17* variants (0.4%) in the PAH Biobank. We reported enrichment of *SOX17* variants in APAH-CHD; the low frequency of *SOX17* variants in the PAH Biobank and UK cohort is likely due, at least in part, to the paucity of APAH-CHD cases in both cohorts. Interestingly, a genome-wide association study of common SNPs involving both the PAH Biobank and the UK cohort identified SNPs in a putative endothelial-acting enhancer region of *SOX17* in PAH [51], suggesting that common variants may play an important role in susceptibility to PAH.

Differences in the two studies of rare variants included lack of genome-wide association of *ATP13A3* or *AQP1* in the PAH Biobank and no significant association of *KLK1* or *GGCX* in the UK cohort. We screened for *ATP13A1* rare deleterious variants and identified only seven cases with variants. *AQP1* not only failed to reach genome-wide significance but also was not among the expanded list of genes with *p* ≤ 0.001 for either the whole PAH cohort or the IPAH alone. Based on the small relative risks and associated confidence intervals from the UK cohort (RR = 0.37, CI 0.06–1.47 for *ATP13A3*; RR = 0.19, CI 0.004–1.53 for *AQP1*), it was not unexpected that that by chance we would observe no association with these genes and PAH. In terms of *KLK1* and *GGCX*, if the effect sizes for the 2 genes are equal to the estimates from the US cohort (relative risk ~ 12 and 4, respectively), we would expect only 6 and 9 carriers in the UK cohort. As a result, the UK cohort would have poor power (~ 30% and 10%, respectively) to detect these two genes with genome-wide significance.

The new PAH candidate risk genes identified in the current study, *KLK1* and *GGCX*, are both expressed in the lung and vascular tissues and play important roles in vascular hemodynamics and inflammation, but have not been implicated in PAH previously. *KLK1*, also known as tissue kallikrein 1, is a major component of the kallikrein-kinin system that, together with the renin-angiotensin system, regulates blood pressure and cardiovascular function. In rodent and in vitro studies, *KLK1* is constitutively expressed by endothelial cells, and endothelial activation leads to release of active protease, matrix degradation, smooth muscle cell migration, and vascular sprouting [44], processes relevant to PAH. *KLK*^*−/−*^ mice exhibit impaired neovascularization, and adenoviral overexpression of human *KLK1* promotes neovascularization in *KLK*^*−/−*^ mice, rat mesentery arteries, and zebrafish [52]. *KLK1* is part of a highly conserved, serine protease subfamily. We observed enrichment of rare deleterious variants in a gene set of nine *KLK* genes expressed in the lung, suggesting candidate genes for further investigation including *KLK12* which may play a role in angiogenesis via indirect regulation of vascular endothelial growth factor [53]. Together, the data suggest that both deficiency and loss of function mutations in *KLK1*, and potentially other *KLK* genes, may cause impaired neovascularization of injured distal arterioles in PAH. Gene delivery of tissue *KLK1* via adenoviral vectors, protein infusion, or genetically modified stem cells has shown beneficial effects in multiple models of vascular diseases [54]. There may be potential for gene delivery of *KLKs* as a treatment for PAH.

*GGCX* encodes gamma glutamyl carboxylase, responsible for the post-translational modification of vitamin K-dependent proteins involved in coagulation, soft tissue mineralization, prevention of vascular calcification, inflammation, bone formation, and cell proliferation [46]. It is unclear what all the targets of GGCX are, but mutations in GGCX could alter inflammatory responses or cell proliferation of pulmonary artery smooth muscle and/or endothelial cells in pulmonary arterioles, both hallmarks of PAH. Homozygous mutations in *GGCX* cause vitamin K-dependent clotting factor deficiency (MIM #277450) as well as pseudoxanthoma elasticum (MIM #264800), an ectopic mineralization disorder. None of the PAH Biobank *GGCX* heterozygous variant carriers had diagnoses of bleeding disorders or pseudoxanthoma elasticum. *Ggcx*^*−/−*^ mice die pre- or perinatally due to massive bleeding, but heterozygotes are viable [55]. Thus, the heterozygous knockout mouse may provide a model for testing the effect of *Ggcx* on PAH phenotypes.

The differences in etiology, clinical course, and prognosis for child- vs adult-onset PAH are an area of active investigation. Previous studies have implicated *BMPR2*, *TBX4*, and *SOX17* in child-onset disease. In our large PAH Biobank cohort, *BMPR2* variant carriers exhibited a shift towards younger age of onset, but the overall age distribution was similar to that of the whole cohort. *TBX4* exhibited a bimodal distribution with significant enrichment of variants among pediatric-onset cases. Consistent with our previous report of *SOX17* in APAH-CHD, *SOX17* carriers in the PAH Biobank also had a relatively young mean age of onset (26 years). The hypothesis that pediatric PAH is linked to lung growth and development [56] is consistent with roles for *TBX4* and *SOX17*, prominent developmental transcription factors [57], in early-onset disease.

## Conclusions

In summary, we have identified *KLK1* and *GGCX* as new candidate risk genes for PAH, accounting for ~ 0.4% and 0.9% of PAH Biobank cases, respectively. The total percentage of cases with rare deleterious variants in known and novel genes combined was 15.1% (389/2572). The large proportion of unexplained cases can be accounted for by incomplete penetrance which requires a larger sample size, analyses being confined to protein-coding sequences, and genetic heterogeneity, as well as environmental/non-genetic factors. Incomplete penetrance is the rule in PAH with the major susceptibility gene, *BMPR2*, exhibiting only 20–40% penetrance. The finding of risk gene carriers across multiple PAH subclasses is not necessarily surprising. As APAH and other rare subclasses have not been studied extensively, there was no a priori hypothesis as to whether the risk variants would be observed in the same or different genes compared to IPAH/FPAH. Since there is clear overlap in pulmonary phenotypes between the subclasses, it is likely that there will be both overlapping and distinct risk genes. The growing list of PAH risk genes and variants indicates that exome sequencing may be useful in families with PAH if no genetic cause is identified with panel gene testing. However, genomic studies of larger international consortia will be necessary to better clinically characterize these rare genetic subtypes of PAH.

## Supplementary information

**Additional file 1: Figure S1.** Depth of coverage for all samples across all targeted regions.

**Additional file 2: Table S1.** Type I error rates at four different significance thresholds.

**Additional file 3: Figure S2**. A) Power as a function of effect size: comparison of the variable threshold (VT) approach used in our study and SKAT-O; Figure S2B) Power as a function of cumulative allele frequency (CAF): comparison of the variable threshold (VT) approach used in our study and SKAT-O.

**Additional file 4: Table S2.** PAH Biobank cohort demographic data by PAH subclass.

**Additional file 5: Table S3.** Rare, predicted deleterious variants in established PAH risk genes among 2572 PAH cases; **Table S4.** Rare, predicted deleterious variants in recently reported PAH risk genes among 2572 PAH cases.

**Additional file 6: Figure S3.** Locations of rare deleterious PAH patient-derived *BMPR2* variants within the two-dimensional protein structure.

**Additional file 7: Figure S4.** Locations of rare deleterious PAH patient-derived other previously reported PAH risk gene variants within the two-dimensional protein structures.

**Additional file 8: Table S5.** Similar frequency of rare synonymous variants among European PAH cases and non-Finnish European gnomAD and in-house controls.

**Additional file 9: Figure S5.** Gene-level burden test for rare synonymous variants using 1832 European cases and 12,771 European controls.

**Additional file 10: Figure S6.** Depth of coding sequence coverage for *GGCX* and *KLK1*.

**Additional file 11: Table S6**A. Enrichment of rare deleterious variants in a *KLK* gene-set expressed in lung among 1832 European PAH cases and 12,771 European controls; **Table S6**B. Association analysis of *KLK* genes expressed in lung using 1832 (all PAH) or 812 (IPAH) European cases and 12,771 European controls.

**Additional file 12: Table S7.** Mean clinical phenotypes of *KLK1* and *GGCX* IPAH cases compared to other IPAH cases without variants in known risk genes.

**Additional file 13: Table S8.** Lack of enrichment of *KLK1* common SNP, R77H, in the PAH Biobank cohort compared to gnomAD population data.

## Data Availability

The datasets used and/or analyzed during the current study are available via contact with the corresponding author whose Confidentiality Agreement with the Regeneron Genetics Center grants to him a non-exclusive, worldwide, irrevocable, perpetual, royalty-free sublicensable license to access and use the genomic data for any and all purposes. Therefore, while the data are not uploaded to a publicly available database, direct access to the data are granted by the corresponding author on reasonable request who has full administrative access to all of the data. A subset including 183 affected participants were included in previous publications from our group [14, 20, 22]. The script used for the variable threshold method is available from the following URL: https://github.com/ShenLab/VariableThresholdTest.
