# Correction to: Novel risk genes and mechanisms implicated by exome sequencing of 2572 individuals with pulmonary arterial hypertension

**DOI:** 10.1186/s13073-022-01014-0

**Published:** 2022-02-07

**Authors:** Na Zhu, Michael W. Pauciulo, Carrie L. Welch, Katie A. Lutz, Anna W. Coleman, Claudia Gonzaga-Jauregui, Jiayao Wang, Joseph M. Grimes, Lisa J. Martin, Hua He, PAH Biobank Enrolling Centers’ Investigators, Yufeng Shen, Wendy K. Chung, William C. Nichols

**Affiliations:** 1 Department of Pediatrics, Columbia University Medical Center, New York, NY USA; 2 Department of Systems Biology, Columbia University, New York, NY USA; 3 Division of Human Genetics, Cincinnati Children’s Hospital Medical Center, 3333 Burnet Avenue MLC, Cincinnati, OH 7016 USA; 4 Department of Pediatrics, College of Medicine, University of Cincinnati, Cincinnati, OH USA; 5 Regeneron Genetics Center, Regeneron Pharmaceuticals, Tarrytown, New York, NY USA; 6 Department of Biomedical Informatics, Columbia University, New York, NY USA; 7 Herbert Irving Comprehensive Cancer Center, Columbia University Medical Center, New York, NY USA; 8 Department of Medicine, Columbia University Medical Center, New York, NY USA

**Correction to: Genome Med 11, 69 (2019)**

**https://doi.org/10.1186/s13073-019-0685-z**

Following publication of the original article [1], two errors were identified in the Results section.

1. The sentence currently reads:

“For IPAH, we observed significant associations for BMPR2 (*p* = 1.0E − 7, FDR = 9.0E− 04), KLK1 (*p* = 1.0E− 7, FDR = 9.0E− 04), and GGCX (*p* = 5.0E− 07, FDR = 0.002) (see Fig. 4).

The sentence should read

“For IPAH, we observed significant associations for BMPR2 (*p* = 1.0E − 7, FDR = 9.0E− 04), KLK1 (*p* = 1.0E− 7, FDR = 9.0E− 04), and GGCX (*p* = 1.9E-06, FDR = 0.013).

Further down in the text:

2. The sentence currently reads:

“Likewise, while the association signal for GDF2 fell below the cutoff (*p* = 3.0E−07, FDR = 0.002), we clearly provide confirmation of this new PAH risk gene”.

The sentence should read:

“Likewise, while the association signal for GDF2 fell below the cutoff (*p* = 3.2E-06, FDR = 0.016), we clearly provide confirmation of this new PAH risk gene”.

The original article [1] has been corrected.
