# Supplementary figures and images for: Novel risk genes and mechanisms implicated by exome sequencing of 2572 individuals with pulmonary arterial hypertension
Source: Genome Med. 2019 Nov 14;11:69. doi: 10.1186/s13073-019-0685-z (PMC6857288; doi:10.1186/s13073-019-0685-z)

Figure S1. Depth of coverage plot indicates that 98.3% of samples have D15 for 90% of targeted regions.

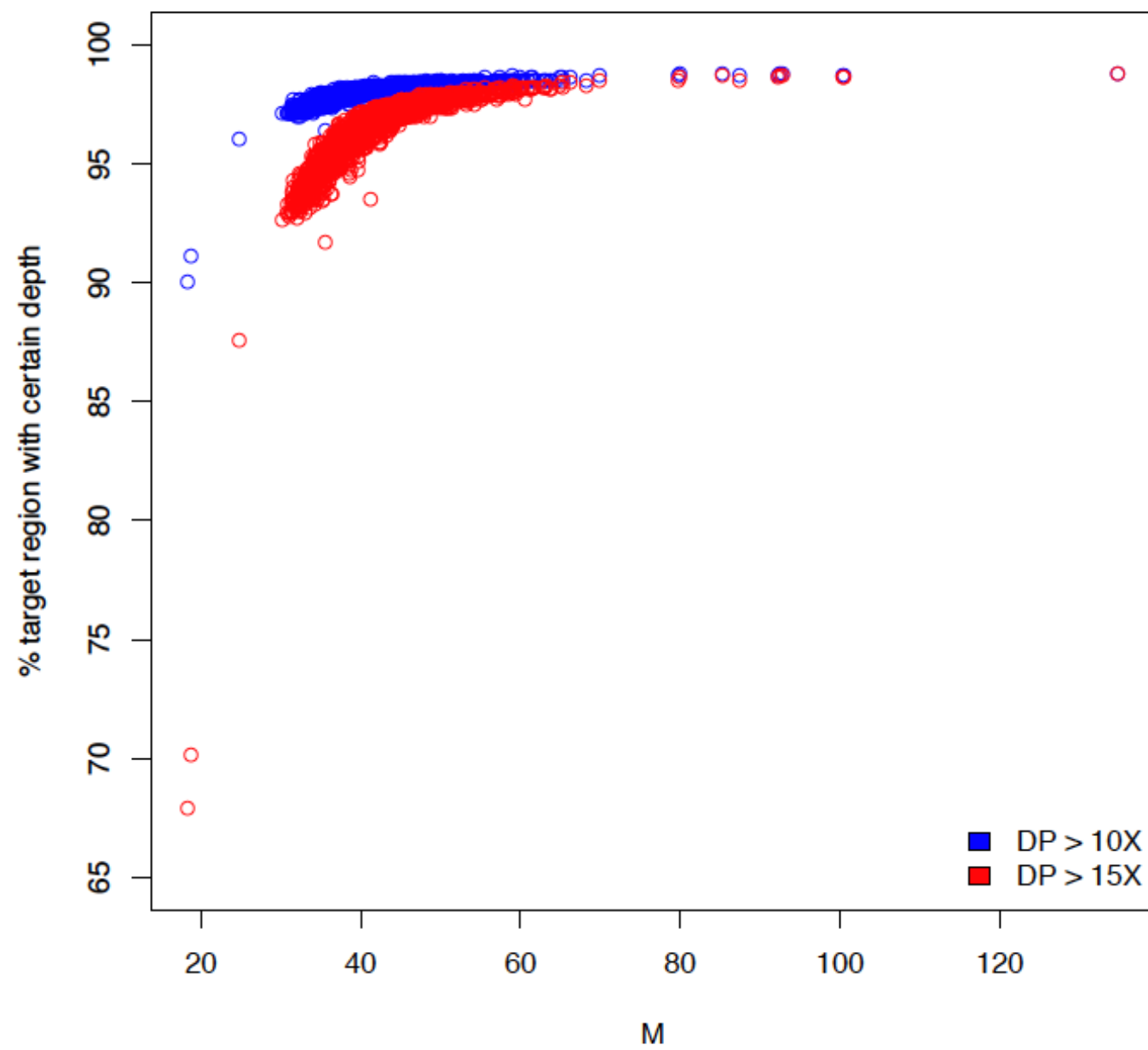

Supplement: Supplementary file 1 — Additional file 1: Figure S1. Depth of coverage for all samples across all targeted regions. [file 13073_2019_685_MOESM1_ESM.pdf]
