# Supplementary material for: Novel risk genes and mechanisms implicated by exome sequencing of 2572 individuals with pulmonary arterial hypertension
Source: Genome Med. 2019 Nov 14;11:69. doi: 10.1186/s13073-019-0685-z (PMC6857288; doi:10.1186/s13073-019-0685-z)
Supplement: Supplementary file 2 — Additional file 2: Table S1. Type I error rates at four different significance thresholds. [file 13073_2019_685_MOESM2_ESM.docx]

**Table S1. Type I error rates at four different significance thresholds.** The dataset included 188 rare *BMPR2* variants (AF <10-4 and variant type likely gene damaging or missense) among 1832 unrelated European PAH Biobank cases and 5,262 unrelated European internal controls. 10,000,000 simulations were performed under the null model (random labeling of cases and controls with case:control =1832:5262 for each simulation).

| **Significance threshold (α)** | **Type I error rate** |
| --- | --- |
| 0.001 | 9.77E-04 |
| 1.00E-04 | 9.76E-05 |
| 1.00E-05 | 9.72E-06 |
| 2.50E-06 | 2.16E-06 |
