# Supplementary material for: Novel risk genes and mechanisms implicated by exome sequencing of 2572 individuals with pulmonary arterial hypertension
Source: Genome Med. 2019 Nov 14;11:69. doi: 10.1186/s13073-019-0685-z (PMC6857288; doi:10.1186/s13073-019-0685-z)
Supplement: Supplementary file 3 — Additional file 3: Figure S2. A) Power as a function of effect size: comparison of the variable threshold (VT) approach used in our study and SKAT-O; Figure S2B) Power as a function of cumulative allele frequency (CAF): comparison of the variable threshold (VT) approach used in our study and SKAT-O. [file 13073_2019_685_MOESM3_ESM.pdf]

Figure S2. A) Power as a function of effect size: comparison of the variable threshold (VT) approach used in our study and SKAT-O. We used *BMPR2* data from 1832 unrelated European PAH Biobank cases and 5,262 unrelated European internal controls for the analysis. In total, there are 188 rare variants (AF <10-4 and variant type likely gene damaging or missense). Under each condition, we randomly label cases and controls with a required fraction (*F*) of true cases being labeled as cases. Since the effect size (relative risk) of *BMPR2* is fixed in the original data, *F* determines the effect size in each condition. F= 0.258 is equivalent to completely randomizing cases and controls labels and therefore, it corresponds to the null model (relative risk=1). F=1 corresponds to original case/control data and maximizes the effect size (relative risk ~45). The power was estimated using two significance thresholds, α=0.005 and α=2.5E-06. 1000 simulations were run in each setting. Better power can be observed for VT compared to SKAT-O across a fraction range of 0.4-0.6, reflecting a range of modest effect sizes (relative risk ~ 2 to 5).

B) Power as a function of cumulative allele frequency (CAF): comparison of the variable threshold (VT) approach used in our study and SKAT-O. In this comparison, we aim to show the power for genes with different size. Given a sample size and effect size, cumulative allele frequency is determined largely by gene transcript size. We used the same *BMPR2* data set as Figure 1. Given an effect size (fixed by fraction of true cases / simulated cases, as the x-axis in figure 1), we randomly sample a fraction of genotypes from the original data to reach smaller CAF than *BMPR2*, and performed 1000 simulations for power analysis. The power was estimated using a single significance threshold (α=2.5E-06). The larger the CAF, the greater the power for each effect size. We observed better power of VT compared to SKAT-O under all conditions. Similar results were obtained using a threshold of α=0.005 (data not shown).

A.

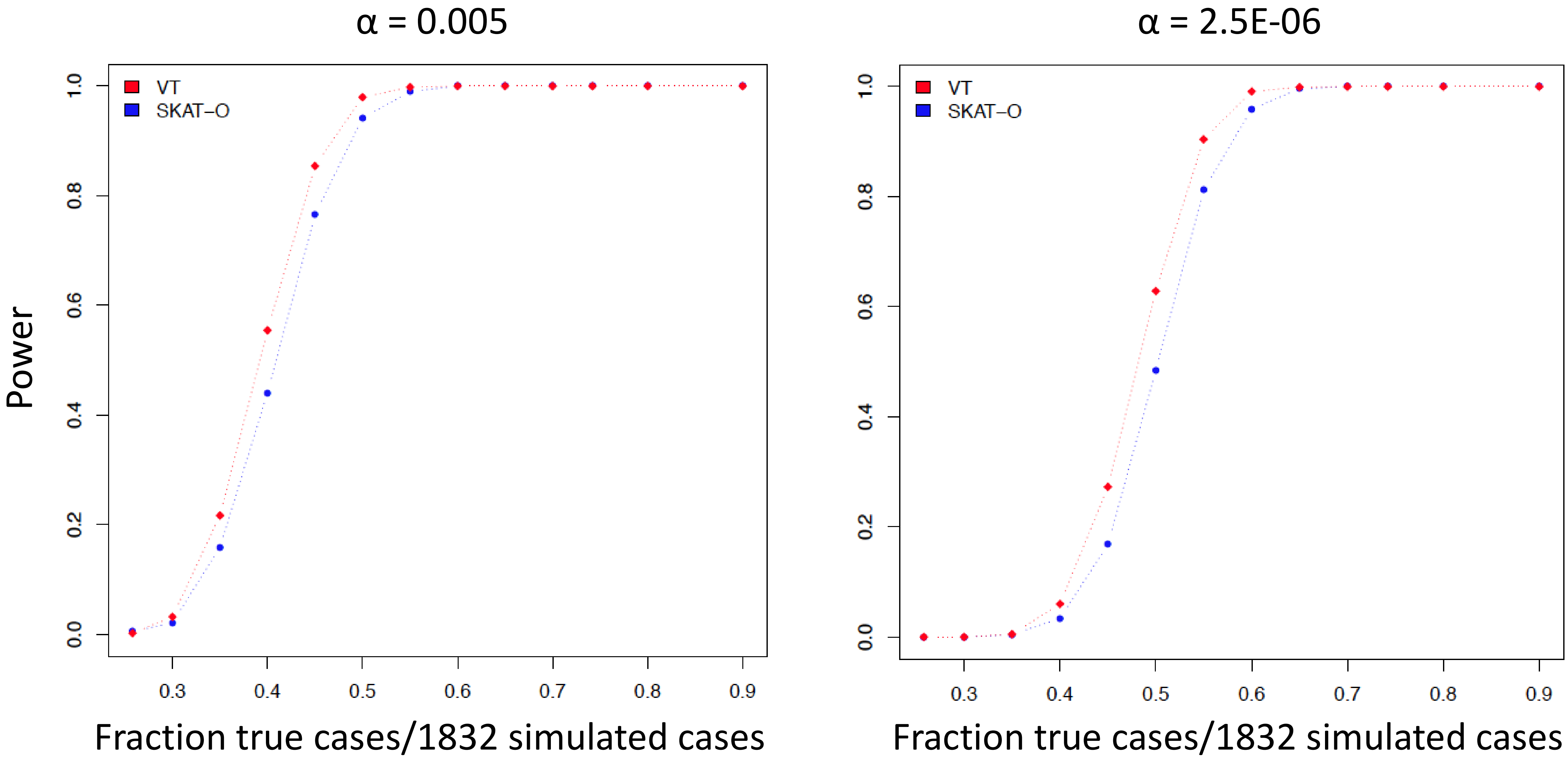
α = 0.005
α = 2.5E-06
Power
VT
SKAT–O
0.0
0.2
0.4
0.6
0.8
1.0
0.3
0.4
0.5
0.6
0.7
0.8
0.9
Fraction true cases/1832 simulated cases
Fraction true cases/1832 simulated cases

B.

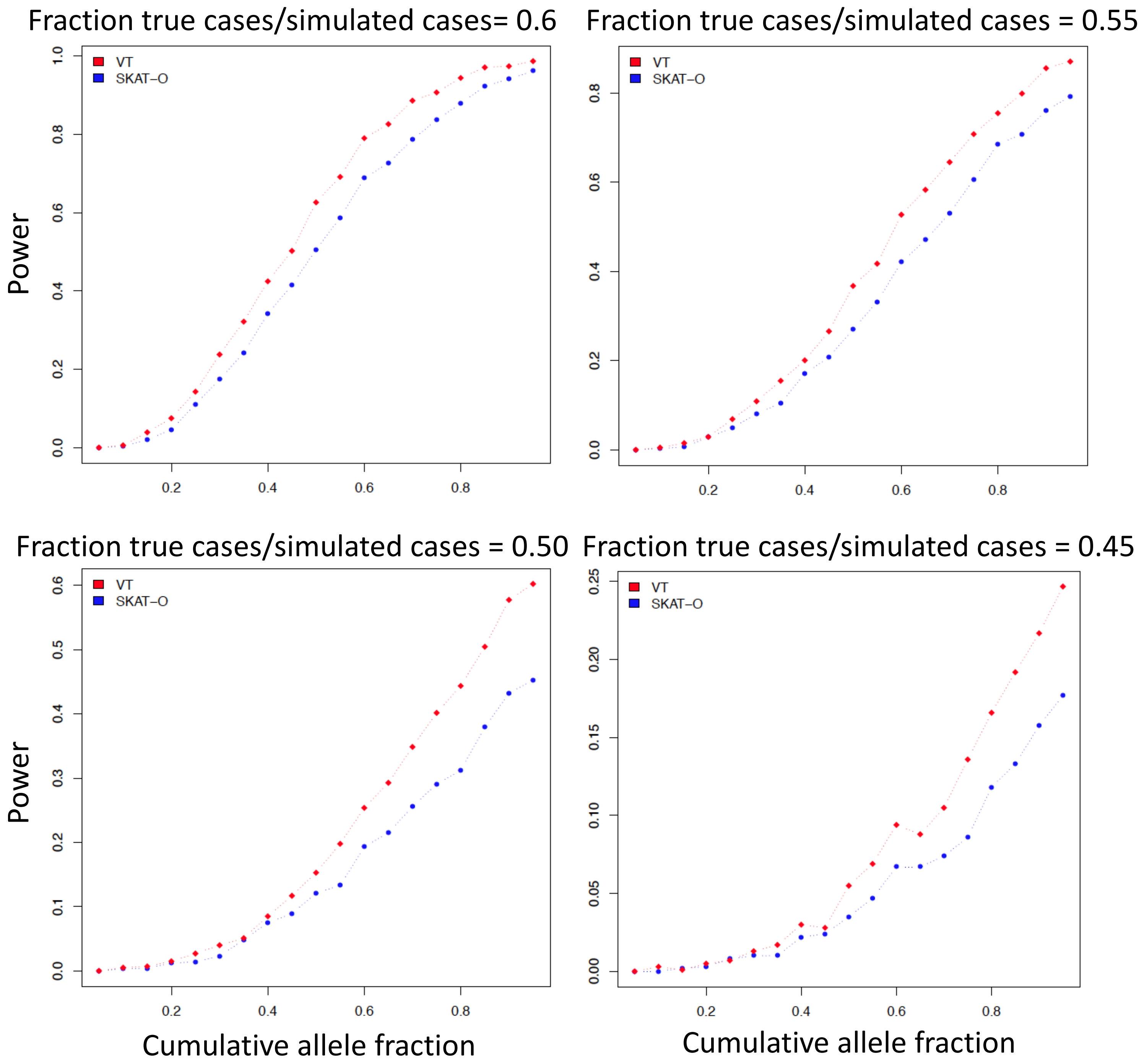

Fraction true cases/simulated cases= 0.6
Fraction true cases/simulated cases = 0.55
Fraction true cases/simulated cases = 0.50
Fraction true cases/simulated cases = 0.45
VT
SKAT–O
Power
Cumulative allele fraction
