# Supplementary material for: Novel risk genes and mechanisms implicated by exome sequencing of 2572 individuals with pulmonary arterial hypertension
Source: Genome Med. 2019 Nov 14;11:69. doi: 10.1186/s13073-019-0685-z (PMC6857288; doi:10.1186/s13073-019-0685-z)
Supplement: Supplementary file 4 — Additional file 4: Table S2. PAH Biobank cohort demographic data by PAH subclass. [file 13073_2019_685_MOESM4_ESM.docx]

**Table S2. PAH Biobank cohort demographic and hemodynamic data by PAH subclass.**

|  | **ALL** | **IPAH** | **APAH** | | | | **DTOX** | **FPAH** | **Other** |
| --- | --- | --- | --- | --- | --- | --- | --- | --- | --- |
|  |  |  | **CTD** | **CHD** | **Portopulmonary** | **Other** |  |  |  |
| **Total, n (%)** | 2572 | 1110 (43.2) | 722 (28.1) | 268 (10.4) | 139 (5.4) | 110 (4.3) | 110 (4.3) | 98 (3.8) | 12 (0.5) |
| **Age-of-onset** |  |  |  |  |  |  |  |  |  |
| Child (dx age <19) | 226 (8.8) | 94 (8.5) | 7 (0.9) | 95 (37.2)*** | 4 (2.8) | 6 (5.5) | 1 (0.9) | 13 (13.4) | 4 (33.3) |
| Adult (dx age >=19) | 2345 (91.2) | 1015 (91.4) | 715 (99.0) | 173 (62.8) | 135 (97.2) | 104 (94.5) | 109 (99.1) | 85 (86.6) | 8 (66.7) |
| **Gender** |  |  |  |  |  |  |  |  |  |
| Female | 2023 (78.7) | 878 (78.4) | 655 (90.7) | 200 (74.5) | 63 (46.1) | 78 (70.9) | 81 (73.9) | 67 (68.2) | 9 (75.0) |
| Male | 548 (21.3) | 242 (21.6) | 67 (9.3) | 68 (25.5) | 76 (53.9) | 32 (29.1) | 29 (26.1) | 31 (31.6) | 3 (25.0) |
| Female:male ratio | 3.7:1 | 3.6:1 | 9.8:1 | 2.9:1 | 1:1.2 | 2.4:1 | 2.8:1 | 2.1:1 | 3:1 |
| **Ancestry n (%)** |  |  |  |  |  |  |  |  |  |
| European | 1851 (72) | 816 (73) | 491 (68) | 190 (71) | 110 (79) | 63 (57.3) | 88 (80) | 87 (89) | 11 (91.7) |
| Hispanic | 316 (12) | 138 (12) | 74 (10) | 40 (15) | 25 (18)* | 18 (16.4) | 12 (11) | 9 (9.2) | 0 |
| African | 292 (11) | 118 (11) | 132 (18)* | 8 (3)** | 3 (2.2)** | 25 (22.7) | 5 (4.5) | 1 (1) | 1 (8.3) |
| East Asian | 70 (2.7) | 25 (2.2) | 20 (2.8) | 18 (6.7) | 1 (0.72) | 2 (1.8) | 4 (3.6) | 0 | 0 |
| South Asian | 28 (1.1) | 13 (1.2) | 4 (0.55) | 9 (3.4) | 0 | 2 (1.8) | 0 | 1 (1) | 0 |
| Others | 15 (0.58) | 10 (0.89) | 1 (0.14) | 3 (1.1) | 0 | 0 | 1 (0.91) | 0 | 0 |
| **Hemodynamics, mean ± SD (n)** |  |  |  |  |  |  |  |  |  |
| MPAP (mmHg) |  | 52 ± 14 | 44 ± 12*** | 55 ± 19** | 49 ± 12 | 50 ± 13 | 52 ± 12 | 59 ± 14*** | 41 ± 12 |
| MPCW (mmHg) |  | 10 ± 4 (1078) | 10 ± 4 (708) | 10 ± 4 (251) | 10 ± 4 (136) | 10 ± 4 (91) | 11 ± 4 (91) | 10 ± 4 (95) | 11 ± 3 (12) |
| CO, Fick (L/min) |  | 4.4 ± 1.7 (770) | 4.7 ± 1.7 (538) | 4.1 ± 2.1 (191) | 5.6 ± 2.0 (98)*** | 4.8 ± 2.6 (67) | 4.3 ± 1.3 (77) | 3.6 ± 1.0(66)** | 4.1 ± 1.7 (10) |
| PVR (Woods units) |  | 11.2 ± 6.9 (747) | 8.4 ± 5.0 (529)*** | 14.9 ± 10.0 (181)*** | 7.5 ± 4.1 (95)*** | 10.3 ± 5.6 (67) | 11.2 ± 6.8 (73) | 15.2 ± 6.2 (64)*** | 8.9 ± 3.3 (9) |

Abbreviations: IPAH, idiopathic PAH; APAH, PAH associated with other diseases; CTD, connective tissue disorders; CHD, congenital heart disease; DTOX, diet and toxin-induced PAH; FPAH, familial PAH; dx, diagnosis; SD, standard deviation; MPAP, mean pulmonary artery pressure; MPCW, mean pulmonary capillary wedge pressure; CO, cardiac output; PVR, pulmonary vascular resistance.

APAH other included HIV, HHT and other rare associated diseases.

ALL other included 11 non-familial PVOD/PCH and one persistent pulmonary hypertension of the newborn.

*p=0.02, two-tailed Chi-square test (all Hispanic or African APAH cases vs Hispanic or African portopulmonary or CTD cases).

**p≤0.001, two-tailed Chi-square test (all African APAH cases vs African CHD or portopulmonary cases).

***<0.0001, two-tailed Chi-square test (all child-onset cases vs child-onset CHD cases).

For hemodynamic data: *p<0.05, **p<0.01, ***p<0.0001, one-way ANOVA with correction for multiple comparisons vs IPAH.
