# Supplementary material for: Novel risk genes and mechanisms implicated by exome sequencing of 2572 individuals with pulmonary arterial hypertension
Source: Genome Med. 2019 Nov 14;11:69. doi: 10.1186/s13073-019-0685-z (PMC6857288; doi:10.1186/s13073-019-0685-z)
Supplement: Supplementary file 5 — Additional file 5: Table S3. Rare, predicted deleterious variants in established PAH risk genes among 2572 PAH cases; Table S4. Rare, predicted deleterious variants in recently reported PAH risk genes among 2572 PAH cases. [file 13073_2019_685_MOESM5_ESM.docx]

**Table S3. Rare, predicted deleterious variants* in established PAH risk genes among 2,572 PAH cases. Patients are heterozygous for the indicated variant unless noted.**

| **ID** | **PAH subclass** | **Gender** | **Age dx** | **Ancestry** | **Gene** | **Transcript** | **Nucleotide change** | **Amino acid change** | **Variant type** | **Previously reported?** | **MAF (ExAC)** | **CADD** | **REVEL** |
| --- | --- | --- | --- | --- | --- | --- | --- | --- | --- | --- | --- | --- | --- |
| 12-070 | APAH-HHT | F | 35 | EUR | *ACVRL1* | . | exon 10 del | . | exon deletion | no | N/A | . | . |
| 12-193 | APAH-HHT | F | 59 | EUR | *ACVRL1* | NM_001077401.1 | c.199C>T | p.(Arg67Trp) | D-Mis | ^1-3^ | . | 24 | 0.72 |
| 26-020 | DTOX | F | 35 | EUR | *ACVRL1* | NM_001077401.1 | c.430C>T | p.(Arg144*) | stopgain | ^2^ | 8.57E-06 | 39 | . |
| 07-010 | APAH-NF | F | 46 | EUR | *ACVRL1* | NM_001077401.1 | c.599G>A | p.(Arg200Gln) | D-Mis | rs1018788708 | . | 35 | 0.78 |
| 03-069 | APAH-CHD | F | 68 | EUR | *ACVRL1* | NM_001077401.1 | c.641G>T | p.(Gly214Val) | D-Mis | no | . | 28 | 0.83 |
| 08-069 | APAH-CTD | F | 53 | EUR | *ACVRL1* | NM_001077401.1 | c.721C>T | p.(Arg241Trp) | D-Mis | no | 5.09E-05 | 35 | 0.84 |
| 02-100* | FPAH | F | 55 | EUR | *ACVRL1* | NM_001077401.1 | c.841G>C | p.(Glu281Gln) | D-Mis | no | . | 30 | 0.81 |
| 12-015* | IPAH | F | 40 | EUR | *ACVRL1* | NM_001077401.1 | c.864T>G | p.(Phe288Leu) | D-Mis | no | . | 25 | 0.66 |
| 10-045 | APAH-HHT | F | 29 | EUR | *ACVRL1* | NM_001077401.1 | c.950T>C | p.(Ile317Thr) | D-Mis | ^2, 4, 5^ | . | 27 | 0.92 |
| 07-022 | APAH-CHD | M | 52 | EUR | *ACVRL1* | NM_001077401.1 | c.1064A>C | p.(His355Pro) | D-Mis | no | 8.39E-06 | 27 | 0.68 |
| 12-058 | APAH-HHT | F | 48 | EUR | *ACVRL1* | NM_001077401.1 | c.1120C>T | p.(Arg374Trp) | D-Mis | ^1, 6^ | . | 29 | 0.70 |
| 05-004 | IPAH | F | 46 | EUR | *ACVRL1* | NM_001077401.1 | c.1258G>A | p.(Asp420Asn) | D-Mis | no | . | 34 | 0.74 |
| 15-080 | IPAH | M | 14 | AMR | *ACVRL1* | NM_001077401.1 | c.1270C>A | p.(Pro424Thr) | D-Mis | ^2, 4, 7^ | . | 32 | 0.79 |
| 11-048 | APAH-HHT | F | 46 | EUR | *ACVRL1* | NM_001077401.1 | c.1331_1332dup | p.(Asp445Trpfs*21) | frameshift | no | . | 35 | . |
| 12-108 | APAH-HHT | F | 56 | SAS | *ACVRL1* | NM_001077401.1 | c.1345C>T | p.(Pro449Ser) | D-Mis | ^1^ | . | 33 | 0.74 |
| 01-018 | APAH-HHT | F | 4 | EUR | *ACVRL1* | NM_001077401.1 | c.1450C>T | p.(Arg484Trp) | D-Mis | ^2, 6, 8, 9^ | . | 33 | 0.92 |
| 22-065 | IPAH | F | 50 | EAS | *BMPR1A* | NM_004329.2 | c.407C>T | p.(Pro136Leu) | D-Mis | no | . | 27 | 0.83 |
| 22-110 | IPAH | F | 52 | AFR | *BMPR1A* | NM_004329.2 | c.1216C>T | p.(Arg406Cys) | D-Mis | rs587781332 | 2.47E-05 | 35 | 0.65 |
| 20-017 | APAH-HIV | F | 40 | AFR | *BMPR1A* | NM_004329.2 | c.1324C>T | p.(Arg442Cys) | D-Mis | rs587782496 | . | 35 | 0.74 |
| 06-130 | APAH-CHD | F | 27 | EUR | *BMPR1A* | NM_004329.2 | c.1498A>G | p.(Met500Val) | D-Mis | no | 4.94E-05 | 25 | 0.63 |
| 05-035 | APAH-CTD | M | 57 | AMR | *BMPR1B* | NM_001256792.1 | c.334C>T | p.(Pro112Ser) | D-Mis | no | . | 26 | 0.56 |
| 30-038 | APAH-Portopulm | F | 51 | AMR | *BMPR1B* | NM_001256792.1 | c.1253G>A | p.(Gly418Asp) | D-Mis | no | . | 33 | 0.77 |
| 04-012 | IPAH | M | 43 | EUR | *BMPR2* | NM_001204.6 | c.7del | p.(Ser3Profs*44) | frameshift | no | . | 24 | . |
| 04-026 | IPAH | F | 31 | EUR | *BMPR2* | NM_001204.6 | c.16C>T | p.(Gln6*) | stopgain | ^2, 10-13^ | . | 35 | . |
| 06-070 | IPAH | F | 28 | EUR | *BMPR2* | NM_001204.6 | c.16C>T | p.(Gln6*) | stopgain | ^2, 10-13^ | . | 35 | . |
| 08-097 | IPAH | M | 42 | EUR | *BMPR2* | NM_001204.6 | c.39G>A | p.(Trp13*) | stopgain | ^2^ | . | 36 | . |
| 02-072 | IPAH | F | 40 | AFR | *BMPR2* | NM_001204.6 | c.47G>A | p.(Trp16*) | stopgain | ^2, 14, 15^ | . | 35 | . |
| 06-027 | IPAH | F | 59 | EUR | *BMPR2* | NM_001204.6 | c.53_64del | p.(Ile18_Val21del) | in-frame | no | . | 18 | . |
| 15-067 | FPAH | F | 8 | SAS | *BMPR2* | NM_001204.6 | c.76+1G>T | p.(=) | splicing | ^2^ | . | 23 | . |
| 08-054 | FPAH | F | 37 | EUR | *BMPR2* | NM_001204.6 | c.76+2T>C | p.(=) | splicing | ^2^ | . | 23 | . |
| 11-049 | IPAH | F | 33 | EUR | *BMPR2* | NM_001204.6 | c.116del | p.(Pro39Argfs*8) | frameshift | no | . | 33 | . |
| 10-096 | IPAH | F | 60 | EUR | *BMPR2* | NM_001204.6 | c.118dup | p.(Tyr40Leufs*9) | frameshift | no | . | 32 | . |
| 02-071* | FPAH | F | 40 | EUR | *BMPR2* | NM_001204.6 | c.135dup | p.(Ile46Aspfs*3) | frameshift | no | . | . | . |
| 17-077 | IPAH | F | 38 | AMR | *BMPR2* | NM_001204.6 | c.164dup | p.(Asn55Lysfs*10) | frameshift | no | . | 33 | . |
| 22-034 | IPAH | F | 36 | AFR | *BMPR2* | NM_001204.6 | c.199dup | p.(Tyr67Leufs*31) | frameshift | no | . | 33 | . |
| 12-115 | FPAH | M | 28 | EUR | *BMPR2* | NM_001204.6 | c.200A>G | p.(Tyr67Cys) | D-Mis | ^2, 6, 8, 13, 16-18^ | . | 26 | 0.89 |
| 06-010 | FPAH | M | 56 | EUR | *BMPR2* | NM_001204.6 | c.201T>G | p.(Tyr67*) | stopgain | no | . | 35 | . |
| 06-011 | FPAH | F | 41 | EUR | *BMPR2* | NM_001204.6 | c.201T>G | p.(Tyr67*) | stopgain | no | . | 35 | . |
| 17-002 | IPAH | F | 34 | EAS | *BMPR2* | NM_001204.6 | c.203del | (p.(Gly68Alafs*10) | frameshift | no | . | 33 | . |
| 09-059 | IPAH | F | 44 | EUR | *BMPR2* | NM_001204.6 | c.211del | p.(Glu71Argfs*7) | frameshift | no | . | 33 | . |
| 06-116 | APAH-CHD | F | 4 | EUR | *BMPR2* | NM_001204.6 | c.211G>A | p.(Glu71Lys) | D-Mis | ^19^ | . | 26 | 0.75 |
| 27-004 | IPAH | F | 33 | EUR | *BMPR2* | NM_001204.6 | c.218C>A | p.(Ser73*) | stopgain | no | . | 36 | . |
| 05-094 | IPAH | F | 24 | AMR | *BMPR2* | NM_001204.6 | c.251G>A | p.(Cys84Tyr) | D-Mis | ^8^ | . | 28 | 0.95 |
| 22-054 | IPAH | F | 37 | EUR | *BMPR2* | NM_001204.6 | c.255G>A | p.(Trp85*) | stopgain | ^2, 10, 18^ | . | 38 | . |
| 32-007 | APAH-CTD | F | 68 | EUR | *BMPR2* | NM_001204.6 | c.255G>A | p.(Trp85*) | stopgain | ^2, 10, 18^ | . | 38 | . |
| 03-003 | IPAH | M | 49 | EUR | *BMPR2* | NM_001204.6 | c.258del | p.(His87Thrfs*14) | frameshift | no | . | 33 | . |
| 08-048 | IPAH | M | 44 | EUR | *BMPR2* | NM_001204.6 | c.277dup | p.(Glu93Glyfs*5) | frameshift | no | . | 34 | . |
| 04-007 | IPAH | F | 21 | EUR | *BMPR2* | NM_001204.6 | c.278_279del | p.(Glu93Valfs*4) | frameshift | no | . | 34 | . |
| 15-051* | IPAH | F | 4 | EUR | *BMPR2* | NM_001204.6 | c.295T>C | p.(Cys99Arg) | D-Mis | ^2, 6, 18, 20, 21^ | . | 26 | 0.94 |
| 06-022 | IPAH | M | 3 | EUR | *BMPR2* | NM_001204.6 | c.297T>G | p.(Cys99Trp) | D-Mis | ^6^ | . | 26 | 0.93 |
| 16-032 | IPAH | F | 21 | EUR | *BMPR2* | NM_001204.6 | c.344del | p.(Phe115Serfs*37) | frameshift | no | . | 33 | . |
| 16-050 | FPAH | F | 41 | EUR | *BMPR2* | NM_001204.6 | c.344del | p.(Phe115Serfs*37) | frameshift | no | . | 33 | . |
| 12-088 | FPAH | F | 29 | EUR | *BMPR2* | NM_001204.6 | c.350G>A | p.(Cys117Tyr) | D-Mis | ^2, 6, 21, 22^ | . | 28 | 0.96 |
| 19-032 | FPAH | F | 28 | EUR | *BMPR2* | NM_001204.6 | c.350G>A | p.(Cys117Tyr) | D-Mis | ^2, 6, 21, 22^ | . | 28 | 0.96 |
| 19-010 | IPAH | M | 33 | EUR | *BMPR2* | NM_001204.6 | c.353G>A | p.(Cys118Tyr) | D-Mis | ^2, 23, 24^ | . | 28 | 0.94 |
| 19-003 | FPAH | F | 28 | EUR | *BMPR2* | NM_001204.6 | c.354T>G | p.(Cys118Trp) | D-Mis | ^2, 25^ | . | 26 | 0.93 |
| 19-070 | IPAH | F | 30 | EUR | *BMPR2* | NM_001204.6 | c.354T>G | p.(Cys118Trp) | D-Mis | ^2, 25^ | . | 26 | 0.93 |
| 19-077 | FPAH | F | 39 | EUR | *BMPR2* | NM_001204.6 | c.354T>G | p.(Cys118Trp) | D-Mis | ^2, 25^ | . | 26 | 0.93 |
| 19-088 | FPAH | F | 26 | EUR | *BMPR2* | NM_001204.6 | c.354T>G | p.(Cys118Trp) | D-Mis | ^2, 25^ | . | 26 | 0.93 |
| 25-001 | FPAH | M | 3 | EUR | *BMPR2* | NM_001204.6 | c.354T>G | p.(Cys118Trp) | D-Mis | ^2, 25^ | . | 26 | 0.93 |
| 12-095 | IPAH | M | 45 | EUR | *BMPR2* | NM_001204.6 | c.367T>C | p.(Cys123Arg) | D-Mis | ^2, 7, 8, 16, 23^ | . | 27 | 0.93 |
| 12-101 | IPAH | M | 64 | EUR | *BMPR2* | NM_001204.6 | c.367T>C | p.(Cys123Arg) | D-Mis | ^2, 7, 8, 16, 23^ | . | 27 | 0.93 |
| 12-197 | FPAH | M | 39 | EUR | *BMPR2* | NM_001204.6 | c.377A>G | p.(Asn126Ser) | D-Mis | ^2, 8, 12, 16, 21, 24, 26^ | . | 26 | 0.80 |
| 19-062 | IPAH | F | 48 | EUR | *BMPR2* | NM_001204.6 | c.377A>G | p.(Asn126Ser) | D-Mis | ^2, 8, 12, 16, 21, 24, 26^ | . | 26 | 0.80 |
| 13-045 | DTOX | M | 26 | EUR | *BMPR2* | NM_001204.6 | c.419-1G>T | p.(=) | splicing | no | . | 26 | . |
| 02-100* | FPAH | F | 55 | EUR | *BMPR2* | NM_001204.6 | c.439C>T | p.(Arg147*) | stopgain | ^2, 6, 8, 13, 16-18, 20, 23, 24, 26-28^ | . | 38 | . |
| 20-011 | IPAH | F | 36 | EUR | *BMPR2* | NM_001204.6 | c.439C>T | p.(Arg147*) | stopgain | ^2, 6, 8, 13, 16-18, 20, 23, 24, 26-28^ | . | 38 | . |
| 26-029 | IPAH | F | 21 | EUR | *BMPR2* | NM_001204.6 | c.452_453del | p.(Ile151Asnfs*29) | stopgain | no | . | 34 | . |
| 26-039 | IPAH | F | 31 | EUR | *BMPR2* | NM_001204.6 | c.524del | p.(Leu175*) | stopgain | no | . | 34 | . |
| 03-097 | IPAH | F | 31 | EUR | *BMPR2* | NM_001204.6 | c.529+1G>A | p.(=) | splicing | ^19^ | . | 28 | . |
| 18-038 | IPAH | F | 54 | EUR | *BMPR2* | NM_001204.6 | c.529+1G>A | p.(=) | splicing | ^19^ | . | 28 | . |
| 02-119 | IPAH | F | 53 | EUR | *BMPR2* | NM_001204.6 | c.529G>A | p.(Gly177Arg) | D-Mis | no | . | 23 | 0.55 |
| 18-078 | IPAH | F | 21 | AFR | *BMPR2* | NM_001204.6 | c.543_544del | p.(Gly182Serfs*17) | frameshift | no | . | 35 | . |
| 03-110 | IPAH | F | 36 | AFR | *BMPR2* | NM_001204.6 | c.631C>T | p.(Arg211*) | stopgain | ^2, 6, 8, 18^ ^16, 20-24, 29, 30^ | . | 42 | . |
| 21-054 | IPAH | F | 48 | EUR | *BMPR2* | NM_001204.6 | c.631C>T | p.(Arg211*) | stopgain | ^2, 6, 8, 18^ ^16, 20-24, 29, 30^ | . | 42 | . |
| 22-001 | IPAH | F | 55 | EUR | *BMPR2* | NM_001204.6 | c.631C>T | p.(Arg211*) | stopgain | ^2, 6, 8, 18^ ^16, 20-24, 29, 30^ | . | 42 | . |
| 06-015 | FPAH | F | 60 | EUR | *BMPR2* | NM_001204.6 | c.637C>T | p.(Arg213*) | stopgain | ^2, 8, 13, 18, 20^ | . | 37 | . |
| 10-012 | FPAH | F | 30 | EUR | *BMPR2* | NM_001204.6 | c.637C>T | p.(Arg213*) | stopgain | ^2, 8, 13, 18, 20^ | . | 37 | . |
| 12-015* | IPAH | F | 40 | EUR | *BMPR2* | NM_001204.6 | c.637C>T | p.(Arg213*) | stopgain | ^2, 8, 13, 18, 20^ | . | 37 | . |
| 26-037 | FPAH | F | 26 | EUR | *BMPR2* | NM_001204.6 | c.637C>T | p.(Arg213*) | stopgain | ^2, 8, 13, 18, 20^ | . | 37 | . |
| 16-061 | IPAH | F | 37 | Dominican | *BMPR2* | NM_001204.6 | c.688A>T | p.(Lys230*) | stopgain | no | . | 40 | . |
| 16-041 | IPAH | F | 42 | EUR | *BMPR2* | NM_001204.6 | c.689_690del | p.(Lys230Serfs*25) | frameshift | no | . | 34 | . |
| 08-074 | FPAH | F | 19 | EUR | *BMPR2* | NM_001204.6 | c.697del | p.(Cys397*) | stopgain | no | . | 35 | . |
| 10-100 | IPAH | M | 34 | EUR | *BMPR2* | NM_001204.6 | c.712C>T | p.(Gln238*) | stopgain | no | . | 39 | . |
| 07-011 | APAH-CTD | F | 65 | EUR | *BMPR2* | NM_001204.6 | c.775C>T | p.(Arg259Cys) | D-Mis | no | 4.95E-05 | 34 | 0.78 |
| 12-208 | APAH-unspecified | F | 38 | AMR | *BMPR2* | NM_001204.6 | c.797G>C | p.(Arg266Thr) | D-Mis | ^2, 18, 21^ | 4.13E-05 | 28 | 0.63 |
| 05-046 | IPAH | F | 32 | EUR | *BMPR2* | NM_001204.6 | c.834dup | p.(Met279Aspfs*19) | frameshift | no | . | 35 | . |
| 07-060 | FPAH | F | 34 | EUR | *BMPR2* | NM_001204.6 | c.846T>G | p.(Tyr282*) | stopgain | rs863223419 | . | 36 | . |
| 03-058 | IPAH | F | 39 | EUR | *BMPR2* | NM_001204.6 | c.852+1G>A | p.(=) | splicing | ^2, 16^ | . | 27 | . |
| 19-014 | FPAH | M | 37 | EUR | *BMPR2* | NM_001204.6 | c.852+1G>A | p.(=) | splicing | ^2, 16^ | . | 27 | . |
| 19-019 | FPAH | F | 48 | EUR | *BMPR2* | NM_001204.6 | c.852+1G>A | p.(=) | splicing | ^2, 16^ | . | 27 | . |
| 05-008 | IPAH | M | 40 | EUR | *BMPR2* | NM_001204.6 | c.853-1G>A | p.(=) | splicing | ^2, 8^ | . | 26 | . |
| 07-077 | FPAH | M | 43 | EUR | *BMPR2* | NM_001204.6 | c.853-2A>G | p.(=) | splicing | ^2, 8, 10, 11, 18^ | . | 25 | . |
| 08-104 | FPAH | F | 26 | EUR | *BMPR2* | NM_001204.6 | c.862dup | p.(Cys288Leufs*10) | frameshift | no | . | 33 | . |
| 05-052 | APAH-CHD | F | 32 | AMR | *BMPR2* | NM_001204.6 | c.872dup | p.(Leu291Phefs*7) | frameshift | no | . | 35 | . |
| 08-032 | IPAH | F | 51 | EUR | *BMPR2* | NM_001204.6 | c.893G>A | p.(Trp298*) | stopgain | ^2, 31^ | . | 37 | . |
| 19-017 | FPAH | M | 33 | EUR | *BMPR2* | NM_001204.6 | c.894_895dup | p.(Val299Glyfs*2) | frameshift | no | . | 35 | . |
| 28-136 | IPAH | F | 32 | EUR | *BMPR2* | NM_001204.6 | c.918_921del | p.(His306Glnfs*28) | frameshift | no | . | 35 | . |
| 07-096 | IPAH | F | 28 | AMR | *BMPR2* | NM_001204.6 | c.935T>C | p.(Leu312Pro) | D-Mis | no | . | 29 | 0.98 |
| 16-017 | IPAH | F | 41 | AMR | *BMPR2* | NM_001204.6 | c.942_943insA | p.(Leu315Thrfs*12) | frameshift | no | . | 35 | . |
| 17-049 | IPAH | F | 35 | EUR | *BMPR2* | NM_001204.6 | c.947A>G | p.(His316Arg) | D-Mis | no | . | 25 | 0.93 |
| 11-018 | APAH-CTD | F | 33 | EUR | *BMPR2* | NM_001204.6 | c.961C>T | p.(Arg321*) | stopgain | ^2, 6, 8, 16-18, 24, 32^ | . | 40 | . |
| 13-083 | FPAH | F | 62 | EUR | *BMPR2* | NM_001204.6 | c.961C>T | p.(Arg321*) | stopgain | ^2, 6, 8, 16-18, 24, 32^ | . | 40 | . |
| 17-027 | IPAH | F | 31 | EUR | *BMPR2* | NM_001204.6 | c.961C>T | p.(Arg321*) | stopgain | ^2, 6, 8, 16-18, 24, 32^ | . | 40 | . |
| 18-043 | IPAH | M | 30 | AMR | *BMPR2* | NM_001204.6 | c.961C>T | p.(Arg321*) | stopgain | ^2, 6, 8, 16-18, 24, 32^ | . | 40 | . |
| 37-011 | IPAH | M | 69 | EUR | *BMPR2* | NM_001204.6 | c.961C>T | p.(Arg321*) | stopgain | ^2, 6, 8, 16-18, 24, 32^ | . | 40 | . |
| 15-077 | IPAH | F | 16 | EUR | *BMPR2* | NM_001204.6 | c.967G>A | p.(Asp323Asn) | D-Mis | no | . | 32 | 0.60 |
| 12-084 | FPAH | M | 45 | EUR | *BMPR2* | NM_001204.6 | c.969dup | p.(His324Serfs*3) | frameshift | no | . | 34 | . |
| 05-027 | IPAH | M | 37 | EUR | *BMPR2* | NM_001204.6 | c.994C>T | p.(Arg332*) | stopgain | ^6, 8, 16, 20, 22, 23, 26, 30, 33, 34^ | . | 38 | . |
| 08-082 | IPAH | F | 35 | EUR | *BMPR2* | NM_001204.6 | c.994C>T | p.(Arg332*) | stopgain | ^6, 8, 16, 20, 22, 23, 26, 30, 33, 34^ | . | 38 | . |
| 10-018 | FPAH | M | 54 | EUR | *BMPR2* | NM_001204.6 | c.994C>T | p.(Arg332*) | stopgain | ^6, 8, 16, 20, 22, 23, 26, 30, 33, 34^ | . | 38 | . |
| 19-002 | FPAH | M | 26 | EUR | *BMPR2* | NM_001204.6 | c.994C>T | p.(Arg332*) | stopgain | ^6, 8, 16, 20, 22, 23, 26, 30, 33, 34^ | . | 38 | . |
| 19-021 | FPAH | F | 14 | EUR | *BMPR2* | NM_001204.6 | c.994C>T | p.(Arg332*) | stopgain | ^6, 8, 16, 20, 22, 23, 26, 30, 33, 34^ | . | 38 | . |
| 25-009 | FPAH | M | 9 | EUR | *BMPR2* | NM_001204.6 | c.994C>T | p.(Arg332*) | stopgain | ^6, 8, 16, 20, 22, 23, 26, 30, 33, 34^ | . | 38 | . |
| 02-045 | FPAH | F | 48 | EUR | *BMPR2* | NM_001204.6 | c.995G>C | p.(Arg332Pro) | D-Mis | ^6^ | . | 34 | 0.99 |
| 06-013 | IPAH | F | 31 | EUR | *BMPR2* | NM_001204.6 | c.1040G>A | p.(Cys347Tyr) | D-Mis | ^2, 6, 18, 25^ | . | 29 | 0.94 |
| 19-090 | IPAH | F | 53 | EUR | *BMPR2* | NM_001204.6 | c.1126G>T | p.(Glu376*) | stopgain | ^2^ | . | 45 | . |
| 10-031 | FPAH | F | 22 | EUR | *BMPR2* | NM_001204.6 | c.1128del | p.(Val377Leufs*12) | frameshift | no | . | 26 | . |
| 10-032 | FPAH | F | 53 | EUR | *BMPR2* | NM_001204.6 | c.1128del | p.(Val377Leufs*12) | frameshift | no | . | 26 | . |
| 05-136 | FPAH | F | 22 | AMR | *BMPR2* | NM_001204.6 | c.1128+1G>A | p.(=) | splicing | ^2, 18^ | . | 27 | . |
| 06-007 | IPAH | M | 26 | EUR | *BMPR2* | NM_001204.6 | c.1128+1G>A | p.(=) | splicing | ^2, 18^ | . | 27 | . |
| 15-059 | FPAH | F | 16 | AMR | *BMPR2* | NM_001204.6 | c.1128+1G>A | p.(=) | splicing | ^2, 18^ | . | 27 | . |
| 03-113 | IPAH | M | 30 | EUR | *BMPR2* | NM_001204.6 | c.1128+1G>C | p.(=) | splicing | ^2, 18^ | . | 26 | . |
| 07-075 | FPAH | F | 35 | EUR | *BMPR2* | NM_001204.6 | c.1141dup | p.(Arg381Lysfs*18) | frameshift | no | . | 34 | . |
| 06-059 | IPAH | F | 7 | EAS | *BMPR2* | NM_001204.6 | c.1154C>G | p.(Pro385Arg) | D-Mis | ^6^ | . | 28 | 0.98 |
| 04-008 | IPAH | M | 34 | AFR | *BMPR2* | NM_001204.6 | c.1172C>A | p.(Ala391Asp) | D-Mis | no | . | 33 | 0.82 |
| 25-005 | APAH-CHD | F | 10 | EUR | *BMPR2* | NM_001204.6 | c.1175T>A | p.(Val392Glu) | D-Mis | no | . | 32 | 0.91 |
| 09-038 | IPAH | F | 27 | AFR | *BMPR2* | NM_001204.6 | c.1191_1192del | p.(Cys397*) | stopgain | no | . | 35 | . |
| 10-049 | IPAH | F | 57 | EUR | *BMPR2* | NM_001204.6 | c.1197del | p.(Ala400Leufs*2) | frameshift | no | . | 35 | . |
| 13-035 | FPAH | F | 35 | AFR | *BMPR2* | NM_001204.6 | c.1233_1236dup | p.(Tyr413Asnfs*36) | frameshift | no | . | 35 | . |
| 17-013 | FPAH | F | 38 | EUR | *BMPR2* | NM_001204.6 | c.1250_1253del | p.(Phe417*) | stopgain | no | . | 34 | . |
| 08-037 | IPAH | F | 67 | EUR | *BMPR2* | NM_001204.6 | c.1361C>T | p.(Ser454Phe) | D-Mis | no | . | 33 | 0.52 |
| 12-085 | IPAH | F | 63 | EUR | *BMPR2* | NM_001204.6 | c.1361C>T | p.(Ser454Phe) | D-Mis | no | . | 33 | 0.52 |
| 10-005 | FPAH | F | 44 | EUR | *BMPR2* | NM_001204.6 | c.1397G>A | p.(Trp466*) | stopgain | ^18, 32^ ^20, 24^ | . | 43 | . |
| 08-022 | IPAH | F | 60 | EUR | *BMPR2* | NM_001204.6 | c.1413+1G>A | p.(=) | splicing | ^2, 16, 24^ | . | 25 | . |
| 19-009 | IPAH | F | 29 | EUR | *BMPR2* | NM_001204.6 | c.1450T>C | p.(Trp484Arg) | D-Mis | no | . | 28 | 0.79 |
| 08-003 | FPAH | F | 48 | EUR | *BMPR2* | NM_001204.6 | c.1451G>A | p.(Trp484*) | stopgain | ^2^ | . | 40 | . |
| 08-068 | FPAH | F | 30 | EUR | *BMPR2* | NM_001204.6 | c.1451G>A | p.(Trp484*) | stopgain | ^2^ | . | 40 | . |
| 33-009 | IPAH | F | 25 | AFR | *BMPR2* | NM_001204.6 | c.1451G>A | p.(Trp484*) | stopgain | ^2^ | . | 40 | . |
| 33-012 | IPAH | F | 58 | AFR | *BMPR2* | NM_001204.6 | c.1451G>A | p.(Trp484*) | stopgain | ^2^ | . | 40 | . |
| 04-083 | IPAH | F | 40 | EUR | *BMPR2* | NM_001204.6 | c.1460A>T | p.(Asp487Val) | D-Mis | ^2, 21, 24^ | . | 29 | 0.95 |
| 05-194 | IPAH | F | 61 | EUR | *BMPR2* | NM_001204.6 | c.1468G>A | p.(Ala490Thr) | D-Mis | no | . | 33 | 0.52 |
| 39-001 | IPAH | F | 41 | AFR | *BMPR2* | NM_001204.6 | c.1471C>T | p.(Arg491Trp) | D-Mis | ^2, 16-18, 32, 33, 35-37^ ^6, 8, 11, 12, 24^ | . | 35 | 0.96 |
| 03-108 | FPAH | F | 30 | EUR | *BMPR2* | NM_001204.6 | c.1472G>A | p.(Arg491Gln) | D-Mis | ^2, 5, 6, 8, 18^ ^24, 26, 34, 35^ | . | 35 | 0.96 |
| 06-047 | IPAH | M | 11 | AMR | *BMPR2* | NM_001204.6 | c.1472G>A | p.(Arg491Gln) | D-Mis | ^2, 5, 6, 8, 18^ ^24, 26, 34, 35^ | . | 35 | 0.96 |
| 08-020 | IPAH | M | 52 | EUR | *BMPR2* | NM_001204.6 | c.1472G>A | p.(Arg491Gln) | D-Mis | ^2, 5, 6, 8, 18^ ^24, 26, 34, 35^ | . | 35 | 0.96 |
| 19-018 | IPAH | M | 43 | EUR | *BMPR2* | NM_001204.6 | c.1472G>A | p.(Arg491Gln) | D-Mis | ^2, 5, 6, 8, 18^ ^24, 26, 34, 35^ | . | 35 | 0.96 |
| 02-155 | IPAH | F | 13 | AMR | *BMPR2* | NM_001204.6 | c.1483C>T | p.(Gln495*) | stopgain | ^2, 24, 32^ | . | 41 | . |
| 03-107 | APAH-CTD | F | 76 | EUR | *BMPR2* | NM_001204.6 | c.1492G>A | p.(Glu498Lys) | D-Mis | no | . | 34 | 0.70 |
| 29-006 | FPAH | F | 36 | EUR | *BMPR2* | NM_001204.6 | c.1524G>A | p.(Trp508*) | stopgain | ^2, 24^ | . | 40 | . |
| 08-060 | FPAH | M | 36 | EUR | *BMPR2* | NM_001204.6 | c.1549del | p.(Thr517Glnfs*47) | frameshift | No | . | 35 | . |
| 22-060 | IPAH | F | 41 | EUR | *BMPR2* | NM_001204.6 | c.1744A>T | p.(Lys582*) | stopgain | No | . | 42 | . |
| 02-058 | IPAH | F | 68 | AMR | *BMPR2* | NM_001204.6 | c.1748dup | p.(Asn583Lysfs*6) | frameshift | No | . | 35 | . |
| 26-015 | APAH-unspecified | F | 51 | EUR | *BMPR2* | NM_001204.6 | c.1939_1940del | p.(Gln647Valfs*27) | frameshift | No | . | 34 | . |
| 26-023 | IPAH | F | 44 | EUR | *BMPR2* | NM_001204.6 | c.1958del | p.(Pro653Leufs*6) | frameshift | ^2, 13, 38^ | . | 29 | . |
| 13-079 | IPAH | F | 54 | EUR | *BMPR2* | NM_001204.6 | c.1981G>T | p.(Glu661*) | stopgain | ^2^ | . | 43 | . |
| 22-015 | IPAH | F | 49 | AFR | *BMPR2* | NM_001204.6 | c.2073dup | p.(Gln692Thrfs*12) | frameshift | no | . | 33 | . |
| 13-050 | APAH-HIV | F | 39 | AFR | *BMPR2* | NM_001204.6 | c.2140G>T | p.(Ala714Ser) | D-Mis | no | 9.08E-05 | 24 | 0.59 |
| 08-027 | IPAH | F | 30 | AFR | *BMPR2* | NM_001204.6 | c.2146G>T | p.(Glu716*) | stopgain | no | . | 45 | . |
| 04-001 | IPAH | F | 36 | EUR | *BMPR2* | NM_001204.6 | c.2202del | p.(Pro735Leufs*26) | frameshift | no | . | 31 | . |
| 12-152 | FPAH | F | 40 | EUR | *BMPR2* | NM_001204.6 | c.2216del | p.(Pro739Leufs*22) | frameshift | no | . | 35 | . |
| 07-086 | IPAH | F | 47 | EUR | *BMPR2* | NM_001204.6 | c.2268del | p.(Ser757Valfs*4) | frameshift | no | . | 35 | . |
| 06-008 | APAH-CHD | M | 2 | EUR | *BMPR2* | NM_001204.6 | c.2353G>A | p.(Glu785Lys) | D-Mis | ^19^ | 8.24E-06 | 32 | 0.59 |
| 26-033 | IPAH | M | 66 | EUR | *BMPR2* | NM_001204.6 | c.2396dup | p.(His800Serfs*13) | frameshift | no | . | 34 | . |
| 03-042 | FPAH | M | 54 | EUR | *BMPR2* | NM_001204.6 | c.2410_2413del | p.(Val804Profs*2) | frameshift | no | . | 35 | . |
| 08-062 | FPAH | F | 30 | EUR | *BMPR2* | NM_001204.6 | c.2450_2451del | p.(Asn817Ilefs*25) | frameshift | no | . | 35 | . |
| 08-034 | FPAH | M | 13 | EUR | *BMPR2* | NM_001204.6 | c.2450_2451del | p.(Asn817Ilefs*25) | frameshift | no | . | 35 | . |
| 08-035 | FPAH | F | 46 | EUR | *BMPR2* | NM_001204.6 | c.2450_2451del | p.(Asn817Ilefs*25) | frameshift | no | . | 35 | . |
| 02-011 | DTOX | F | 55 | EUR | *BMPR2* | NM_001204.6 | c.2457_2464del | p.(Ala820Asnfs*20) | frameshift | no | . | 35 | . |
| 12-048 | FPAH | F | 29 | EUR | *BMPR2* | NM_001204.6 | c.2580del | p.(Asn861Ilefs*11) | frameshift | no | . | 34 | . |
| 10-083 | FPAH | F | 20 | EUR | *BMPR2* | NM_001204.6 | c.2617C>T | p.(Arg873*) | stopgain | ^2, 8, 18^ ^20, 24, 26, 28, 35, 36^ | . | 44 | . |
| 13-059 | IPAH | F | 26 | EUR | *BMPR2* | NM_001204.6 | c.2617C>T | p.(Arg873*) | stopgain | ^2, 8, 18^ ^20, 24, 26, 28, 35, 36^ | . | 44 | . |
| 02-099 | DTOX | F | 38 | AMR | *BMPR2* | NM_001204.6 | c.2651_2652insAT | p.(Asp885Trpfs*12) | frameshift | no | . | 34 | . |
| 02-148 | IPAH | F | 28 | AFR | *BMPR2* | NM_001204.6 | c.2695C>T | p.(Arg899*) | stopgain | ^2, 8, 14, 16, 17, 21, 24, 26^ | . | 39 | . |
| 08-019 | IPAH | F | 35 | EUR | *BMPR2* | NM_001204.6 | c.2695C>T | p.(Arg899*) | stopgain | ^2, 8, 21, 24^ ^16^ ^14, 17, 26^ | . | 39 | . |
| 14-033 | FPAH | F | 40 | EUR | *BMPR2* | NM_001204.6 | c.2695C>T | p.(Arg899*) | stopgain | ^2, 8, 14, 16, 17, 21, 24, 26^ | . | 39 | . |
| 28-008 | IPAH | F | 41 | EUR | *BMPR2* | NM_001204.6 | c.2695C>T | p.(Arg899*) | stopgain | ^2, 8, 14, 16, 17, 21, 24, 26^ | . | 39 | . |
| 03-051 | IPAH | F | 41 | EUR | *BMPR2* | NM_001204.6 | c.2730T>A | p.(Cys910*) | stopgain | ^2^ | . | 36 | . |
| 14-044 | IPAH | F | 53 | AMR | *BMPR2* | NM_001204.6 | c.2952del | p.(Trp984Cysfs*50) | frameshift | no | . | 35 | . |
| 10-021 | IPAH | F | 55 | EUR | *BMPR2* | . | exon1 del | . | exon deletion | ^23, 39^ | N/A | . | . |
| 11-010 | IPAH | F | 26 | EUR | *BMPR2* | . | exon 1 del | . | exon deletion | ^23, 39^ | N/A | . | . |
| 03-029 | FPAH | M | 33 | EUR | *BMPR2* | . | exon 2-3 del | . | exon deletion | ^40^ | N/A | . | . |
| 04-019 | IPAH | F | 44 | EUR | *BMPR2* | . | exon 2-3 del | . | exon deletion | ^40^ | N/A | . | . |
| 04-075 | IPAH | F | 22 | EUR | *BMPR2* | . | exon 2-3 del | . | exon deletion | ^40^ | N/A | . | . |
| 10-010 | IPAH | F | 21 | EUR | *BMPR2* | . | exon 2-3 del | . | exon deletion | ^40^ | N/A | . | . |
| 12-207 | IPAH | F | 35 | EUR | *BMPR2* | . | exon 2-3 del | . | exon deletion | ^40^ | N/A | . | . |
| 04-060 | IPAH | M | 55 | EUR | *BMPR2* | . | exon 3 del | . | exon deletion | ^41^ | N/A | . | . |
| 08-023 | FPAH | F | 47 | EUR | *BMPR2* | . | exon 3 del | . | exon deletion | ^41^ | N/A | . | . |
| 08-073 | FPAH | M | 58 | EUR | *BMPR2* | . | exon 3 del | . | exon deletion | ^41^ | N/A | . | . |
| 08-114 | FPAH | F | 34 | EUR | *BMPR2* | . | exon 3 del | . | exon deletion | ^41^ | N/A | . | . |
| 12-013 | FPAH | F | 59 | EUR | *BMPR2* | . | exon 3 del | . | exon deletion | ^41^ | N/A | . | . |
| 10-095 | FPAH | M | 69 | EUR | *BMPR2* | . | exon 4 del | . | exon deletion | no | N/A | . | . |
| 22-017 | FPAH | F | 45 | EUR | *BMPR2* | . | exon 4 del | . | exon deletion | no | N/A | . | . |
| 07-006 | IPAH | F | 42 | EUR | *BMPR2* | . | exon 4-5 del | . | exon deletion | ^41, 42^ | N/A | . | . |
| 05-152 | IPAH | F | 36 | AMR | *BMPR2* | . | exon 4-7 del | . | exon deletion | no | N/A | . | . |
| 10-002 | FPAH | M | 26 | EUR | *BMPR2* | . | exon 4-7 del | . | exon deletion | no | N/A | . | . |
| 14-039 | FPAH | M | 60 | EUR | *BMPR2* | . | exon 4-7 del | . | exon deletion | no | N/A | . | . |
| 05-109 | IPAH | F | 22 | AMR | *BMPR2* | . | exon 4-8 del | . | exon deletion | no | N/A | . | . |
| 26-038 | IPAH | F | 64 | EUR | *BMPR2* | . | exon 4-9 del | . | exon deletion | no | N/A | . | . |
| 08-026 | IPAH | F | 56 | EUR | *BMPR2* | . | exon 6 del | . | exon deletion | ^16^ | N/A | . | . |
| 02-139 | FPAH | F | 25 | AMR | *BMPR2* | . | exon 8-9 del | . | exon deletion | ^39^ | N/A | . | . |
| 08-096 | IPAH | F | 32 | EUR | *BMPR2* | . | exon 11-12 del | . | exon deletion | ^23, 39^ | N/A | . | . |
| 17-015 | APAH-Portopulm | M | 56 | EAS | *CAV1* | NM_001172896.1 | c.-13_-10del | p.(=) | splicing | no | 1.65E-05 | 24 | . |
| 17-012 | IPAH | F | 33 | AFR | *CAV1* | NM_001172896.1 | c.191C>T | p.(Thr64Met) | D-Mis | no | 2.49E-05 | 31 | 0.92 |
| 04-038 | IPAH | F | 29 | EUR | *CAV1* | NM_001172896.1 | c.209G>A | p.(Arg70His) | D-Mis | no | 3.31E-05 | 32 | 0.96 |
| 17-064 | APAH-CHD | F | 54 | EUR | *CAV1* | NM_001172896.1 | c.274del | p.(Ser92Leufs*16) | frameshift | no | . | 35 | . |
| 08-041 | FPAH | F | 5 | EUR | *CAV1* | NM_001172896.1 | c.381del | p.(Leu128Serfs*22) | frameshift | no | . | 32 | . |
| 08-086 | FPAH | F | 41 | EUR | *CAV1* | NM_001172896.1 | c.381del | p.(Leu128Serfs*22) | frameshift | no | . | 32 | . |
| 08-087 | FPAH | M | 67 | EUR | *CAV1* | NM_001172896.1 | c.381del | p.(Leu128Serfs*22) | frameshift | no | . | 32 | . |
| 11-024 | APAH-CTD | F | 69 | EUR | *CAV1* | NM_001172896.1 | c.407T>C | p.(Phe136Ser) | D-Mis | no | 4.13E-05 | 27 | 0.90 |
| 13-062 | APAH-Portopulm | M | 59 | EUR | *CAV1* | NM_001172896.1 | c.407T>C | p.(Phe136Ser) | D-Mis | no | 4.13E-05 | 27 | 0.90 |
| 16-008 | IPAH | F | 45 | AFR | *CAV1* | NM_001172896.1 | c.418C>T | p.(Arg140Cys) | D-Mis | no | 8.27E-06 | 33 | 0.82 |
| 12-014** | APAH-CTD | F | 78 | EUR | *EIF2AK4* | NM_001013703.3 | c.220G>A | p.(Asp74Asn) | D-Mis | no | 1.66E-05 | 32 | 0.17 |
| 12-014** | APAH-CTD | F | 78 | EUR | *EIF2AK4* | NM_001013703.3 | c.3111G>C | p.(Gln1037His) | D-Mis | no | 8.33E-06 | 25 | 0.51 |
| 02-030** | APAH-CTD | F | 42 | AFR | *EIF2AK4* | NM_001013703.3 | c.650C>G | p.(Pro217Arg) | D-Mis | no | 2.00E-04 | 23 | 0.03 |
| 02-030** | APAH-CTD | F | 42 | AFR | *EIF2AK4* | NM_001013703.3 | c.1265T>G | p.(Val422Gly) | D-Mis | no | 3.00E-04 | 29 | 0.92 |
| 10-091** | FPAH-PVOD | M | 36 | EUR | *EIF2AK4* | NM_001013703.3 | c.1153dup | p.(Val385Glyfs*30) | frameshift | ^43^ | 3.31E-05 | 32 | . |
| 12-064** | PVOD | F | 48 | EUR | *EIF2AK4* | NM_001013703.3 | c.2141C>A | p.(Ser714*) | stopgain | no | . | 42 | . |
| 10-091** | FPAH-PVOD | M | 36 | EUR | *EIF2AK4* | NM_001013703.3 | c.3766C>T | p.(Arg1256*) | stopgain | ^2, 6, 43^ | 1.00E-04 | 49 | . |
| 21-036** | IPAH | F | 33 | EUR | *EIF2AK4* | NM_001013703.3 | c.4593del | p.(Ile1533Leufs*2) | frameshift | no | . | 27 | . |
| 12-009 | APAH-HHT | F | 78 | EUR | *ENG* | NM_000118.3 | c.277C>T | p.(Arg93*) | stopgain | ^1, 6, 44^ | . | 37 | . |
| 06-114 | APAH-HHT | F | 38 | AMR | *ENG* | NM_000118.3 | c.715dup | p.(Glu239Glyfs*95) | frameshift | ^44^ | . | 28 | . |
| 03-023 | APAH-CTD | F | 69 | EUR | *ENG* | NM_000118.3 | c.1361T>G | p.(Leu454Arg) | D-Mis | no | . | 26 | 0.61 |
| 17-051 | APAH-HIV | M | 57 | AFR | *ENG* | NM_000118.3 | c.1415A>T | p.(Gln472Leu) | D-Mis | no | . | 25 | 0.60 |
| 22-046 | APAH-CTD | F | 53 | EUR | *ENG* | NM_000118.3 | c.1585C>T | p.(Arg529Cys) | D-Mis | no | 1.65E-05 | 29 | 0.64 |
| 38-002 | APAH-CTD | M | 68 | AFR | *ENG* | NM_000118.3 | c.*202_*210dup | p.(=) | splicing | no | 8.03E-05 | 28 | . |
| 25-007 | FPAH | M | 6 | EUR | *KCNK3* | NM_002246.2 | c.544G>A | p.(Glu182Lys) | D-Mis | ^6, 45^ | . | 32 | 0.59 |
| 15-043 | IPAH | F | 5 | EUR | *KCNK3* | NM_002246.2 | c.646_651dup | p.(Thr216_Gln217dup) | in-frame | ^6^ | . | 13 | . |
| 05-138 | APAH-CTD | F | 49 | EUR | *KCNK3* | NM_002246.2 | c.1075_1076del | p.(Arg359Thrfs*278) | frameshift | no | . | 35 | . |
| 14-047 | IPAH | M | 62 | EUR | *SMAD4* | NM_005359.5 | c.466_468del | p.(Met157del) | in-frame | no | . | 21 | . |
| 17-009 | IPAH | F | 52 | EUR | *SMAD4* | NM_005359.5 | c.1460C>T | p.(Ala487Val) | D-Mis | no | . | 24 | 0.67 |
| 23-009 | APAH-CTD | F | 45 | EUR | *SMAD9* | NM_001127217.2 | c.138_141del | p.(Lys47Argfs*43) | frameshift | no | 1.65E-05 | 27 |  |
| 04-092 | IPAH | M | 58 | AFR | *SMAD9* | NM_001127217.2 | c.146_148del | p.(Lys49_Gly50delinsArg) | in-frame | no | 1.65E-05 | 14 |  |
| 06-124 | APAH-CHD | F | 1 | Other | *SMAD9* | NM_001127217.2 | c.204C>A | p.(Cys68*) | stopgain | no | . | 32 | . |
| 11-007 | APAH-HIV | F | 44 | AFR | *SMAD9* | NM_001127217.2 | c.430G>T | p.(Val144Leu) | D-Mis | no | 5.78E-05 | 28 | 0.70 |
| 02-050 | APAH-CTD | F | 47 | AMR | *SMAD9* | NM_001127217.2 | c.438A>C | p.(Arg146Ser) | D-Mis | no | . | 25 | 0.81 |
| 02-022 | IPAH | F | 57 | AMR | *SMAD9* | NM_001127217.2 | c.804G>C | p.(Glu268Asp) | D-Mis | no | . | 23 | 0.63 |
| 23-004 | APAH-CHD | F | 42 | EUR | *SMAD9* | NM_001127217.2 | c.851G>T | p.(Arg284Leu) | D-Mis | no | 1.65E-05 | 34 | 0.92 |
| 18-070 | APAH-CHD | F | 65 | EAS | SMAD9 | NM_001127217.2 | c.767C>A | p.(Ser256Leu) | D-Mis | no | . | 29 | 0.82 |
| 15-051* | IPAH | F | 4 | EUR | *SMAD9* | NM_001127217.2 | c.880C>T | p.(Arg294*) | stopgain | no | 8.24E-06 | 40 | . |
| 28-062 | APAH-CTD | F | 45 | EUR | *SMAD9* | NM_001127217.2 | c.971C>T | p.(Thr324Met) | D-Mis | no | 8.24E-05 | 28 | 0.79 |
| 26-019 | APAH-CTD | F | 46 | AMR | *SMAD9* | NM_001127217.2 | c.995_997del | p.(Ile332_Gly333delinsArg) | frameshift | no | . | 35 | . |
| 16-040 | IPAH | F | 59 | EUR | *SMAD9* | NM_001127217.2 | c.1260G>C | p.(Lys420Asn) | D-Mis | no | . | 31 | 0.86 |
| 22-006 | FPAH | F | 39 | AMR | *SMAD9* | NM_001127217.2 | c.1321C>T | p.(His441Tyr) | D-Mis | no | . | 29 | 0.92 |
| 15-078 | DTOX | F | 2 | Dominican | *TBX4* | NM_018488.3 | c.146del | p.(Gly49Aspfs*39) | frameshift | no | . | 26 | . |
| 06-061 | IPAH | M | 3 | EUR | *TBX4* | NM_018488.3 | c.150del | p.(Ala52Profs*36) | frameshift | ^6^ | . | 25 | . |
| 05-184 | DTOX | F | 45 | EUR | *TBX4* | NM_018488.3 | c.179_180dup | p.(Glu61Argfs*28) | frameshift | no | . | 28 | . |
| 02-191 | IPAH | F | 5 | AMR | *TBX4* | NM_018488.3 | c.210dup | p.(Leu71Alafs*3) | frameshift | no | . | 35 | . |
| 08-001 | IPAH | F | 40 | EUR | *TBX4* | NM_018488.3 | c.293C>T | p.(Pro98Leu) | D-Mis | no | . | 34 | 0.97 |
| 02-071* | FPAH | F | 40 | EUR | *TBX4* | NM_018488.3 | c.299A>G | p.(Tyr100Cys) | D-Mis | no | . | 27 | 0.73 |
| 15-013 | IPAH | M | 13 | EUR | *TBX4* | NM_018488.3 | c.316G>A | p.(Gly106Ser) | D-Mis | no | 4.12E-05 | 31 | 0.96 |
| 06-009 | IPAH | F | 14 | AFR | *TBX4* | NM_018488.3 | c.380A>C | p.(Tyr127Ser) | D-Mis | ^6^ | . | 28 | 0.93 |
| 12-080 | APAH-CTD | F | 29 | AMR | *TBX4* | NM_018488.3 | c.455C>T | p.(Pro152Leu) | D-Mis | no | 1.65E-05 | 34 | 0.95 |
| 10-024 | DTOX | F | 71 | EUR | *TBX4* | NM_018488.3 | c.529C>T | p.(His177Tyr) | D-Mis | no | . | 20 | 0.57 |
| 12-145 | IPAH | M | 70 | EUR | *TBX4* | NM_018488.3 | c.561_570dup | p.(Lys191Leufs*13) | frameshift | no | . | 35 | . |
| 02-159 | APAH-CTD | F | 15 | EUR | *TBX4* | NM_018488.3 | c.571_576del | p.(Lys191_Tyr192del) | in-frame | no | . | 23 | . |
| 15-046 | APAH-CHD | F | 0 | EUR | *TBX4* | NM_018488.3 | c.702+1G>A | p.(=) | splicing | ^6, 19^ | . | 27 | . |
| 19-038 | APAH-CHD | F | 59 | EUR | *TBX4* | NM_018488.3 | c.782G>A | p.(Arg261Gln) | D-Mis | no | 8.24E-06 | 34 | 0.66 |
| 24-005 | FPAH | M | 15 | Dominican | *TBX4* | NM_018488.3 | c.789del | p.(Ser264Alafs*6) | frameshift | no | . | 33 | . |
| 10-109 | IPAH | F | 57 | AMR | *TBX4* | NM_018488.3 | c.809T>G | p.(Ile270Ser) | D-Mis | no | . | 27 | 0.62 |
| 05-151 | IPAH | M | 63 | EUR | *TBX4* | NM_018488.3 | c.847dup | p.(Gln283Profs*103) | frameshift | ^6^ | . | 24 | . |
| 15-062 | IPAH | F | 5 | EUR | *TBX4* | NM_018488.3 | c.847dup | p.(Gln283Profs*103) | frameshift | ^6^ | . | 24 | . |
| 01-008 | IPAH | F | 7 | EUR | *TBX4* | NM_018488.3 | c.985G>T | p.(Asp329Tyr) | D-Mis | no | . | 30 | 0.61 |
| 12-102 | FPAH | M | 55 | EUR | *TBX4* | NM_018488.3 | c.1021+1G>A | p.(=) | splicing | no | . | 27 | . |
| 06-052 | APAH-CHD | M | 1 | EUR | *TBX4* | NM_018488.3 | c.1112del | p.(Pro371Leufs*8) | frameshift | ^19^ | . | 33 | . |
| 12-045 | IPAH | F | 52 | EUR | *TBX4* | NM_018488.3 | c.1119C>A | p.(Tyr373*) | stopgain | no | . | 36 | . |
| 02-168 | IPAH | M | 17 | EAS | *TBX4* | NM_018488.3 | c.1417_1420dup | p.(Tyr474Cysfs*30) | frameshift | no | . | 21 | . |

Rare, predicted deleterious variants defined as MAF ≤1.00E-04 and LGD (stopgain, frameshift, splicing) or missense with REVEL score >0.5 (D-Mis). For biallelic *EIF2AK4* variants, MAF ≤1.00E-02 and LGD or missense with CADD ≥20. CADD scores for all variants are provided as a reference.

*Patients 02-071, 02-100, 12-015 and 15-051 carried variants in more than one risk gene.

**Patients 12-064 and 21-036 are homozygous for the given EIF2AK4 mutations. Patients 12-014, 02-030 and 10-091 are compound heterozygotes.

Abbreviations: dx, diagnosis; HHT, hereditary hemorrhagic telangiectasia; MAF, minor allele frequency; NF, neurofibromatosis; EUR, European; AMR, admixed American (Hispanic); SAS, southeast Asian; EAS, East Asian; AFR, African.

**Supplementary Table 4. Rare, predicted deleterious variants in recently-reported PAH risk genes among 2572 PAH cases. Patients are heterozygous for the indicated variant.**

| **Patient ID** | **PAH subclass** | **Gender** | **Age at dx** | **Ancestry** | **Gene** | **Transcript** | **Nucleotide change** | **Amino acid change** | **Variant type** | **Previously reported?** | **MAF (ExAC)** | **CADD** | **REVEL** |
| --- | --- | --- | --- | --- | --- | --- | --- | --- | --- | --- | --- | --- | --- |
| 18-002 | IPAH | F | 34 | EAS | *ABCC8* | NM_001351295.1 | c.307C>T | p.(His103Tyr) | D-Mis | rs751209734 | 4.95E-05 | 23 | 0.66 |
| 05-145 | APAH-CHD | F | 42 | AMR | *ABCC8* | NM_001351295.1 | c.313C>T | p.(His105Tyr) | D-Mis | no | 1.65E-05 | 27 | 0.78 |
| 12-207 | IPAH | F | 43 | EUR | *ABCC8* | NM_001351295.1 | c.375C>G | p.(His125Gln) | D-Mis | rs60637558 | 8.25E-05 | 26 | 0.89 |
| 05-166 | APAH-CTD | F | 31 | SAS | *ABCC8* | NM_001351295.1 | c.503G>A | p.(Arg168His) | D-Mis | no | . | 30 | 0.88 |
| 02-060 | IPAH | F | 34 | AMR | *ABCC8* | NM_001351295.1 | c.610G>T | p.(Val204Leu) | D-Mis | no | . | 20 | 0.54 |
| 22-023 | IPAH | F | 33 | EUR | *ABCC8* | NM_001351295.1 | c.647G>A | p.(Arg216His) | D-Mis | rs199702708 | 2.47E-05 | 24 | 0.60 |
| 08-043 | IPAH | M | 57 | EUR | *ABCC8* | NM_001351295.1 | c.890G>T | p.(Arg297Met) | D-Mis | no | 8.30E-06 | 26 | 0.82 |
| 13-078 | APAH-CTD | F | 41 | AFR | *ABCC8* | NM_001351295.1 | c.1024G>A | p.(Gly342Arg) | D-Mis | no | 3.31E-05 | 23 | 0.63 |
| 02-179 | APAH-CTD | F | 50 | EUR | *ABCC8* | NM_001351295.1 | c.1198A>G | p.(Met400Val) | D-Mis | no | . | 22 | 0.55 |
| 05-033 | IPAH | F | 63 | EUR | *ABCC8* | NM_001351295.1 | c.1198A>G | p.(Met400Val) | D-Mis | no | . | 22 | 0.55 |
| 07-025 | APAH-CTD | F | 64 | AFR | *ABCC8* | NM_001351295.1 | c.1270G>A | p.(Asp424Asn) | D-Mis | rs577545383 | 2.47E-05 | 34 | 0.96 |
| 13-034 | IPAH | F | 73 | EUR | *ABCC8* | NM_001351295.1 | c.1484G>A | p.(Arg495Gln) | D-Mis | no | . | 35 | 0.87 |
| 14-006 | APAH-HIV | M | 44 | EUR | *ABCC8* | NM_001351295.1 | c.1531C>G | p.(Leu511Val) | D-Mis | no | 8.25E-06 | 27 | 0.82 |
| 06-043 | APAH-CHD | F | 44 | AMR | *ABCC8* | NM_001351295.1 | c.1676T>C | p.(Phe559Ser) | D-Mis | no | . | 32 | 0.95 |
| 16-054 | IPAH | F | 44 | AFR | *ABCC8* | NM_001351295.1 | c.1919C>A | p.(Ala640Glu) | D-Mis | no | 8.26E-06 | 16 | 0.54 |
| 17-018 | APAH-CTD | F | 48 | EUR | *ABCC8* | NM_001351295.1 | c.2003T>C | p.(Val668Ala) | D-Mis | no | . | 23 | 0.57 |
| 18-025 | IPAH | F | 33 | EAS | *ABCC8* | NM_001351295.1 | c.2008C>T | p.(Arg670Cys) | D-Mis | no | 8.31E-06 | 34 | 0.73 |
| 19-051 | IPAH | F | 29 | AMR | *ABCC8* | NM_001351295.1 | c.2008C>T | p.(Arg670Cys) | D-Mis | no | 8.31E-06 | 34 | 0.73 |
| 11-009 | APAH-CTD | F | 76 | EUR | *ABCC8* | NM_001351295.1 | c.2218G>A | p.(Gly740Ser) | D-Mis | no | 1.66E-05 | 35 | 0.96 |
| 16-036 | APAH-CTD | F | 64 | EUR | *ABCC8* | NM_001351295.1 | c.2228C>T | p.(Ser743Leu) | D-Mis | no | . | 34 | 0.95 |
| 06-018 | IPAH | M | 18 | EUR | *ABCC8* | NM_001351295.1 | c.2230C>T | p.(Leu744Phe) | D-Mis | no | 8.26E-06 | 29 | 0.90 |
| 31-006 | APAH-CTD | F | 50 | AFR | *ABCC8* | NM_001351295.1 | c.2439T>A | p.(Ser813Arg) | D-Mis | no | . | 17 | 0.59 |
| 04-056 | IPAH | F | 41 | EUR | *ABCC8* | NM_001351295.1 | c.2474A>G | p.(Glu825Gly) | D-Mis | no | . | 23 | 0.56 |
| 15-025 | APAH-CHD | F | 2 | EUR | *ABCC8* | NM_001351295.1 | c.2488C>A | p.(Gln830Lys) | D-Mis | no | 4.12E-05 | 20 | 0.60 |
| 07-055 | IPAH | F | 67 | EUR | *ABCC8* | NM_001351295.1 | c.2488C>A | p.(Gln830Lys) | D-Mis | no | 4.12E-05 | 20 | 0.60 |
| 06-091 | APAH-CHD | F | 15 | AFR | *ABCC8* | NM_001351295.1 | c.3410C>T | p.(Thr1137Met) | D-Mis | no | 8.25E-06 | 34 | 0.61 |
| 03-076 | APAH-CTD | F | 64 | EUR | *ABCC8* | NM_001351295.1 | c.3571C>A | p.(Pro1191Thr) | D-Mis | no | . | 27 | 0.84 |
| 20-035 | IPAH | F | 35 | AFR | *ABCC8* | NM_001351295.1 | c.3644A>T | p.(Asp1215Val) | D-Mis | no | 2.47E-05 | 29 | 0.94 |
| 06-052 | IPAH | M | 1 | EUR | *ABCC8* | NM_001351295.1 | c.4004G>A | p.(Arg1335His) | D-Mis | ^46^ | 2.48E-05 | 34 | 0.93 |
| 12-063 | APAH-CTD | F | 30 | EUR | *ATP13A3* | NM_024524.3 | c.158_159del; 163_167del | p.(Trp53Serfs*12) | frameshift | no | . | 27 | . |
| 07-091 | IPAH | F | 40 | EUR | *ATP13A3* | NM_024524.3 | c.201_202del | p.(Cys67*) | stopgain | no | . | 25 | . |
| 03-012 | APAH-CHD | M | 30 | EUR | *ATP13A3* | NM_024524.3 | c.1222A>G | p.(Arg408Gly) | D-Mis | no | . | 26 | 0.72 |
| 04-025 | IPAH | F | 66 | EUR | *ATP13A3* | NM_024524.3 | c.2189_2205del | p.(Thr730Argfs*4) | frameshift | no | . | 35 | . |
| 14-040 | IPAH | F | 56 | SAS | *ATP13A3* | NM_024524.3 | c.2228G>T | p.(Arg743Leu) | D-Mis | no | . | 35 | 0.75 |
| 09-043 | IPAH | F | 45 | EUR | *ATP13A3* | NM_024524.3 | c.2549dup | p.(Met850Ilefs*13) | frameshift | no | . | 35 | . |
| 06-014 | FPAH | M | 35 | EUR | *ATP13A3* | NM_024524.3 | c.2996C>T | p.(Ser999Leu) | D-Mis | no | 8.30E-06 | 29 | 0.74 |
| 18-055 | IPAH | M | 53 | AMR | *GDF2* | NM_016204.3 | c.76C>T | p.(Gln26*) | stopgain | ^47^ | . | 36 | . |
| 26-003 | IPAH | F | 39 | EUR | *GDF2* | NM_016204.3 | c.143dup | p.(Pro49Alafs*2) | frameshift | no | . | 23 | . |
| 18-029 | IPAH | F | 22 | SAS | *GDF2* | NM_016204.3 | c.230C>T | p.(Ser77Leu) | D-Mis | no | . | 22 | . |
| 34-016 | APAH-CTD | F | 67 | AMR | *GDF2* | NM_016204.3 | c.254C>T | p.(Pro85Leu) | D-Mis | no | . | 24 | . |
| 02-116 | IPAH | F | 55 | EUR | *GDF2* | NM_016204.3 | c.328C>T | p.(Arg110Trp) | D-Mis | ^8^ | . | 31 | . |
| 05-003 | IPAH | M | 48 | EUR | *GDF2* | NM_016204.3 | c.329G>A | p.(Arg110Gln) | D-Mis | no | . | 34 | . |
| 10-056 | IPAH | M | 26 | AMR | *GDF2* | NM_016204.3 | c.502G>T | p.(Gly168*) | stopgain | no | . | 37 | . |
| 28-107 | IPAH | F | 39 | AMR | *GDF2* | NM_016204.3 | c.530A>T | p.(Asp177Val) | D-Mis | no | . | 26 | . |
| 05-109 | IPAH | F | 22 | AMR | *GDF2* | NM_016204.3 | c.607G>C | p.(Glu203Gln) | D-Mis | no | . | 24 | . |
| 24-022 | IPAH | M | 9 | EAS | *GDF2* | NM_016204.3 | c.646C>T | p.(Arg216Trp) | D-Mis | no | . | 24 | . |
| 13-058 | APAH-HIV | M | 70 | EUR | *GDF2* | NM_016204.3 | c.751del | p.(Leu251Cysfs*22) | frameshift | no | . | 24 | . |
| 12-083 | IPAH | F | 46 | EUR | *GDF2* | NM_016204.3 | c.751del | p.(Leu251Cysfs*22) | frameshift | no | . | 24 | . |
| 03-122 | IPAH | F | 45 | EUR | *GDF2* | NM_016204.3 | c.857dup | p.(Leu287Alafs*11) | frameshift | no | . | 24 | . |
| 27-008 | IPAH | F | 41 | EUR | *GDF2* | NM_016204.3 | c.857dup | p.(Leu287Alafs*11) | frameshift | no | . | 24 | . |
| 31-021 | APAH-HIV | F | 52 | AFR | *GDF2* | NM_016204.3 | c.997C>T | p.(Arg333Trp) | D-Mis | no | 1.65E-05 | 22 | . |
| 18-079 | IPAH | F | 31 | AMR | *GDF2* | NM_016204.3 | c.997C>T | p.(Arg333Trp) | D-Mis | no | 1.65E-05 | 22 | . |
| 02-039 | IPAH | F | 45 | AMR | *GDF2* | NM_016204.3 | c.1011G>T | p.(Glu337Asp) | D-Mis | no | . | 23 | . |
| 02-039 | IPAH | F | 45 | AMR | *GDF2* | NM_016204.3 | c.1012G>T | p.(Asp338Tyr) | D-Mis | no | . | 28 | . |
| 29-045 | FPAH | F | 64 | EUR | *GDF2* | NM_016204.3 | c.1023G>C | p.(Trp341Cys) | D-Mis | no | . | 35 | . |
| 06-014 | FPAH | M | 35 | EUR | *GDF2* | NM_016204.3 | c.1042C>A | p.(Pro348Thr) | D-Mis | no | . | 25 | . |
| 27-002 | IPAH | M | 59 | EUR | *GDF2* | NM_016204.3 | c.1063G>C | p.(Glu355Gln) | D-Mis | no | . | 25 | . |
| 09-073 | IPAH | F | 18 | AFR | *GDF2* | NM_016204.3 | c.1103T>C | p.(Val368Ala) | D-Mis | no | . | 24 | . |
| 02-173 | APAH-CTD | F | 51 | AFR | *GDF2* | NM_016204.3 | c.1135del | p.(Leu379fs) | frameshift | no | . | . | . |
| 17-089 | APAH-HIV | F | 43 | AFR | *GDF2* | NM_016204.3 | c.1135del | p.(Leu379fs) | frameshift | no | . | . | . |
| 17-017 | IPAH | F | 33 | EUR | *GDF2* | NM_016204.3 | c.1186A>G | p.(Thr396Ala) | D-Mis | no | . | 29 | . |
| 05-156 | IPAH | F | 40 | EAS | *GDF2* | NM_016204.3 | c.1259G>C | p.(Gly420Ala) | D-Mis | no | . | 27 | . |
| 09-046 | IPAH | F | 24 | EUR | *GDF2* | NM_016204.3 | c.1267G>A | p.(Val423Met) | D-Mis | no | . | 29 | . |
| 02-129 | IPAH | F | 25 | EUR | *GDF2* | NM_016204.3 | c.1282T>C | p.(Cys428Arg) | D-Mis | no | . | 31 | . |
| 05-174 | APAH-CTD | F | 56 | AMR | *KCNA5* | NM_002234.3 | c.1A>C | (p.?) | D-Mis | no | . | 23 | 0.55 |
| 28-020 | IPAH | F | 21 | EUR | *KCNA5* | NM_002234.3 | c.660T>G | p.(Ile220Met) | D-Mis | no | . | 16 | 0.5 |
| 12-050 | FPAH | F | 36 | EUR | *KCNA5* | NM_002234.3 | c.670del | p.(Glu224Argfs*134) | frameshift | no | . | 21 | . |
| 12-130 | FPAH | F | 28 | EUR | *KCNA5* | NM_002234.3 | c.670del | p.(Glu224Argfs*134) | frameshift | no | . | 21 | . |
| 12-140 | FPAH | F | 59 | EUR | *KCNA5* | NM_002234.3 | c.670del | p.(Glu224Argfs*134) | frameshift | no | . | 21 | . |
| 11-060 | APAH-CTD | M | 62 | EUR | *KCNA5* | NM_002234.3 | c.964G>C | p.(Asp322His) | D-Mis | no | 9.15E-05 | 24 | 0.83 |
| 20-028 | APAH-CHD | F | 26 | EUR | *KCNA5* | NM_002234.3 | c.964G>C | p.(Asp322His) | D-Mis | no | 9.15E-05 | 24 | 0.83 |
| 17-098 | APAH-APAH | F | 73 | AFR | *KCNA5* | NM_002234.3 | c.1043G>A | p.(Ser348Asn) | D-Mis | no | 8.25E-06 | 25 | 0.56 |
| 04-081 | APAH-CTD | F | 43 | EUR | *KCNA5* | NM_002234.3 | c.1243C>T | p.(Arg415Cys) | D-Mis | no | . | 32 | 0.84 |
| 21-048 | IPAH | F | 75 | EUR | *KCNA5* | NM_002234.3 | c.1327A>G | p.(Ile443Val) | D-Mis | no | 5.78E-05 | 25 | 0.67 |
| 05-179 | APAH-CTD | F | 50 | EUR | *KCNA5* | NM_002234.3 | c.1472T>C | p.(Val491Ala) | D-Mis | no | 4.94E-05 | 23 | 0.55 |
| 18-014 | DTOX | M | 45 | EUR | *KCNA5* | NM_002234.3 | c.1564T>C | p.(Phe522Leu) | D-Mis | no | . | 29 | 0.93 |
| 12-017 | APAH-CHD | F | 30 | EAS | *KCNA5* | NM_002234.3 | c.1727C>T | p.(Ala576Val) | D-Mis | no | 3.33E-05 | 13 | 0.54 |
| 07-020 | APAH-CHD | F | 53 | EUR | *SMAD1* | NM_005900.2 | c.469C>T | p.(Arg157Cys) | D-Mis | no | 8.25E-06 | 32 | 0.56 |
| 06-089 | APAH-CTD | F | 50 | EUR | *SMAD1* | NM_005900.2 | c.671C>A | p.(Pro224Gln) | D-Mis | no | . | 24 | 0.71 |
| 06-116 | APAH-CHD | F | 3 | EUR | *SOX17* | NM_022454.3 | c.226A>G | p.(Met76Val) | D-Mis | ^19^ | . | 26 | 0.97 |
| 29-021 | APAH-Portopulm | F | 57 | AMR | *SOX17* | NM_022454.3 | c.277C>A | p.(Leu93Met) | D-Mis | no | . | 26 | 0.63 |
| 24-002 | IPAH | F | 7 | EUR | *SOX17* | NM_022454.3 | c.365_366del | p.(Glu122Alafs*39) | frameshift | no | . | 34 | . |
| 28-150 | APAH-CHD | F | 31 | AMR | *SOX17* | NM_022454.3 | c.392A>G | p.(Asp131Gly) | D-Mis | ^19^ | . | 29 | 0.89 |
| 05-192 | IPAH | F | 40 | AMR | *SOX17* | NM_022454.3 | c.392A>G | p.(Asp131Gly) | D-Mis | ^19^ | . | 29 | 0.89 |
| 06-005 | IPAH | F | 5 | EUR | *SOX17* | NM_022454.3 | c.418C>T | p.(Arg140Trp) | D-Mis | no | . | 35 | 0.68 |
| 06-012 | IPAH | F | 5 | AMR | *SOX17* | NM_022454.3 | c.499_520del | p.(Leu167Trpfs*213) | frameshift | ^8^ | . | 34 | . |
| 08-008 | DTOX | M | 31 | EUR | *SOX17* | NM_022454.3 | c.788del | p.(Pro263Argfs*124) | frameshift | no | . | 10 | . |
| 07-059 | IPAH | F | 66 | EUR | *SOX17* | NM_022454.3 | c.1190C>T | p.(Ser397Leu) | D-Mis | no | . | 34 | 0.80 |
| 06-016 | IPAH | M | 19 | EUR | *SOX17* | NM_022454.3 | c.1224del | p.(Cys409Alafs*45) | frameshift | no | . | 33 | . |

Rare, predicted deleterious variants defined as MAF ≤1.00E-04 and LGD (stopgain, frameshift, splicing) or missense with REVEL score >0.5 (D-Mis). CADD scores are provided as a reference.

Abbreviations: dx, diagnosis; MAF, minor allele frequency; EUR, European; AMR, admixed American (Hispanic); SAS, southeast Asian; EAS, East Asian; AFR, African.

**Supplementary Table 3 and 4 References**

1. Richards-Yutz, J., Grant, K., Chao, E.C., Walther, S.E. & Ganguly, A. Update on molecular diagnosis of hereditary hemorrhagic telangiectasia. *Hum Genet* **128**, 61-77 (2010).

2. Machado, R.D. *et al.* Pulmonary Arterial Hypertension: A Current Perspective on Established and Emerging Molecular Genetic Defects. *Hum Mutat* **36**, 1113-1127 (2015).

3. Chen, Y.J. *et al.* Clinical and genetic characteristics of Chinese patients with hereditary haemorrhagic telangiectasia-associated pulmonary hypertension. *Eur J Clin Invest* **43**, 1016-1024 (2013).

4. Austin, E.D. & Loyd, J.E. Toward Precision Medicine in Pulmonary Arterial Hypertension. *Am J Respir Crit Care Med* **192**, 1272-1274 (2015).

5. Pfarr, N. *et al.* Hemodynamic and genetic analysis in children with idiopathic, heritable, and congenital heart disease associated pulmonary arterial hypertension. *Respir Res* **14**, 3 (2013).

6. Zhu, N. *et al.* Exome Sequencing in Children With Pulmonary Arterial Hypertension Demonstrates Differences Compared With Adults. *Circ Genom Precis Med* **11**, e001887 (2018).

7. Fujiwara, M. *et al.* Implications of mutations of activin receptor-like kinase 1 gene (ALK1) in addition to bone morphogenetic protein receptor II gene (BMPR2) in children with pulmonary arterial hypertension. *Circ J* **72**, 127-133 (2008).

8. Graf, S. *et al.* Identification of rare sequence variation underlying heritable pulmonary arterial hypertension. *Nat Commun* **9**, 1416 (2018).

9. Piao, C. *et al.* Identification of multiple ACVRL1 mutations in patients with pulmonary arterial hypertension by targeted exome capture. *Clin Sci (Lond)* **130**, 1559-1569 (2016).

10. Kataoka, M. *et al.* Alu-mediated nonallelic homologous and nonhomologous recombination in the BMPR2 gene in heritable pulmonary arterial hypertension. *Genet Med* **15**, 941-947 (2013).

11. Higasa, K. *et al.* A burden of rare variants in BMPR2 and KCNK3 contributes to a risk of familial pulmonary arterial hypertension. *BMC Pulm Med* **17**, 57 (2017).

12. Abou Hassan, O.K. *et al.* Clinical and genetic characteristics of pulmonary arterial hypertension in Lebanon. *BMC Med Genet* **19**, 89 (2018).

13. Morisaki, H. *et al.* BMPR2 mutations found in Japanese patients with familial and sporadic primary pulmonary hypertension. *Hum Mutat* **23**, 632 (2004).

14. Kerstjens-Frederikse, W.S. *et al.* TBX4 mutations (small patella syndrome) are associated with childhood-onset pulmonary arterial hypertension. *J Med Genet* **50**, 500-506 (2013).

15. Harrison, R.E. *et al.* Transforming growth factor-beta receptor mutations and pulmonary arterial hypertension in childhood. *Circulation* **111**, 435-441 (2005).

16. Girerd, B. *et al.* Absence of influence of gender and BMPR2 mutation type on clinical phenotypes of pulmonary arterial hypertension. *Respir Res* **11**, 73 (2010).

17. Wang, H. *et al.* Identities and frequencies of BMPR2 mutations in Chinese patients with idiopathic pulmonary arterial hypertension. *Clin Genet* **77**, 189-192 (2010).

18. Machado, R.D. *et al.* Mutations of the TGF-beta type II receptor BMPR2 in pulmonary arterial hypertension. *Hum Mutat* **27**, 121-132 (2006).

19. Zhu, N. *et al.* Rare variants in SOX17 are associated with pulmonary arterial hypertension with congenital heart disease. *Genome Med* **10**, 56 (2018).

20. Elliott, C.G. *et al.* Relationship of BMPR2 mutations to vasoreactivity in pulmonary arterial hypertension. *Circulation* **113**, 2509-2515 (2006).

21. Machado, R.D. *et al.* Genetics and genomics of pulmonary arterial hypertension. *J Am Coll Cardiol* **54**, S32-42 (2009).

22. Thomson, J.R. *et al.* Sporadic primary pulmonary hypertension is associated with germline mutations of the gene encoding BMPR-II, a receptor member of the TGF-beta family. *J Med Genet* **37**, 741-745 (2000).

23. Machado, R.D. *et al.* BMPR2 haploinsufficiency as the inherited molecular mechanism for primary pulmonary hypertension. *American journal of human genetics* **68**, 92-102 (2001).

24. Pfarr, N. *et al.* Hemodynamic and clinical onset in patients with hereditary pulmonary arterial hypertension and BMPR2 mutations. *Respir Res* **12**, 99 (2011).

25. International, P.P.H.C. *et al.* Heterozygous germline mutations in BMPR2, encoding a TGF-beta receptor, cause familial primary pulmonary hypertension. *Nat Genet* **26**, 81-84 (2000).

26. Liu, D. *et al.* Molecular genetics and clinical features of Chinese idiopathic and heritable pulmonary arterial hypertension patients. *Eur Respir J* **39**, 597-603 (2012).

27. Momose, Y. *et al.* De novo mutations in the BMPR2 gene in patients with heritable pulmonary arterial hypertension. *Ann Hum Genet* **79**, 85-91 (2015).

28. Sztrymf, B. *et al.* Clinical outcomes of pulmonary arterial hypertension in carriers of BMPR2 mutation. *Am J Respir Crit Care Med* **177**, 1377-1383 (2008).

29. Humbert, M. *et al.* BMPR2 germline mutations in pulmonary hypertension associated with fenfluramine derivatives. *Eur Respir J* **20**, 518-523 (2002).

30. Portillo, K. *et al.* [Study of the BMPR2 gene in patients with pulmonary arterial hypertension]. *Arch Bronconeumol* **46**, 129-134 (2010).

31. Pousada, G., Baloira, A. & Valverde, D. Complex inheritance in Pulmonary Arterial Hypertension patients with several mutations. *Sci Rep* **6**, 33570 (2016).

32. Koehler, R. *et al.* Low frequency of BMPR2 mutations in a German cohort of patients with sporadic idiopathic pulmonary arterial hypertension. *J Med Genet* **41**, e127 (2004).

33. Zhicheng, J. *et al.* Bone morphogenetic protein receptor-II mutation Arg491Trp causes malignant phenotype of familial primary pulmonary hypertension. *Biochem Biophys Res Commun* **315**, 1033-1038 (2004).

34. Sankelo, M. *et al.* BMPR2 mutations have short lifetime expectancy in primary pulmonary hypertension. *Hum Mutat* **26**, 119-124 (2005).

35. Deng, Z. *et al.* Familial primary pulmonary hypertension (gene PPH1) is caused by mutations in the bone morphogenetic protein receptor-II gene. *Am J Hum Genet* **67**, 737-744 (2000).

36. Morrell, N.W. *et al.* Altered growth responses of pulmonary artery smooth muscle cells from patients with primary pulmonary hypertension to transforming growth factor-beta(1) and bone morphogenetic proteins. *Circulation* **104**, 790-795 (2001).

37. Jing, Z.C. *et al.* [Clinical and genetic characteristics of a Chinese family of primary pulmonary hypertension]. *Zhonghua Yi Xue Za Zhi* **84**, 199-202 (2004).

38. Sugiyama, S. *et al.* Novel insertional mutation in the bone morphogenetic protein receptor type II associated with sporadic primary pulmonary hypertension. *Circ J* **68**, 592-594 (2004).

39. Aldred, M.A. *et al.* BMPR2 gene rearrangements account for a significant proportion of mutations in familial and idiopathic pulmonary arterial hypertension. *Hum Mutat* **27**, 212-213 (2006).

40. Rosenzweig, E.B. *et al.* Clinical implications of determining BMPR2 mutation status in a large cohort of children and adults with pulmonary arterial hypertension. *J Heart Lung Transplant* **27**, 668-674 (2008).

41. Cogan, J.D. *et al.* High frequency of BMPR2 exonic deletions/duplications in familial pulmonary arterial hypertension. *Am J Respir Crit Care Med* **174**, 590-598 (2006).

42. Cogan, J.D. *et al.* Gross BMPR2 gene rearrangements constitute a new cause for primary pulmonary hypertension. *Genet Med* **7**, 169-174 (2005).

43. Best, D.H. *et al.* EIF2AK4 mutations in pulmonary capillary hemangiomatosis. *Chest* **145**, 231-236 (2014).

44. Bossler, A.D., Richards, J., George, C., Godmilow, L. & Ganguly, A. Novel mutations in ENG and ACVRL1 identified in a series of 200 individuals undergoing clinical genetic testing for hereditary hemorrhagic telangiectasia (HHT): correlation of genotype with phenotype. *Hum Mutat* **27**, 667-675 (2006).

45. Ma, L. *et al.* A novel channelopathy in pulmonary arterial hypertension. *N Engl J Med* **369**, 351-361 (2013).

46. Bohnen, M.S. *et al.* Loss-of-Function ABCC8 Mutations in Pulmonary Arterial Hypertension. *Circ Genom Precis Med* **11**, e002087 (2018).

47. Wang, G. *et al.* Novel homozygous BMP9 nonsense mutation causes pulmonary arterial hypertension: a case report. *BMC Pulm Med* **16**, 17 (2016).
