# Supplementary material for: Novel risk genes and mechanisms implicated by exome sequencing of 2572 individuals with pulmonary arterial hypertension
Source: Genome Med. 2019 Nov 14;11:69. doi: 10.1186/s13073-019-0685-z (PMC6857288; doi:10.1186/s13073-019-0685-z)
Supplement: Supplementary file 6 — Additional file 6: Figure S3. Locations of rare deleterious PAH patient-derived BMPR2 variants within the two-dimensional protein structure. [file 13073_2019_685_MOESM6_ESM.pdf]

Figure S3. Locations of rare deleterious PAH patient-derived *BM* *PR2* variants within the two-dimensional protein structure. Predicted damaging missense (D-Mis) variants are shown above the protein schematic; likely-gene-disrupting (LGD including stopgain, frameshift, in-frame deletion and whole exon deletion) variants are below the schematic. The vertical gray lines indicate exon borders. E1(2) indicates a deletion of exon 1 identified in two cases; similar designations are used for the other whole exon deletions.

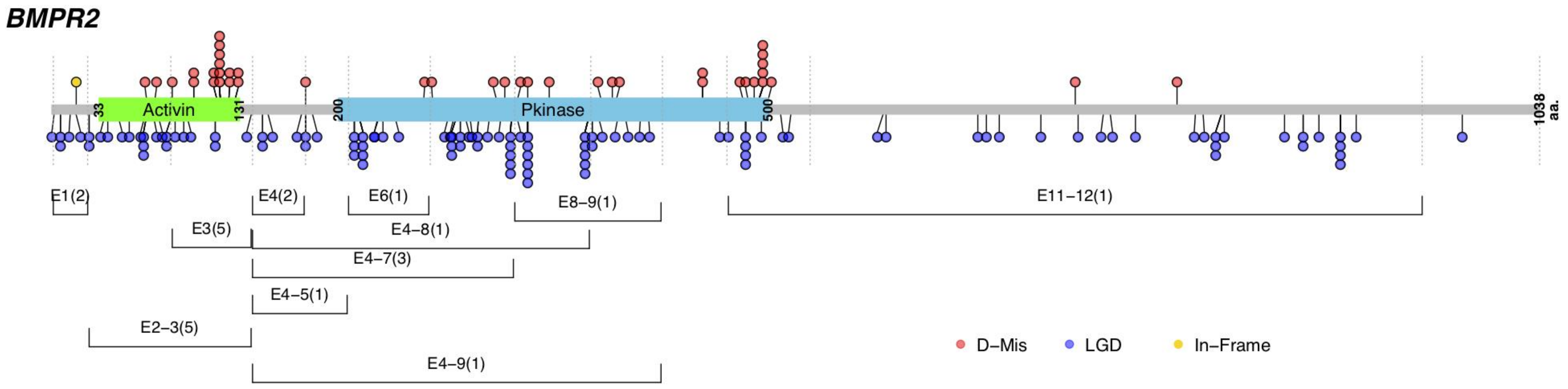

BMPR2
Activin
Pkinase
33
131
200
500
1038
aa.
E1(2)
E4(2)
E6(1)
E8−9(1)
E11−12(1)
E3(5)
E4−8(1)
E4−7(3)
E4−5(1)
E2−3(5)
E4−9(1)
D−Mis
LGD
In−Frame
