# Supplementary material for: Novel risk genes and mechanisms implicated by exome sequencing of 2572 individuals with pulmonary arterial hypertension
Source: Genome Med. 2019 Nov 14;11:69. doi: 10.1186/s13073-019-0685-z (PMC6857288; doi:10.1186/s13073-019-0685-z)
Supplement: Supplementary file 7 — Additional file 7: Figure S4. Locations of rare deleterious PAH patient-derived other previously reported PAH risk gene variants within the two-dimensional protein structures. [file 13073_2019_685_MOESM7_ESM.pdf]

Figure S4. Locations of rare deleterious PAH patient-derived other previously reported PAH risk gene variants within the two-dimensional protein structures. Predicted damaging missense (D-Mis) variants are shown above the protein schematics; likely-gene-disrupting (LGD including stopgain, frameshift, in-frame deletion and whole exon deletion) variants are shown below the schematics. The vertical gray lines indicate exon borders. For *ACVRL1*, E10 (1) indicates a deletion of exon 10 identified in one case. For *EIF2AK4*, patients 12-064 and 21-036 are homozygous for the given *EIF2AK4* variants, and patients 12-014, 02-030 and 10-091 are compound heterozygotes.

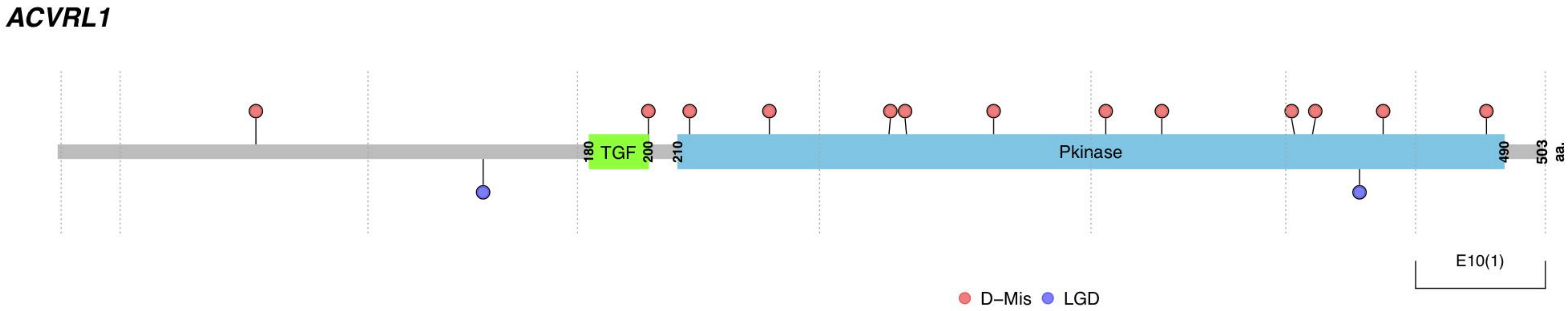
ACVRL1
180
TGF
200
210
Pkinase
490
503
aa.
E10(1)
D−Mis
LGD

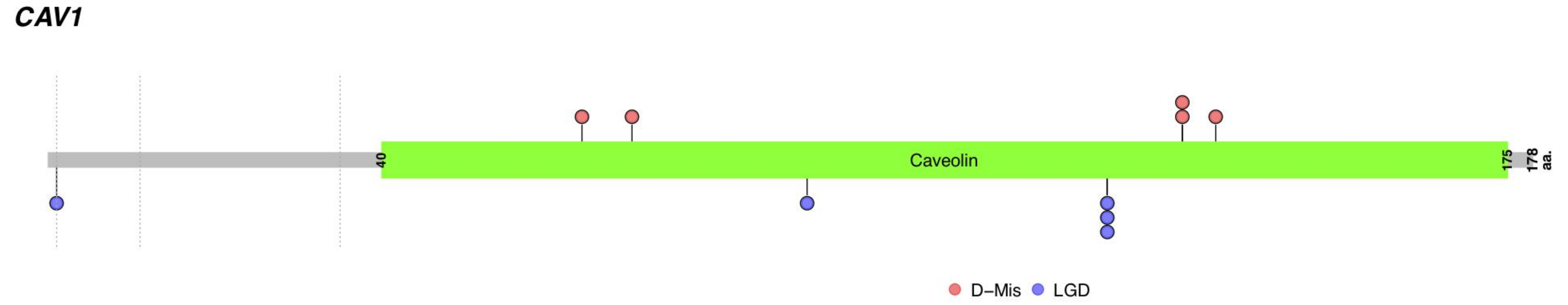
CAV1
40
Caveolin
175
178
aa.
D−Mis
LGD

BMPR1A

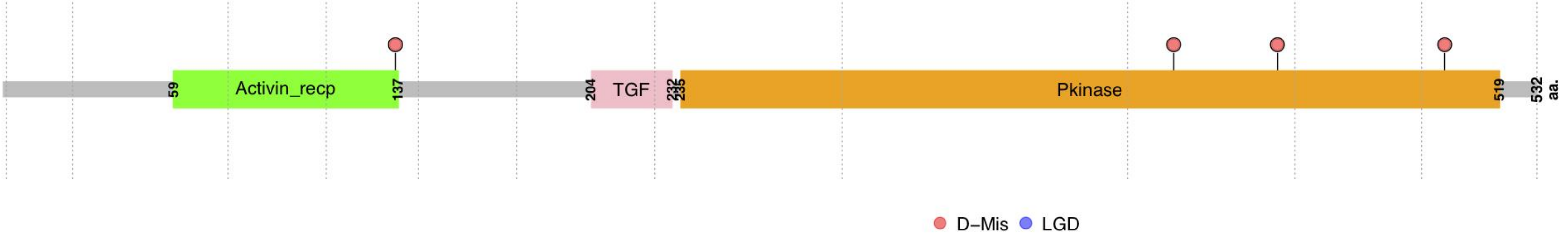

BMPR1B

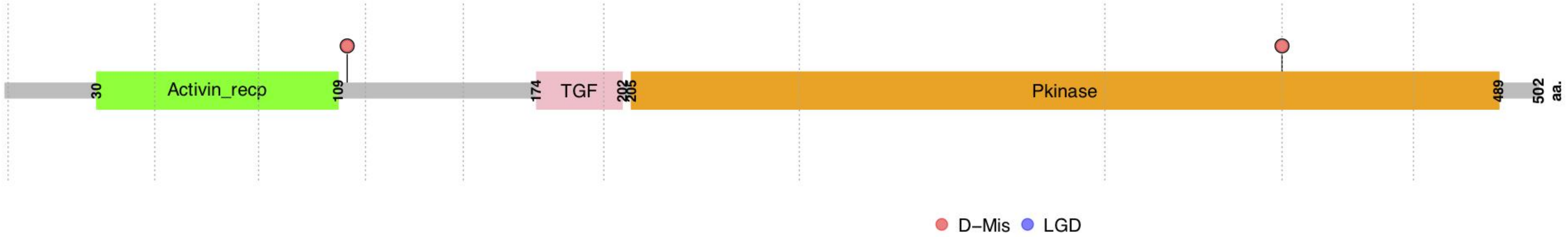

EIF2AK4

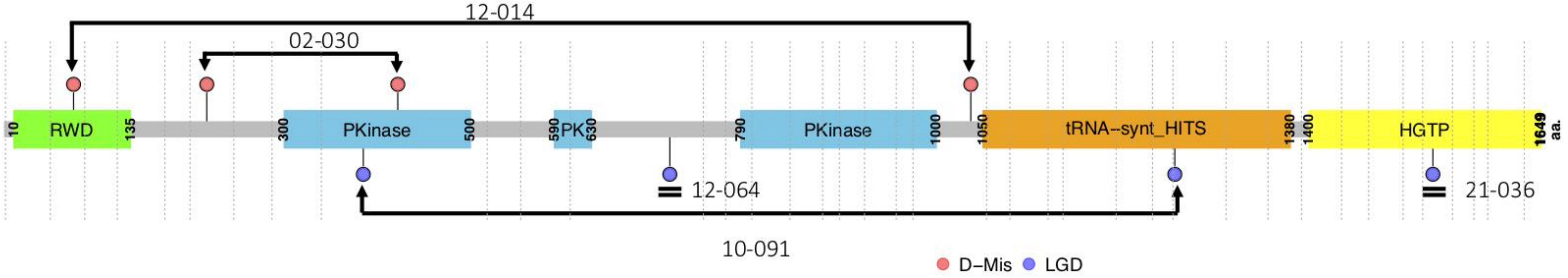

ENG

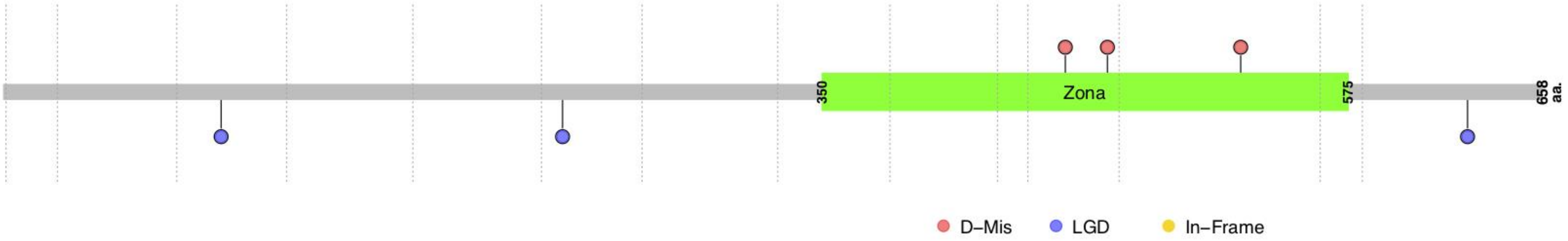

KCNK3

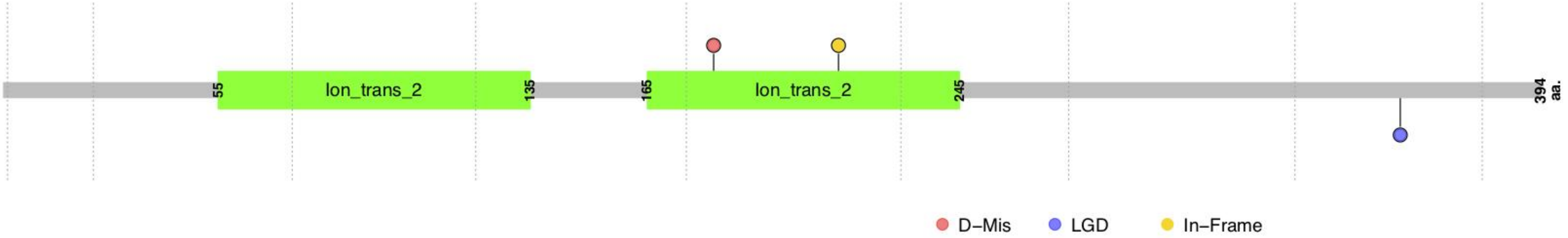

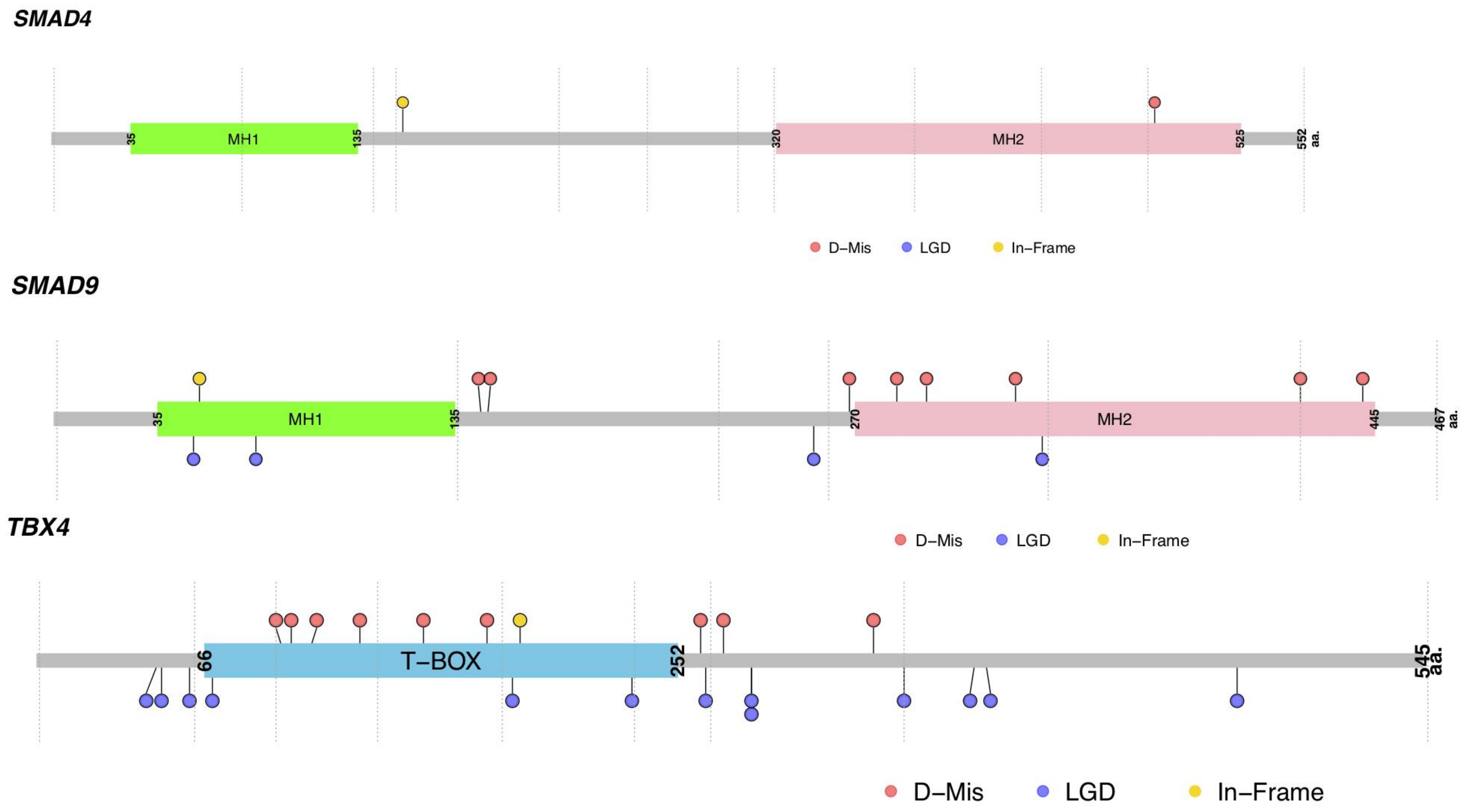
SMAD4
MH1
MH2
35
135
320
525
552 aa.
D–Mis
LGD
In–Frame
SMAD9
MH1
MH2
35
135
270
445
467 aa.
D–Mis
LGD
In–Frame
TBX4
T–BOX
66
252
545 aa.
D–Mis
LGD
In–Frame

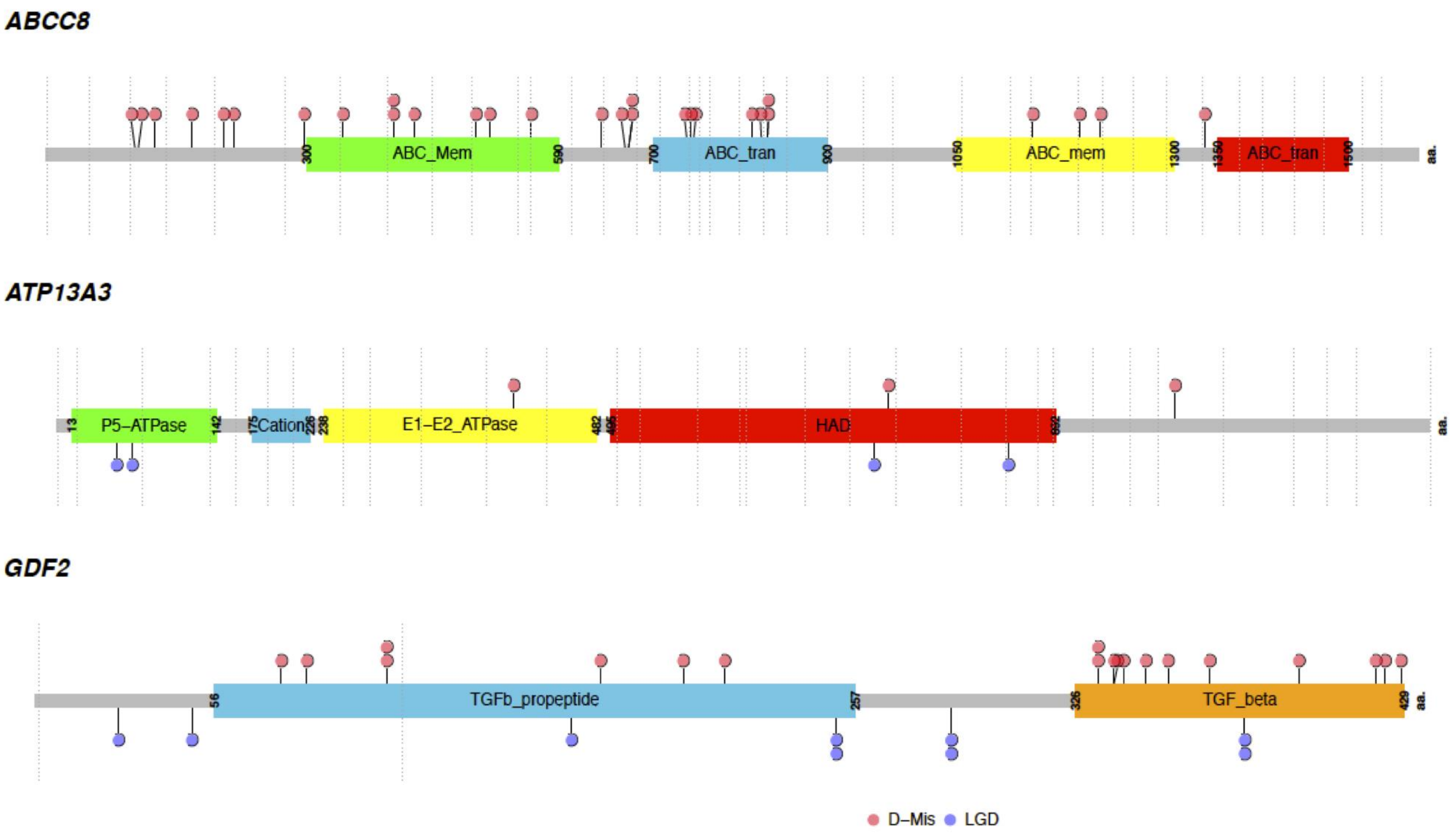

ABCC8
ABC_Mem
ABC_tran
ABC_mem
ABC_tran
300
590
700
900
1050
1300
1350
1500
aa.
ATP13A3
P5−ATPase
Cation
E1−E2_ATPase
HAD
13
142
175
226
238
482
495
692
aa.
GDF2
TGFb_propeptide
TGF_beta
56
257
326
429
aa.
D−Mis
LGD

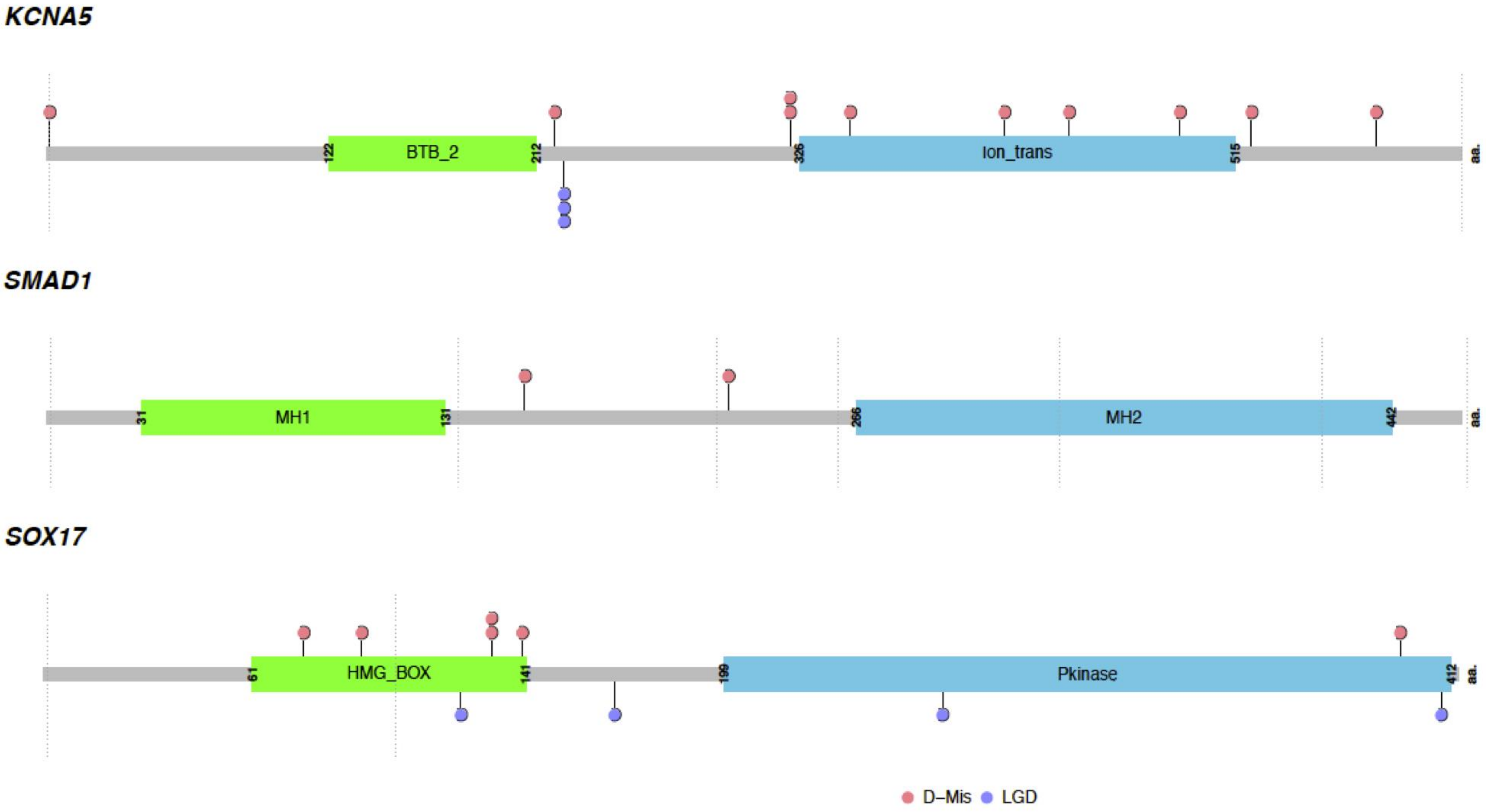
KCNA5
122
BTB_2
212
326
Ion_trans
515
aa.
SMAD1
31
MH1
131
266
MH2
442
aa.
SOX17
61
HMG_BOX
141
199
Pkinase
412
aa.
D–Mis
LGD
