# Supplementary material for: Novel risk genes and mechanisms implicated by exome sequencing of 2572 individuals with pulmonary arterial hypertension
Source: Genome Med. 2019 Nov 14;11:69. doi: 10.1186/s13073-019-0685-z (PMC6857288; doi:10.1186/s13073-019-0685-z)
Supplement: Supplementary file 8 — Additional file 8: Table S5. Similar frequency of rare synonymous variants among European PAH cases and non-Finnish European gnomAD and in-house controls. [file 13073_2019_685_MOESM8_ESM.docx]

**Table S5. Similar frequency of rare synonymous variants among European PAH cases and non-Finnish European gnomAD and in-house controls.**

| **Mutation type*** | **PAH cases (n=1,832)** | **controls (n=12,771)** | **Enrichment** | **p-value** |
| --- | --- | --- | --- | --- |
|  |  |  |  |  |
| SYN | 60024 | 420379 | 1.0 | 0.09 |
| LGD | 8198 | 57417 | 1.0 | 0.53 |
| MIS | 122405 | 862011 | 1.0 | 0.52 |

*Syn, synonymous; LGD, likely gene damaging; Mis, missense.
