# Supplementary material for: Novel risk genes and mechanisms implicated by exome sequencing of 2572 individuals with pulmonary arterial hypertension
Source: Genome Med. 2019 Nov 14;11:69. doi: 10.1186/s13073-019-0685-z (PMC6857288; doi:10.1186/s13073-019-0685-z)
Supplement: Supplementary file 9 — Additional file 9: Figure S5. Gene-level burden test for rare synonymous variants using 1832 European cases and 12,771 European controls. [file 13073_2019_685_MOESM9_ESM.pdf]

Figure S5. Gene-level burden test for rare synonymous variants using 1832 European cases and 12,771 European controls. Results of a binomial test confined to rare synonymous variants in 20,000 protein-coding genes.

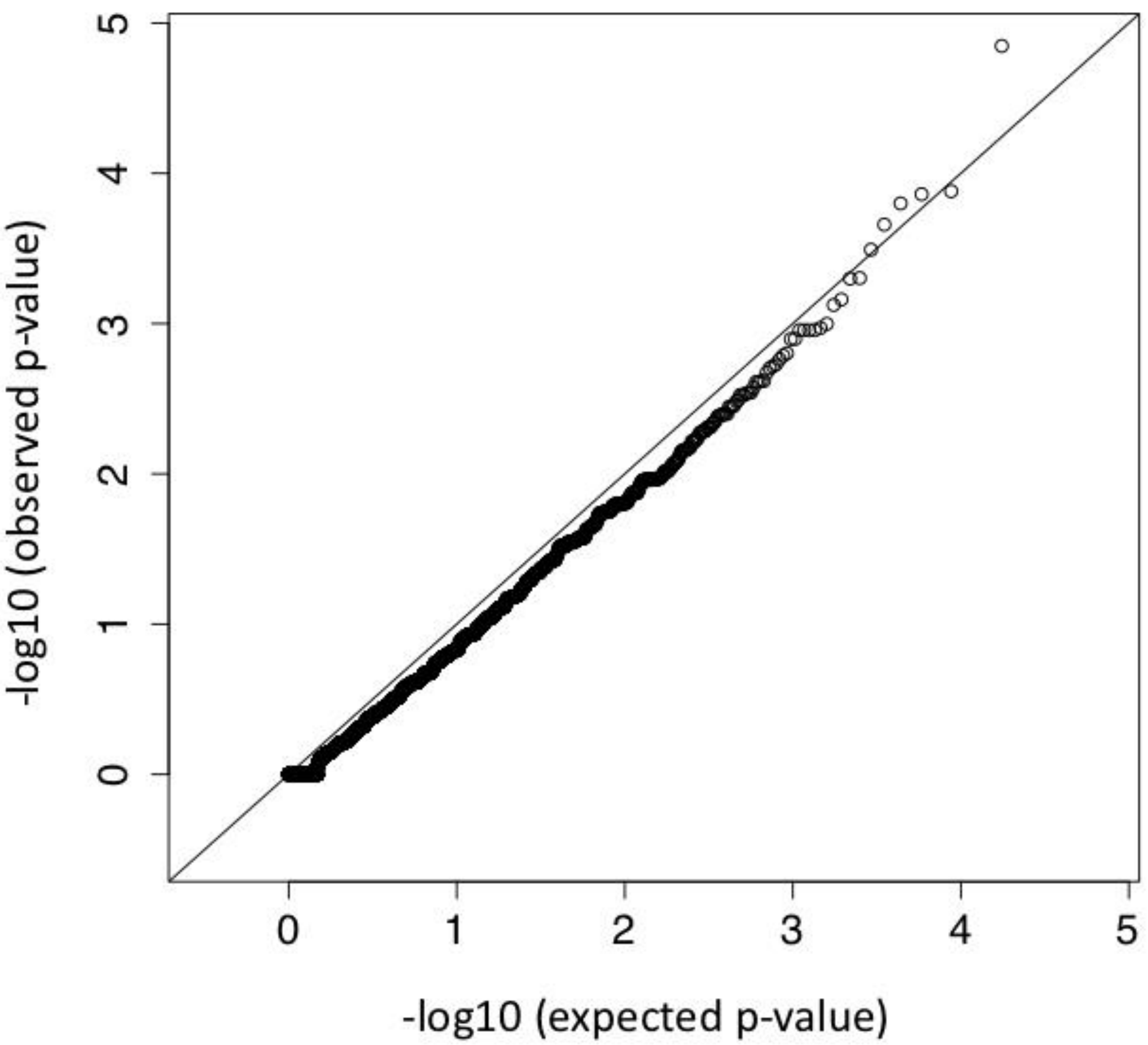

-log10 (observed p-value)
-log10 (expected p-value)
0
1
2
3
4
5
