# Supplementary material for: Novel risk genes and mechanisms implicated by exome sequencing of 2572 individuals with pulmonary arterial hypertension
Source: Genome Med. 2019 Nov 14;11:69. doi: 10.1186/s13073-019-0685-z (PMC6857288; doi:10.1186/s13073-019-0685-z)
Supplement: Supplementary file 10 — Additional file 10: Figure S6. Depth of coding sequence coverage for GGCX and KLK1. [file 13073_2019_685_MOESM10_ESM.pdf]

Figure S6. Depth of coding sequence coverage for *G* [illegible] *GCX* and *KLK1*.

Comparison of coverage between PAH Biobank cases and gnomAD WGS controls at read depth >10X or >15X.

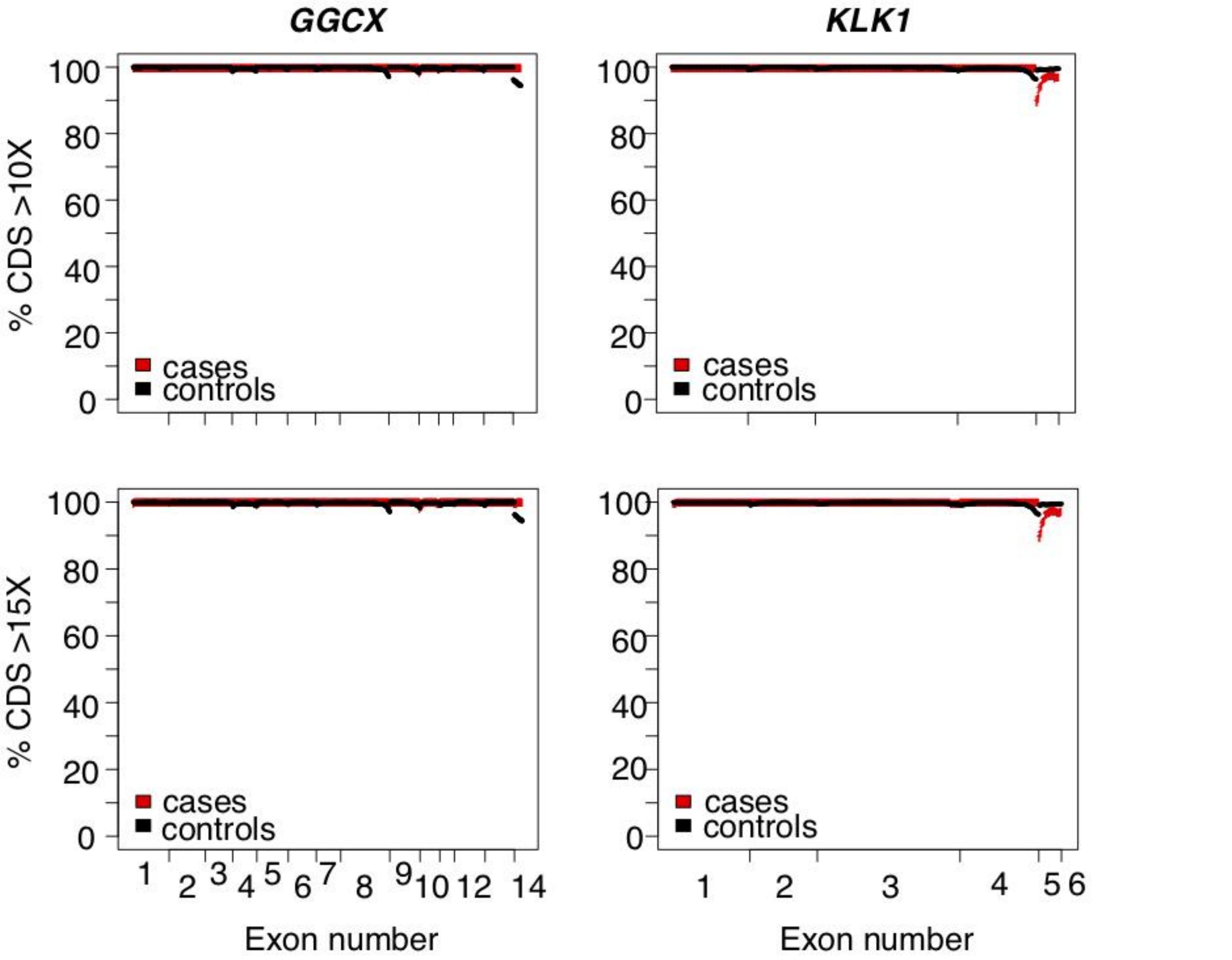
GGCX
KLK1
% CDS >10X
% CDS >15X
100
80
60
40
20
0
cases
controls
1 2 3 4 5 6 7 8 9 10 12 14
1 2 3 4 5 6
Exon number
