# Supplementary material for: Novel risk genes and mechanisms implicated by exome sequencing of 2572 individuals with pulmonary arterial hypertension
Source: Genome Med. 2019 Nov 14;11:69. doi: 10.1186/s13073-019-0685-z (PMC6857288; doi:10.1186/s13073-019-0685-z)
Supplement: Supplementary file 11 — Additional file 11: Table S6A. Enrichment of rare deleterious variants in a KLK gene-set expressed in lung among 1832 European PAH cases and 12,771 European controls; Table S6B. Association analysis of KLK genes expressed in lung using 1832 (all PAH) or 812 (IPAH) European cases and 12,771 European controls. [file 13073_2019_685_MOESM11_ESM.docx]

**Table S6A. Enrichment of rare deleterious variants in a *KLK* gene-set* expressed in lung among 1,832 European PAH cases and 12,771 European controls.**

| **Mutation type**** | **PAH cases (n=1,832)** | **Controls (n=12,771)** | **Enrichment** | **p-value** |
| --- | --- | --- | --- | --- |
|  |  |  |  |  |
| LGD | 5 | 12 | 2.9 | 0.05 |
| D-Mis | 16 | 56 | 2.0 | 0.02 |
| LGD+D-Mis | 21 | 69 | 2.1 | 0.004 |

*KLK gene-set: KLK1, KLK5, KLK6, KLK7, KLK10, KLK11, KLK12, KLK13, KLK14.

**LGD, likely gene damaging; D-Mis, missense with REVEL variable threshold.

**Table S6B. Association analysis of *KLK* genes expressed in lung using 1,832 (all PAH) or 812 (IPAH) European cases and 12,771 European controls.**

| **Gene** | **PAH subclass** | **Empirical Revel** | **P-value** | **Number of permutations** | **Permutation p-value** | **OR** |
| --- | --- | --- | --- | --- | --- | --- |
| *KLK1* | all PAH | 0.45 | 1.35E-07 | 10000000 | 2.00E-07 | 13.9 |
| *KLK12* | all PAH | 0.7 | 0.03 | 1000 | 0.07 | 7.0 |
| *KLK10* | all PAH | 0.25 | 0.04 | 1000 | 0.09 | 2.4 |
| *KLK13* | all PAH | 0.25 | 0.10 | 1000 | 0.13 | 2.3 |
| *KLK5* | all PAH | 0.4 | 0.33 | 1000 | 0.44 | 1.6 |
| *KLK14* | all PAH | 0.4 | 0.31 | 1000 | 0.46 | 2.0 |
| *KLK6* | all PAH | 0.85 | 0.42 | 1000 | 0.55 | 2.3 |
| *KLK7* | all PAH | 0.2 | 0.35 | 1000 | 0.59 | 1.3 |
| *KLK11* | all PAH | 0.25 | 0.82 | 1000 | 0.89 | 0.6 |
|  |  |  |  |  |  |  |
| *KLK1* | IPAH | 0.45 | 3.34E-09 | 10000000 | 1.00E-07 | 26.2 |
| *KLK12* | IPAH | 0.7 | 0.004 | 1000 | 0.007 | 15.7 |
| *KLK13* | IPAH | 0.3 | 0.024 | 1000 | 0.032 | 4.2 |
| *KLK10* | IPAH | 0.25 | 0.052 | 1000 | 0.076 | 3.1 |
| *KLK5* | IPAH | 0.4 | 0.225 | 1000 | 0.339 | 2.4 |
| *KLK14* | IPAH | 0.6 | 0.309 | 1000 | 0.412 | 3.1 |
| *KLK11* | IPAH | 0.25 | 0.758 | 1000 | 0.742 | 0.7 |
| *KLK7* | IPAH | 0.25 | 0.772 | 1000 | 0.767 | 0.7 |
| *KLK6* | IPAH | 0.8 | 1 | 1000 | 1 | 2.0 |
