# Supplementary material for: Novel risk genes and mechanisms implicated by exome sequencing of 2572 individuals with pulmonary arterial hypertension
Source: Genome Med. 2019 Nov 14;11:69. doi: 10.1186/s13073-019-0685-z (PMC6857288; doi:10.1186/s13073-019-0685-z)
Supplement: Supplementary file 12 — Additional file 12: Table S7. Mean clinical phenotypes of KLK1 and GGCX IPAH cases compared to other IPAH cases without variants in known risk genes. [file 13073_2019_685_MOESM12_ESM.docx]

**Table S7. Mean clinical phenotypes of *KLK1* and *GGCX* IPAH cases compared to other IPAH cases without variants in known risk genes*.**

| **Group** | **Age dx (y)** | **MPAP (mmHg)** | **MPCW (mmHg)** | **CO, Fick (L/min)** | **PVR (Woods units)** | **MAP (mmHg)** | **MAP:MPAP** |
| --- | --- | --- | --- | --- | --- | --- | --- |
|  |  |  |  |  |  |  |  |
| ***KLK1*** | 48 ± 19 | 46 ± 12 | 10 ± 3 | 4.7 ± 1.7 | 8.2 ± 4.7 | 99 ± 7 | 2.4 ± 0.4 |
| **(n)** | (10) | (10) | (10) | (8) | (8) | (7) | (7) |
| ***GGCX*** | 48 ± 15 | 57 ± 14 | 11 ± 4 | 4.5 ± 1.4 | 10.7 ± 8.0 | 88 ± 17** | 1.6 ± 0.6 |
| **(n)** | (17) | (17) | (15) | (12) | (10) | (10) | (10) |
| **other IPAH** | 48 ± 19 | 52 ± 12 | 12 ± 5 | 7.0 ± 3.2 | 11.3 ± 7.2 | 96 ± 15 | 1.9 ± 0.7 |
| **(n)** | (1096) | (1043) | (1016) | (715) | (731) | (582) | (582) |

Abbreviations: Age dx, patient age at diagnosis; MPAP, mean pulmonary arterial pressure; MPCW, mean pulmonary capillary wedge pressure; CO, cardiac output by Frick method; PVR, pulmonary vascular resistance; MAP, mean arterial pressure.

*known risk genes included 18 genes from Supplementary Tables 3 and 4.

**p<0.02 vs other IPAH, Student's t-test.
