# Supplementary material for: Novel risk genes and mechanisms implicated by exome sequencing of 2572 individuals with pulmonary arterial hypertension
Source: Genome Med. 2019 Nov 14;11:69. doi: 10.1186/s13073-019-0685-z (PMC6857288; doi:10.1186/s13073-019-0685-z)
Supplement: Supplementary file 13 — Additional file 13: Table S8. Lack of enrichment of KLK1 common SNP, R77H, in the PAH Biobank cohort compared to gnomAD population data. [file 13073_2019_685_MOESM13_ESM.docx]

**Table S8. Lack of enrichment of *KLK1* common SNP, R77H, in the PAH Biobank cohort compared to gnomAD population data.**

| **Ancestry group** | **# R77H alleles observed in cases** | **# Total cases** | **R77H AF in gnomad** | **p-value** | **RR** |
| --- | --- | --- | --- | --- | --- |
|  |  |  |  |  |  |
| NFE | 136 | 1,841 | 0.0371 | 1.00 | 1.0 |
| AFR | 54 | 291 | 0.1033 | 0.54 | 0.9 |

NFE, non-Finnish European; AFR, African; AF, allele frequency; RR, relative risk.

P-value based on binomial test.
